# Hydrazine-Containing Heterocycle Cytochalasan Derivatives From Hydrazinolysis of Extracts of a Desert Soil-Derived Fungus *Chaetomium madrasense* 375

**DOI:** 10.3389/fchem.2021.620589

**Published:** 2021-04-21

**Authors:** Qingfeng Guo, Jinhua Chen, Yuwei Ren, Zhenhua Yin, Juanjuan Zhang, Baocheng Yang, Xuewei Wang, Wenbing Yin, Wancun Zhang, Gang Ding, Lin Chen

**Affiliations:** ^1^Zhengzhou Key Laboratory of Synthetic Biology of Natural Products, Zhengzhou Key Laboratory of Medicinal Resources Research, Comprehensive Utilization of Edible and Medicinal Plant Resources Engineering Technology Research Center, Huanghe Science and Technology College, Zhengzhou, China; ^2^Department of Pharmacy, Affiliated Cancer Hospital of Zhengzhou University, Henan Cancer Hospital, Zhengzhou, China; ^3^State Key Laboratory of Mycology, Institute of Microbiology, Chinese Academy of Sciences, Beijing, China; ^4^Henan Key Laboratory of Children's Genetics and Metabolic Diseases, Children's Hospital Affiliated to Zhengzhou University, Zhengzhou, China; ^5^Institute of Medicinal Plant Development, Chinese Academy of Medical Science and Union Medical College, Beijing, China

**Keywords:** diversity-enhanced, cytochalasans, antiproliferative activity, chaetomium madrasense, hydrazinolysis

## Abstract

“Diversity-enhanced extracts” is an effective method of producing chemical libraries for the purpose of drug discovery. Three rare new cytochalasan derivative chaetoglobosins B_1_-B_3_ (**1**–**3**) were obtained from chemically engineered crude broth extracts of *Chaetomium madrasense* 375 prepared by reacting with hydrazine monohydrate and four known metabolite chaetoglobosins (**4–7**) were also identified from the fungus. The structures were identified by NMR and MS analysis and electronic circular dichroism simulation. In addition, the antiproliferative activities of these compounds were also evaluated, and the drug-resistant activities of cytochalasans were evaluated for the first time. Compound **6** possessed potent activity against four human cancer cells (A549, HCC827, SW620, and MDA-MB-231), and two drug-resistant HCC827 cells (Gefitinib-resistant, Osimertinib-resistant) compared with the positive controls.

## Introduction

Natural products have played an important role in the development of novel drugs because of their established structural diversity (Newman and Cragg, 2016). However, research on natural products within the pharmaceutical industry has recently declined, and it has become more and more difficult to obtain compounds bearing skeletally novel structures from natural sources (Bradshaw et al., 2001; Wolfender and Queiroz, 2012). Chemical modification of natural product extracts provides a new strategy for discovering of diverse structurally active molecules. Recently, there have been several reports (Lopez et al., 2007; Mendez et al., 2011; Salazar et al., 2011; Ramallo et al., 2012; Kikuchi et al., 2014; Wu et al., 2015; Garcia et al., 2016) on the application of chemical alteration of natural product extracts to obtain new or bioactive compounds.

Cytochalasans comprise a large group of polyketide synthase and non-ribosomal peptide synthetase-derived fungal metabolites with a wide range of biological activities (Scherlach et al., 2010). The macrocyclic structure of most cytochalasans contains carbonyl functional groups, which could react with hydrazine to form either hydrazones or acyl hydrazides that rarely emerge in nature. This transformation could increase the nitrogen content and nucleophilic character of further reactions (Feher and Schmidt, 2003). In previous studies, we reported several bioactive cytochalasans that were isolated from *C. madrasense* 375 derived from desert soil (Guo et al., 2019). To gain more novel cytochalasan-like bioactive compounds, a chemical transformation of crude broth extracts of *C. madrasense* 375 with hydrazine monohydrate was performed, followed by purification of reaction products, which offered three new cytochalasans derivatives chaetoglobosins B_1_-B_3_ (**1**–**3**), and known chaetoglobosin B (**4**) (Sekita et al., 1982, 1983) chaetoglobosin D (**5**) (Sekita et al., 1982, 1983), chaetoglobosin E (**6**) (Sekita et al., 1976), and cytoglobosin A (**7**) (Cui et al., 2010) were also isolated from the unreactive raw extract of this fungus. In this paper, we present the isolation, structure elucidation, bioactivities, and plausible synthesis pathways of these compounds.

## Materials and Methods

### General Experimental Procedures

CD spectra were determined on a JASCO J-810 spectropolarimeter (JASCO Corporation), and UV data were recorded using a PERSEE UV-VIS spectrophotometer T9 (Beijing, China). NMR experiments were carried out on a Bruker AVANCE III 400 NMR spectrometer (Bruker, Germany), using tetramethylsilane (TMS) or solvent signals as an internal reference. HRESIMS data were collected on an Agilent 6250 TOF LC/MS, and ESIMS data were acquired on an Agilent 1200 series LC/MS system. Semipreparative HPLC was run on a Calmflow^plus^ system that was equipped with a YMC Pack ODS-A column (10 mm × 250 mm 5 μm, Japan) and a 50D UV-vis Detector (Lumiere Tech Ltd) and with a flow rate of 2.0 ml/min. Packing materials for column chromatography were silica gel (200–300 mesh; Qingdao Marine Chemical Factory, Qingdao, China), ODS (YMC, Japan), and Sephadex LH-20 (GE Healthcare BioSciences AB, Sweden). All chemicals used in the study were of analytical grade.

### Fungal Material and Fermentation

The samples of the fungus *C. madrasense*375 (CCTCC M 2019517 CLC375) were collected from a soil sample obtain at Hotan city, XinJiang province, People's Republic of China, and identified by one of the authors (XueWei Wang). The fungus was identified as *C. madrasense* according to its internal transcribed spacer (ITS) sequence of ITS rDNA (Supplementary Figure 34) and beta-tubulin encoding gene (Supplementary Figure 33) from genomic DNA, as well as its morphological features. A phylogenetic tree was constructed based on the sequence of the partial beta-tubulin gene from *C. madrasense* and other species in the genus *Chaetomium*, with *Aspergillus nidulans* as the outgroup (Supplementary Figure 32). The sequence of the strain was deposited in GenBank with accession number KP269060.1. The fungal strain was maintained on potato dextrose agar (PDA) at 25°C for 7 days to prepare the seed culture. Agar plugs were cut into small pieces (approximately 1 cm × 1 cm) and inoculated into four 500 ml Erlenmeyer flasks containing 200 ml of synthetic dropout Medium (SD, peptone 10.0 g/L, dextrose 40.0 g/L) and incubated at 26°C for 10 days on a rotary shaker (120 rpm). The obtained liquid seeds were transferred into fifty 500 ml Erlenmeyer flasks, each containing 200 ml of the sterilized synthetic dropout medium and incubated at 26°C for 35 days on a rotary shaker (120 rpm) before harvest. The culture was filtered to separate broth and mycelia, and then extracted by ethyl acetate (three times) at room temperature. The combined extracts of broth and mycelia were dried under reduced pressure to give a dark brown gum (8.3 g). The crude extract was then suspended in H_2_O and extracted with petroleum ether, EtoAc, and *n*-BuOH, respectively. The EtoAc extract (5.6 g) was packed with cytochalasans based on the analysis of TLC and LC-MS experiments and divided into two parts, one (4 g) prepared for chemical modification, another (1.6 g) for metabolite research directly.

### Chemical Modification of Extraction and Isolation

An EtOH solution of the dry extract (3% wt/vol) containing hydrazine monohydrate (6 % vol/vol) and three drops of hexahydropyridine was stirred under reflux at 75°C for 8 h, and the solvent was removed under reduced pressure. The preliminary experiment was carried out on a 100 mg scale to obtain modified production successfully and prove feasibility. The obtained residue was subjected to an ODS column eluted with CH_3_OH/H_2_O (30, 50, 70, and 100%, *v*/*v*) to afford four main fractions (A-D). Fraction C was chromatographed by Sephadex LH-20 (100% MeOH) to give 10 subfractions (C-1–C-10), Fraction C-1 was further purified *via* semipreparative HPLC (37% MeCN in H_2_O) to yield chaetoglobosin B_1_ (**1**; 2.5 mg, *t*_R_ 16.1 min). Fraction C-3 was further purified by semipreparative HPLC using 32% acetonitrile in water to afford chaetoglobosin B_3_ (**3**; 5.8 mg, *t*_R_ 32.3 min). Chaetoglobosin B_2_ (**2**; 4.3 mg, *t*_R_ 24.6 min) was purified from subfraction C-5 by RP-HPLC using 35% acetonitrile in water. The unreactive raw extract (1.6 g) was subjected to Sephadex LH-20 (CH_2_Cl_2_/MeOH, *v*/*v*, 1:1) to yield five fractions (Fr1–Fr5). Fraction 3 was separated using semipreparative HPLC (45% MeCN in H_2_O with 0.1% HCOOH) to give chaetoglobosin B (**4**; 8.7 mg, *t*_R_ 31.5 min), chaetoglobosin D (**5**; 6.3 mg, *t*_R_ 24.9 min), and chaetoglobosin E (**6**; 4.6 mg, *t*_R_ 21.7 min). Cytoglobosin A (**7**; 1.3 mg, *t*_R_ 19.4 min) was obtained from fraction 4 using semipreparative HPLC (50% MeCN in H_2_O with 0.1% HCOOH).

Chaetoglobosin B_1_ (**1**): white amorphous powder; UV (MeOH) λ_max_ (log ε) 281 nm (2.00), 225 nm (3.02), and 221 nm (3.01); for ^1^H NMR (400 MHz) and ^13^C NMR (100 MHz) data, see Tables 1, 2; HR-ESI-MS *m/z* 543.2950 [M+H]^+^ (calcd for C_32_H_39_N_4_O_4_, 543.2966).

**Table 1:** ^1^H NMR spectroscopic data (δ in ppm, *J* in Hz) for compounds **1**–**3**.

**No**.	**1^a^**	**2^b^**	**3^c^**
1′		10.89 (1H, br s)	8.42 (1H, s)
2′	6.89 (1H, br s)	6.98 (1H, br s)	7.27 (1H, br s)
4′	7.38 (1H, d, 7.7)	7.28 (1H, d, 7.9)	7.46 (1H, d, 7.6)
5′	6.99 (1H, t, 7.2)	6.95 (1H, t, 7.5)	6.97 (1H, t, 7.2)
6′	7.06 (1H, t, 6.9)	7.05 (1H, t,7.5)	7.06 (1H, t, 6.9)
7′	7.31(1H, d, 8.1)	7.33 (1H, d, 8.0)	7.35 (1H, d, 8.1)
2		7.82 (1H, br s)	
3	3.58 (1H, dd, 9.8, 5.2)	3.55 (1H, t, 6.0)	3.38 (1H, m)
4	3.33 (1H, s)	3.28 (1H, br s)	3.82 (1H, s)
7	3.94 (1H, d, 9.3)	3.63 (1H, br s)	3.73 (1H, d, 8.8)
8	2.32 (1H, m)	1.06 (1H, m)	1.67 (1H, t, 9.7)
10	2.73 (1H, dd, 13.9, 5.2)	2.64 (1H, dd, 14.2, 5.5)	2.68 (1H, m)
	2.39 (1H, dd, 13.6, 9.9)	2.34 (1H, dd, 14.0, 9.2)	2.60 (1H, m)
11	1.16 (3H, s)	1.27 (3H, s)	1.00 (3H, s)
12	1.64 (3H, s)	1.56 (3H, s)	1.52 (3H, s)
13	5.91 (1H, dd, 14.2, 10.3)	1.80 (1H, m)	6.25 (1H, dd, 14.6, 10.6)
		1.43 (1H, m)	
14	4.79 (1H, m)	0.30 (1H, m)	4.56 (1H, m)
		1.20 (1H, m)	
15	2.11 (1H, d, 11.0)	0.64 (1H, m)	2.20 (1H, m)
	1.73 (1H, d, 13.7)	0.22 (1H, m)	1.99 (1H, m)
16	2.23 (1H, m)	1.15 (1H, m)	2.36 (1H, m)
17	4.75 (1H, m)	1.05 (1H, m)	5.40 (1H, d, 9.4)
		0.76 (1H,m)	
18		1.78 (1H, m)	
19	4.11 (1H, d, 3.9)	5.05 (1H, s)	5.10 (1H, s)
20	5.17(1H, d, 4.0)		
21		7.76 (1H,d, 8.7)	
22	5.98 (1H, s)	8.00 (1H, d, 8.9)	6.39 (1H, s)
16-Me	0.87 (3H, d, 6.8)	0.72 (3H, d, 6.8)	0.94 (3H, d, 6.8)
18-Me	1.69 (3H, s)	1.21(3H, d, 6.8)	1.35 (3H, s)

a *Measured in CD_3_OD*.

b *Measured in DMSO-d_6_*.

c *Measured in DMSO-d_6_ with one drop CD_3_OD*.

**Table 2:** ^13^C NMR spectroscopic data (δ in ppm) for compounds **1**–**3**.

**NO**	**1^a^**	**2^b^**	**3^c^**
1′a	138.1 C	136.2 C	136.2 C
2′	124.6 CH	123.1 CH	123.8 CH
3′	112.1 C	110.5 C	110.7 C
3′a	128.6 C	127.0 C	127.5 C
4′	119.3 CH	118.0 CH	118.2 CH
5′	119.8 CH	118.3 CH	118.3 CH
6′	122.3 CH	121.0 CH	120.9 CH
7′	112.3 CH	111.4 CH	111.5 CH
1	179.2 C	173.8 C	175.8 C
3	60.1 CH	56.4 CH	58.3 CH
4	53.6 CH	50.9 CH	47.1 CH
5	128.4 C	125.3 C	126.1 C
6	134.3 C	134.4 C	132.6 C
7	71.2 CH	69.8 CH	68.7 CH
8	56.4 CH	50.3 CH	55.8 CH
9	51.5 C	53.0 C	49.1 C
10	32.5 CH_2_	31.9 CH_2_	31.9 CH_2_
11	17.4 CH_3_	17.1 CH_3_	16.9 CH_3_
12	14.7 CH_3_	15.2 CH_3_	14.4 CH_3_
13	129.9 CH	25.2 CH_2_	130.7 CH
14	135.1CH	27.7 CH_2_	133.1 CH
15	43.0 CH	34.6 CH_2_	42.3 CH_2_
16	33.1CH	31.5 CH	32.4 CH
17	137.8 CH	30.2 CH_2_	136.5 CH
18	133.5 C	34.4 CH	133.4 C
19	82.4 CH	75.8 CH	75.6 CH
20	73.0 CH	165.5 C	143.4 C
21	147 C	123.5 CH	128.9 C
22	102.6 CH	125.1 CH	105.2 CH
23	155 C	163.0 C	152.0 C
16-Me	21.8 CH_3_	21.0 CH_3_	21.6 CH_3_
18-Me	12.7 CH_3_	21.9 CH_3_	9.6 CH_3_

a *Measured in CD_3_OD*.

b *Measured in DMSO-d_6_*.

c *Measured in DMSO-d_6_ with one drop CD_3_OD*.

Chaetoglobosin B_2_ (**2**): white amorphous powder; UV (MeOH) λ_max_ (log ε) 279 nm (1.21) and 211 nm (3.23); for ^1^H NMR (400 MHz), and ^13^C NMR (100 MHz) data, see Tables 1, 2; HR-ESI-MS *m/z* 529.3184 [M+H]^+^ (calcd for C_32_H_41_N_4_O_3_, 529.3173).

Chaetoglobosin B_3_ (**3**): white amorphous powder; UV (MeOH) λ_max_ (log ε) 282 nm (0.31), and 221 nm (1.57); ECD (MeOH) λ_max_ (Δε) = 286 nm (+14.5), 224 (−27.2); for ^1^H NMR (400 MHz) and ^13^C NMR (100 MHz) data, see Tables 1, 2; HR-ESI-MS *m/z* 567.2738 [M+H]^+^ (calcd for C_32_H_35_N_6_O_4_, 567.2714).

### Quantum-Chemical Calculation

Monte Carlo conformational searches were run with the Spartan 14 software using the Merck Molecular Force Field (MMFF). The Selected conformers, which account for more than 1% of the Boltzmann distribution, were initially optimized at the B3LYP/6-31+G (d, p) level with the conductor-like polarizable continuum model (CPCM) conductor calculation in methanol solvent. The conformers of **3** were identified *via* ECD simulation using the time-dependent density functional theory (TD-DFT) method at the B3LYP/6-31+G (d, p) level with methanol as solvent, and the rotational strengths of 30 excited states were calculated. ECD spectra of compound **3** were obtained using the SpecDis 1.6 (University of Würzburg, Würzburg, Germany) and GraphPad Prism 5 (University of California San Diego, USA) software by applying Gaussian band shapes with sigma = 0.3 eV from dipole-length rotational strengths (Guo et al., 2019).

### Antiproliferative Assay

Antiproliferative activity was performed against four human cancer cell lines (A549, SW620, MDA-MB-231, and HCC827), together with two drug-resistant (gefitinib, osimertinib) HCC827cell lines, applying the 3-(4,5-dimethyl-2-thiazolyl)-2,5-diphenyl-2-H-tetrazolium bromide (MTT) method (Chen et al., 2014) with the DDP (cisplatin, Sigma), gefitinib, and osimertinib as positive controls, respectively. The cell lines were cultured in RPMI-1640 medium supplemented with 10% FBS at 37°C under a humidified atmosphere of 5% CO_2_. Cells (3 × 10^4^/well) were seeded in a 96-well plate and incubated for 24 h. The test compounds at different concentrations were added to each well and further incubated for 24 or 48 h under the same conditions. Then, 10 μl of the MTT was added to each well at a concentration of 5 mg/ml and incubated for 4 h. The medium containing MTT was then gently replaced by DMSO and pipetted to dissolve any formazan crystals formed. Absorbance was then determined on a Tecan Sunrise microplate reader at 490 nm. The concentration required to inhibit cell growth by 50% (IC_50_) was calculated from inhibition curves.

## Results and Discussion

**Figure 1:**
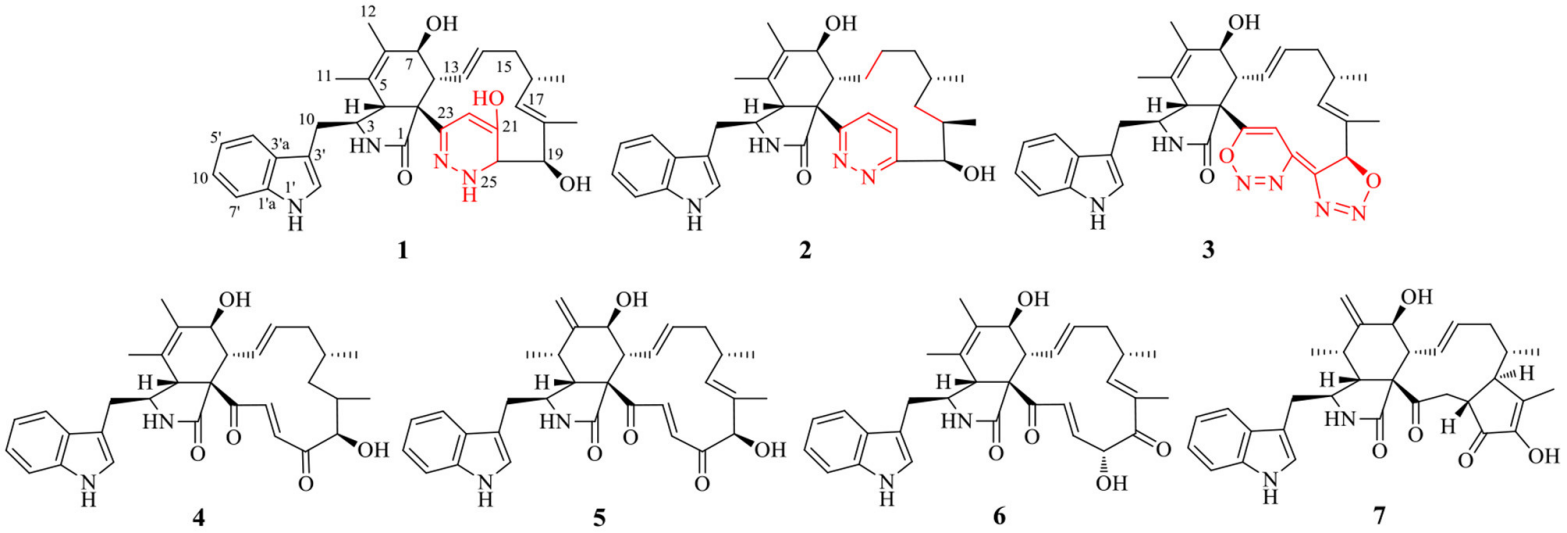
Structures of obtained compounds **1**–**7**.

The ethyl acetate extract of *C. madrasense* 375 was obtained from the submerged fermentation liquid and divided into two parts; part 1 was treated with hydrazine monohydrazine and hexahydropyridine (catalyst) to give a chemically modified production. HPLC (Supplementary Figure 35) and ^13^C NMR (Supplementary Figure 36) were performed to analyze changes in the extract. Modified production and raw extract were further separated and purified through reversed-phase C18 (ODS) column chromatography, Sephadex LH-20, as well as semipreparative HPLC to afford compounds **1**–**7** (Figure 1).

### Identification of Compounds

**Figure 2:**
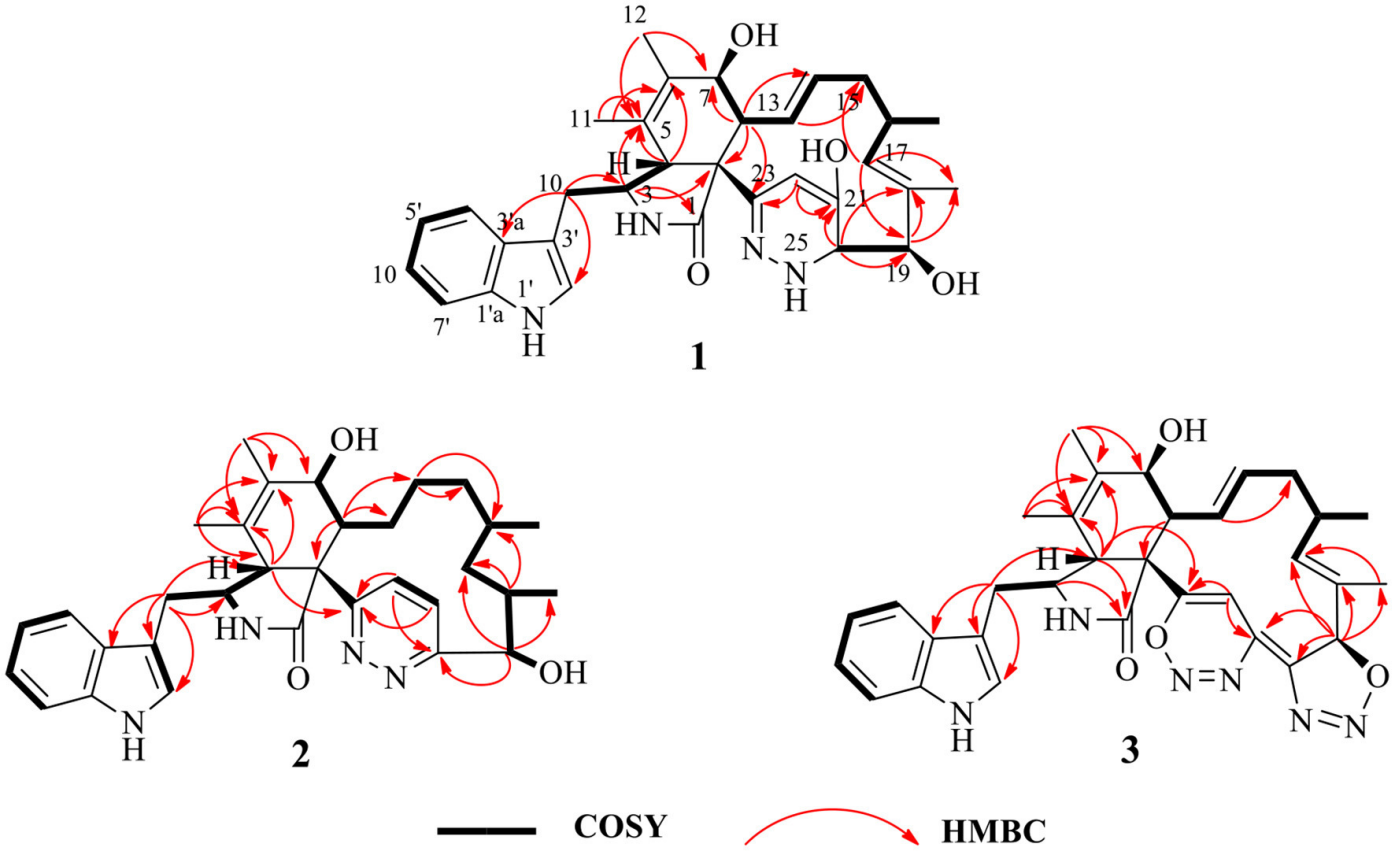
^1^H–^1^H COZY, selected HMBC correlations of compounds **1**–**3**.

Chaetoglobosin B_1_ (**1**) was obtained as a white amorphous powder. The molecular ion peak in the HR-ESI-MS (*m/z* 543.2950 [M+H]^+^ (Supplementary Figure 7), calcd for 543.2966, C_32_H_39_N_4_O_4_) and NMR data indicated that the molecular formula of **1** is C_32_H_38_N_4_O_4_ (16 unsaturations). Combined ^1^H and ^13^C NMR spectra (Tables 1, 2; Supplementary Figures 1, 2) and HSQC (Figure 2; Supplementary Figure 4), HMBC (Figure 2; Supplementary Figure 5) experiments indicated the presence of four methyl groups, two methylene units, seven methine units, one quaternary carbon, 16 olefinic or aromatic carbons, as well as one amide carbonyl carbon. The characteristic signals of ^1^H and ^13^C NMR (Tables 1, 2; Supplementary Figures 1, 2) suggested that compound **1** was most likely a chaetoglobosin derivative (Gao et al., 2019; Chen et al., 2020). Detailed analysis of the 1D (Supplementary Figures 1, 2) spectra of **1** revealed a close similarity with those reported chaetogobosin B (**4**) (Sekita et al., 1982, 1983), except that the C-20, C-21, C-22, and C-23 signals at δ_C_197.3, 136.0, 136.4, and 201.3 in chaetogobosin B (**4**) were replaced by δ_C_73.0, 147.0, 102.6, and 155.0 in **1**, respectively. The chemical shift values of C-20 (δ_C_ = 73.0) and C-23 (δ_C_ = 155.0) revealed the connection with the additional nitrogen or oxygen atom. Comparison of the molecular formula for chaetogobosin B (**4**) and **1**, **1** also was found to contain two additional nitrogen atoms, with the C-20, C-21, C-22, and C-23 carbons to constitute a pyridazin ring. Furthermore, the HMBC correlations (Figure 2; Supplementary Figure 5) from H-20 to C-18, C-19 and C-21, H-22 to C-21 and C-23, and from H-8 to C-23, as well as ^1^H-^1^H COZY correlations (Figure 2; Supplementary Figure 3) of H-20/H-19 supported this deduction. In the NOESY spectrum (Supplementary Figure 6), the relative configurations of H-4 and H-8 were assigned as β-orientations based on cross-peaks of H-4/H-8/H-14, and H-7/H-13 implied that H-7 was α-orientation. Furthermore, correlations from H-13 and H-17 to 15α (δ_H_ 1.73) and from H-17 to H-19 and H-16-Me suggested that 16-Me and H-19 were cofacial and α-oriented. Correlations between H-16 and H-18-Me and large coupling constants (*J* = 14.2 Hz) confirmed the double bonds (Δ13 and Δ17) to be *E*-geometry, and the relative configuration of **1** was identical to that of reported chaetoglobosin B. Therefore, the structure of compound **1** was unambiguously assigned by a detailed analysis of the 1D and 2D NMR spectra (Figure 2; Supplementary Figures 1–6).

Chaetoglobosin B_2_ (**2**) is a white amorphous powder with the molecular formula C_32_H_40_N_4_O_3_ (15 unsaturations) as deduced from an ion at *m/z* 529.3184 [M+H]^+^ in the HR-ESI-MS (Supplementary Figure 17). The comparison of the ^1^H and ^13^C NMR data (Tables 1, 2) between **1** and **2** showed that both possess a similar chaetogobosin skeleton, with the difference as follows: the four olefinic carbons at C-13 (129.9), C-14 (135.1), C-17 (137.8), and C-18 (133.5) in **1** were replaced by alkyl carbons (C-13, 25.2; C-14, 27.7; C-1730.2; C-18, 34.4) and one amino carbon (73.0, C-20) instead of by an imine carbon (165.5, C-20); the hydroxy group at C-21 disappeared, supported by the proton and carbon resonances at δ_H_ 7.76 (br d, 8.7, H-22) and C-21 (125.1) in **2**. Furthermore, the observed ^1^H-^1^H COSY correlation (Figure 2; Supplementary Figure 13) of H-8/H_2_-13/H_2_-14/H_2_-15/H-16/H_2_-17/H-18/H-19 as well as HMBC correlations (Figure 2; Supplementary Figure 15) from H-19 to C-17, C-18-Me, and C-20, from H-21 to C-22 and C-23, from H-22 to C-20 and C-21, and from H-4 to C-23 confirmed the above deduction. The configuration of **2** at H-3, H-4, H-7, H-8, and H-16 was defined to be identical to **1** and known chaetoglobosin B based on the comparison of the NMR data. The *Z*-geometry of the Δ_21_-double bond was deduced by the coupling constant (*J* = 8.7) between H-21and H-22, and the NOESY correlations (Supplementary Figure 16) of H-16/Me-18 suggested H-18 should be β-oriented. Therefore, considering the molecular formula and NMR data of **2**, a pyridazine ring fused into the chaetogobosin scaffold formed the structure of **2**.

**Figure 3:**
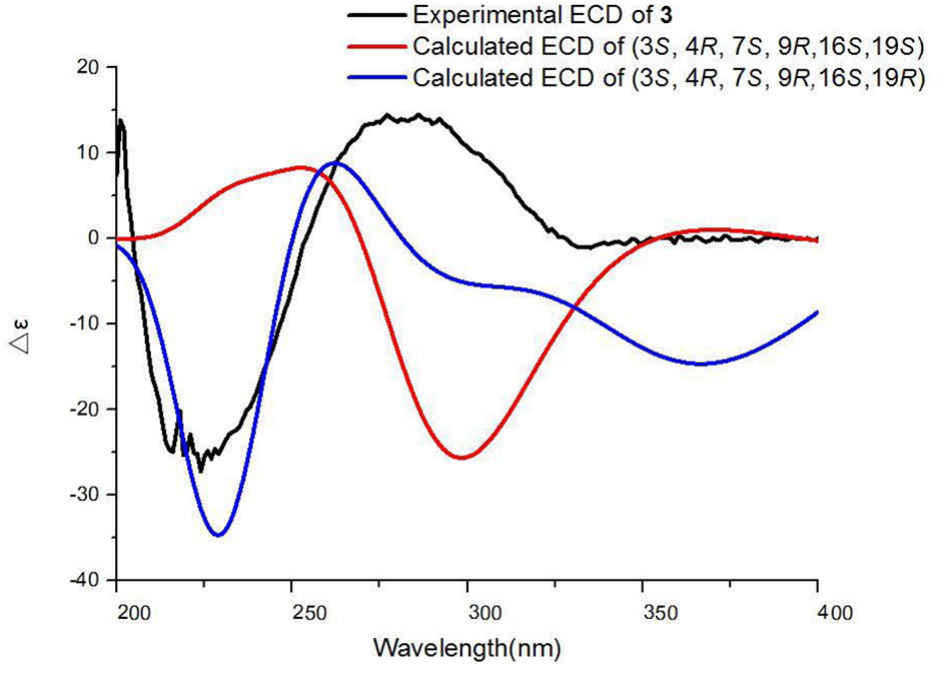
Experimental and calculated ECD spectra of compound **3**.

Chaetoglobosin B_3_ (**3**) was obtained as a white amorphous powder. Its molecular formula was assigned as C_32_H_34_N_6_O_4_ (19 unsaturations) by HR-ESI-MS (Supplementary Figure 26), which showed a protonated molecule peak at *m/z* 567.2738 [M+H]^+^. The ^1^H and ^13^C NMR data (Tables 1, 2; Supplementary Figures 20, 21) suggested compounds **3** and **1** possessed the same core structure except that the heterocyclic incorporated to the 13-membered macrocyclic ring moiety. Comparing the molecular formula of **3** and **1** suggested the presence of two more nitrogen atoms and four less hydrogen atoms, indicating that two heterocyclic moieties with nitrogen atoms possibly incorporated into chaetoglobosins core structure. However, on the basis of NMR data (Tables 1, 2; Supplementary Figures 20–25) and molecular formula, there may be four possible structures (Supplementary Figure 37) of compound **3**. Considering chemical synthesis, the structure of **3a** accords with the reaction production of chaetoglobosin B and hydrazine monohydrate. To further verify the accurate configuration of C-19 of **3**, the ECD experiments and ECD simulation (Figure 3) were both performed. The results corroborated that the configuration of C-19 was established as 19*R*. Thus, compound **3** was named chaetoglobosin B_3_ as shown in Figure 1.

The known cytochalasan alkaloid, chaetoglobosin B (**4**), chaetoglobosin D (**5**), chaetoglobosin E (**6**), and cytoglobosin A (**7**) were identified by comparing their 1D NMR and ESI-MS data with those in the scientific literature. To make sure that **3** is not a scalemic or racemic mixture, an HPLC analysis was carried out on a Agilent 1200 (Agilent Technologies, USA) system with a chiral column Daicel Chiralcel OD-H column (Supplementary Figure 38). The result indicated that **3** is a monomeric compound.

### The Plausible Synthesis Pathway

**Scheme 1:**
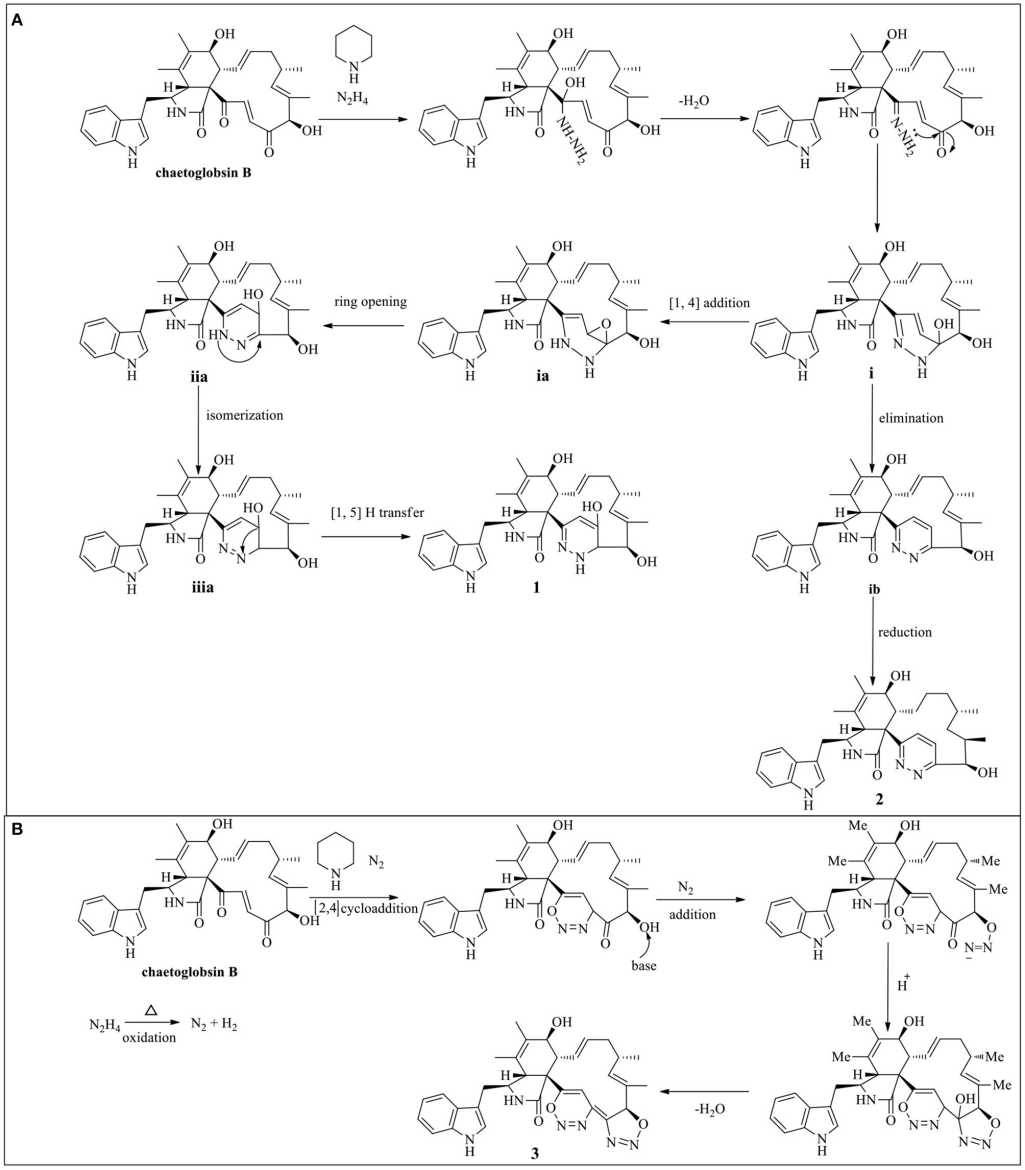
Plausible synthesis pathway of **1**–**2 (A)** and **3 (B)**.

The putative synthesis pathway for compounds **1**–**3** was proposed starting from chaetoglobosin B through hydrazinolysis reaction. The precursor chaetoglobosin B underwent an intermolecular nuclephilic addition-elimination reaction with hydrazine, catalyzed by hexahydropyridine to give the intermediates i, followed by [1, 4] addition, ring opening, isomerization, and [1, 5] proton transfer reactions led to the formation of **1** (Scheme 1A). **2** was formed by an elimination and hydrogenation reduction of intermediates i (Scheme 1A). **3** was an unexpected production of cytochalasan and hydrazine monohydrate. There was little literature on the formation of **3** and its analogs. Herein, we surmised that **3** may be not directly produced by cytochalasan and hydrazine monohydrate. Therefore, we propound that **3** may be formed by cycloaddition of cytochalasan and the oxidation of hydrazine monohydrate. The plausible pathway of **3** is proposed as shown in Scheme 1B.

### Biological Activity

Compounds **1**–**7** were evaluated for their inhibitory effects toward human non-small-cell lung carcinoma (A549, HCC827), human colon cancer (SW620), and human breast cancer (MDA-MB-231) cell lines. As shown in Table 3, compounds **1** and **7** exhibited selected antiproliferative activity on non-small-cell lung carcinoma A549 and human colon cancer SW620 cells, respectively. Compound **4** showed selected antiproliferative activity on three cancer cell lines, HCC827, SW620, and MDA-MB-231with IC_50_ values of 11.6, 8.8, and 11.4 μM, respectively. Compound **5** displayed moderate antiproliferative activity on four cancer cell lines with the IC_50_ values ranging from 6.9 to 10.6 μM. It is noteworthy that compound **6** possessed potent activity (IC_50_ values ranging from 1.7 to 9.9 μM), which were all stronger than the positive control cisplatin. In addition, compound **6** showed a more significant inhibitory effect on two drug-resistant HCC827 cells (Gefitinib-resistant, Osimertinib-resistant) with IC_50_ values of 5.1 and 2.8 μM, respectively. However, compounds **2**–**3** showed no obvious inhibitory effect (IC_50_ > 20 μM). According to the result of the cytotoxicity assay (Tables 3, 4), it is worth noting that the heterocyclic ring fused into the macrocyclic of compounds **1–3** may be reduced the activity by blocking the interaction with its site of action.

**Table 3:** Cytotoxicity of compounds **1**–**7** (IC_50_ μM).

**Compounds**	**A549**	**HCC827**	**SW620**	**MDA-MB-231**
1	11.0	>20	>20	>20
2	>20	>20	>20	>20
3	>20	>20	>20	>20
4	>20	11.6	8.7	11.4
5	6.9	6.9	7.7	10.6
6	2.0	1.7	2.9	9.9
7	>20	>20	13.9	>20
cis-platin^a^	6.4	4.5	4.2	47.7

a *Used as a positive control*.

**Table 4:** Cytotoxicity of compound **6** against two drug-resistant HCC827 cells (Gefitinib-resistant, Osimertinib-resistant) (IC_50_ μM).

**Compounds**	**HCC827 (Gefitinib-resistant strain)**	**HCC827 (Osimertinib-resistant strain)**
6	5.1	2.8
Gefitinib^a^	65.1	–^b^
Osimertinib^a^	–^b^	11.9

a *Used as positive controls*.

b *Not tested*.

## Conclusion

As a fungus, the *Chaetomium* genus contains more than 100 species and plenty of structural novel metabolites (including cytochalasans) with a wide range of biological activities represented from the species (Li et al., 2018). To broaden the chemical diversity of cytochalasans with interesting activity, three novel cytochalasan derivatives, termed chaetoglobosins B_1_-B_3_ (**1**–**3**) were isolated from the chemical modification of an extract of *C. madrasense* 375, and four known metabolite chaetoglobosins (**4**–**7**) were also identified. Compounds **1**–**3** represented the first examples of hydrazine-containing heterocycle fused into the macrocyclic of cytochalasans. The structures of **1**–**3** were elucidated by means of spectroscopic data, quantum-chemical ECD simulation. In the antiproliferative assay, compound **6** displayed potent inhibition activity against four human cancer cell lines (A549, SW620, MDA-MB-231, and HCC827), as well as two drug-resistant (gefitinib, osimertinib) HCC827cell lines; it is the first time to evaluate the drug-resistant activity of cytochalasans. However, the inhibition activity of new compounds **1**–**3** on three cancer cell lines (SW620, MDA-MB-231, and HCC827) was weaker than unreacted chaetoglobosin B (**4**). Only compound **1** exhibited selected antiproliferative activity on non-small-cell lung carcinoma A549 cells. The results indicated the active sites of **1**–**3** maybe at C-19–C-23 which were blocked by hydrazine-containing heterocycle. Thus, chemical modification of extract of *C. madrasense* 375 works not only by considering the modification of the active group but also increasing the site of action in future research. This study provides useful insights that natural products could be an alternative source to generate biological molecules with a novel structure for drug screening through chemical diversification.

## Data Availability Statement

The datasets presented in this study can be found in online repositories. The names of the repository/repositories and accession number(s) can be found in the article/Supplementary Material.

## Author Contributions

QG and LC conceived ideas, designed experiments, and wrote the original draft. QG, ZY, and JZ performed the experiments. JC contributed to cytotoxicity testing. YR and BY contributed to the chemical diversification and plausible synthesis pathway. WY, GD, and WZ analyzed the data and critically reviewed the manuscript. XW and GD provided the fungal material. All authors listed have approved the work for publication.

## Conflict of Interest

The authors declare that the research was conducted in the absence of any commercial or financial relationships that could be construed as a potential conflict of interest.
