# Supplementary material for: Hydrazine-Containing Heterocycle Cytochalasan Derivatives From Hydrazinolysis of Extracts of a Desert Soil-Derived Fungus Chaetomium madrasense 375
Source: Front Chem. 2021 Apr 21;9:620589. doi: 10.3389/fchem.2021.620589 (PMC8097171; doi:10.3389/fchem.2021.620589)
Supplement: Supplementary file 1 [file Data_Sheet_1.docx]

**Electronic Supplementary Information (ESI)**

**Hydrazine–containing heterocycle cytochalasans derivatives from hydrazinolysis of extracts of a desert soil-derived fungus *Chaetomium madrasense* 375**

Qingfeng Guo ^a^, Jinhua Chen ^b^, Yuwei Ren ^a^, Zhenhua Yin ^a^, Juanjuan Zhang ^a^, Baocheng Yang ^a^, Xuewei Wang ^c^, Wen-Bing Yin ^c^, Wanchun Zhang ^d,^ *, Gang Ding ^e,^*and Lin Chen ^a,^*

^a^ *Zhengzhou Key Laboratory of Synthetic Biology of Natural Products, Zhengzhou Key Laboratory of Medicinal Resources Research, Huanghe Science and Technology College, Zhengzhou, 450006, China;*

^b^ *Department of Pharmacy, Affiliated Cancer Hospital of Zhengzhou University, Henan Cancer Hospital, Zhengzhou 450008, China*

^c^ *State Key Laboratory of Mycology, Institute of Microbiology, Chinese Academy of Sciences, Beijing, 100101, China*

^d^ *Henan Key Laboratory of Children’s Genetics and Metabolic Diseases, Children's Hospital Affiliated to Zhengzhou University, Zhengzhou 450018, China*

^e^ *Institute of Medicinal Plant Development, Chinese Academy of Medical Science and Union Medical College, Beijing 100193, China*

Corresponding Author:

E-mail: Lin Chen: lchenchina@163.com; Gang Ding: gding@implad.ac.cn; Wanchun Zhang: zhangwancun@126.com

**Table of contents**

**Figure S1.** ^1^H NMR spectrum of compound **1** in CD_3_OD (400 MHz)

**Figure S2.** ^13^C NMR spectrum of compound **1** in CD_3_OD (100 MHz)

**Figure S3.** COSY spectrum of compound **1** in CD_3_OD (400 MHz)

**Figure S4.** HSQC spectrum of compound **1** in CD_3_OD (400 MHz)

**Figure S5.** HMBC spectrum of compound **1** in CD_3_OD (400 MHz)

**Figure S6.** NOESY spectrum of compound **1** in CD_3_OD (400 MHz)

**Figure S7.** HRESIMS spectrum of compound **1**

**Figure S8.** EIMS spectrum of Compound **1**

**Figure S9.** UV spectrum of Compound **1**

**Figure S10.** ^1^H NMR spectrum of compound **2** in DMSO-*d*_6_ (400 MHz)

**Figure S11.** ^13^C NMR spectrum of compound **2** in DMSO-*d*_6_ (100 MHz)

**Figure S12.** DEPT spectrum of compound **2** in DMSO-*d*_6_ (100 MHz)

**Figure S13.** COSY spectrum of compound **2** in DMSO-*d*_6_ (400 MHz)

**Figure S14.** HMQC spectrum of compound **2** in DMSO-*d*_6_ (400 MHz)

**Figure S15.** HMBC spectrum of compound **2** in DMSO-*d*_6_ (400 MHz)

**Figure S16.** NOESY spectrum of compound **2** in DMSO-*d*_6_ (400 MHz)

**Figure S17.** HRESIMS spectrum of compound **2**

**Figure S18.** EIMS spectrum of Compound **2**

**Figure S19.** UV spectrum of Compound **2**

**Figure S20.** ^1^H NMR spectrum of compound **3** in DMSO-*d*_6_ with one drop CD_3_OD (400 MHz)

**Figure S21.** ^13^C NMR spectrum of compound **3** in DMSO-*d*_6_ with one drop CD_3_OD (100 MHz)

**Figure S22.** COSY spectrum of compound **3** in DMSO-*d*_6_ with one drop CD_3_OD (400 MHz)

**Figure S23.** HSQC spectrum of compound **3** in DMSO-*d*_6_ with one drop CD_3_OD (400 MHz)

**Figure S24.** HMBC spectrum of compound **3** in DMSO-*d*_6_ with one drop CD_3_OD (400 MHz)

**Figure S25.** DEPT spectrum of compound **3** in DMSO-*d*_6_ with one drop CD_3_OD (400 MHz)

**Figure S26.** HRESIMS spectrum of compound **3**

**Figure S27.** EIMS spectrum of Compound **3**

**Figure S28.** UV spectrum of Compound **3**

**Figure S29.** Experimental ECD spectra of **3** and calculated ECD spectra for (3*S*, 4*R*, 7*S*, 9*R*, 16*S*, 19*S*)-**3** and (3*S*, 4*R*, 7*S*, 9*R*, 16*S*, 19*R*)-**3**

**Table S1**. Gibbs free energies^a^ and equilibrium populations^b^ of low-energy conformers of (3*S*, 4*R*, 7S, 9*R*, 16*S*, 19*S*)-**3**

**Figure S30.** Structures and populations of the low-energy conformers of (3*S*, 4*R*, 7*S*, 9*R*, 16*S*, 19*S*)-**3**

**Table S2.** Cartesian coordinates for the low-energy reoptimized MMFF conformers of (3*S*, 4*R*, 7*S*, 9*R*, 16*S*, 19*S*)-**3** at B3LYP/6-31G(d,p) level of theory in MeOH

**Table S3.** Gibbs free energies*^a^* and equilibrium populations*^b^* of low-energy conformers of (3*S*, 4*R*, 7*S*, 9*R*, 16*S*, 19*R*)-**3**

**Figure S31.** Structures and populations of the low-energy conformers of (3*S*, 4*R*, 7*S*, 9*R*, 16*S*, 19*R*)-**3**

**Table S4.** Cartesian coordinates for the low-energy reoptimized MMFF conformers of (3*S*, 4*R*, 7*S*, 9*R*, 16*S*, 19*R*)-**3** at B3LYP/6-311+G (d, p) level of theory in CH_3_OH

**Figure S32.** Molecular phylogenetic analysis of beta-Tubulin gene from different species

**Figure S33.** beta-tubulin encoding gene sequence of *C. madrasense* 375

**Figure S34.** 18S rDNA gene sequence of *C. madrasense* 375

**Figure S35.** HPLC chromatogram analysis of the crude extracts (A) from 375 and modified production (B)

**Figure S36.** ^13^C NMR spectrum of modified production in DMSO-*d*_6_ (100 MHz)

**Figure S37.** Four possible structures of compound **3**

**Figure S38.** Chiral HPLC analysis of compound **3**

**Figure S39.** IC_50_ curve of compounds **1**-**7** against A549 cell lines

**Figure S40.** IC_50_ curve of compounds **1**-**7** against HCC827 cell lines

**Figure S41.** IC_50_ curve of compounds **1**-**7** against SW620 cell lines

**Figure S42.** IC_50_ curve of compounds **1**-**7** against MDA-MB-231 cell lines

**Figure S43.** IC_50_ curve of compounds **6** against drug-resistant HCC827 cells (A: Gefitinib-resistant, B: Osimertinib-resistant) cell lines


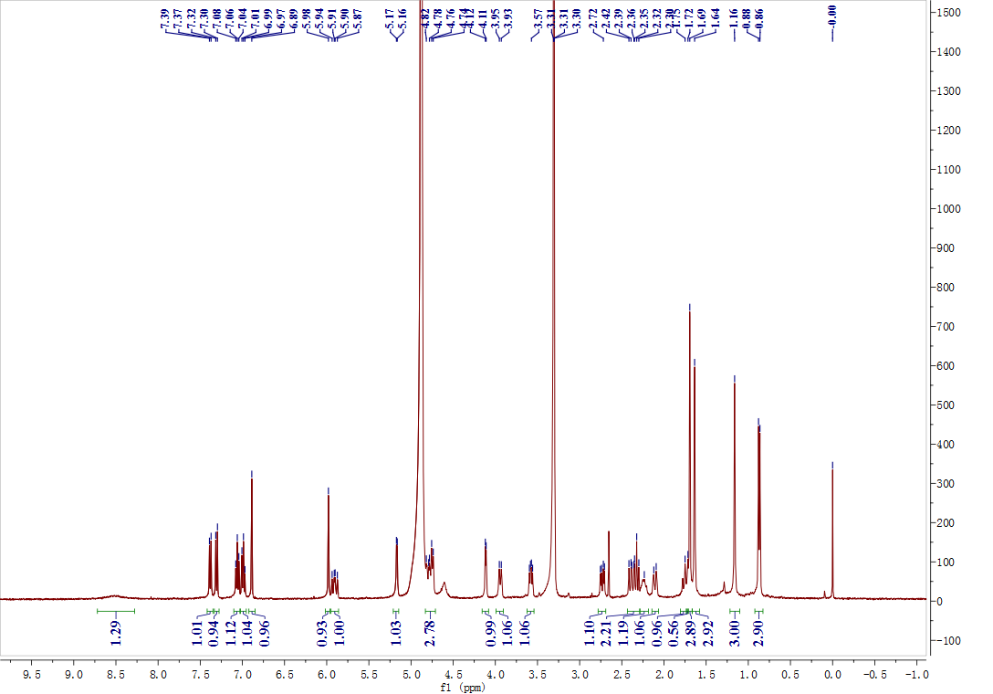


**Figure S1.** ^1^H NMR spectrum of compound **1** in CD_3_OD (400 MHz)


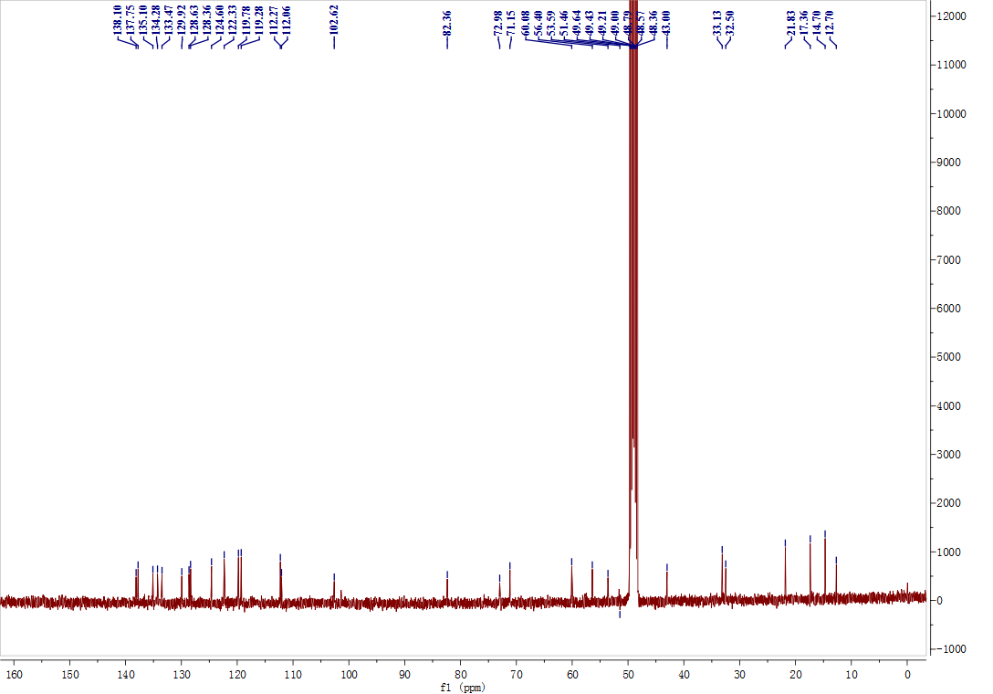


**Figure S2.** ^13^C NMR spectrum of compound **1** in CD_3_OD (100 MHz)


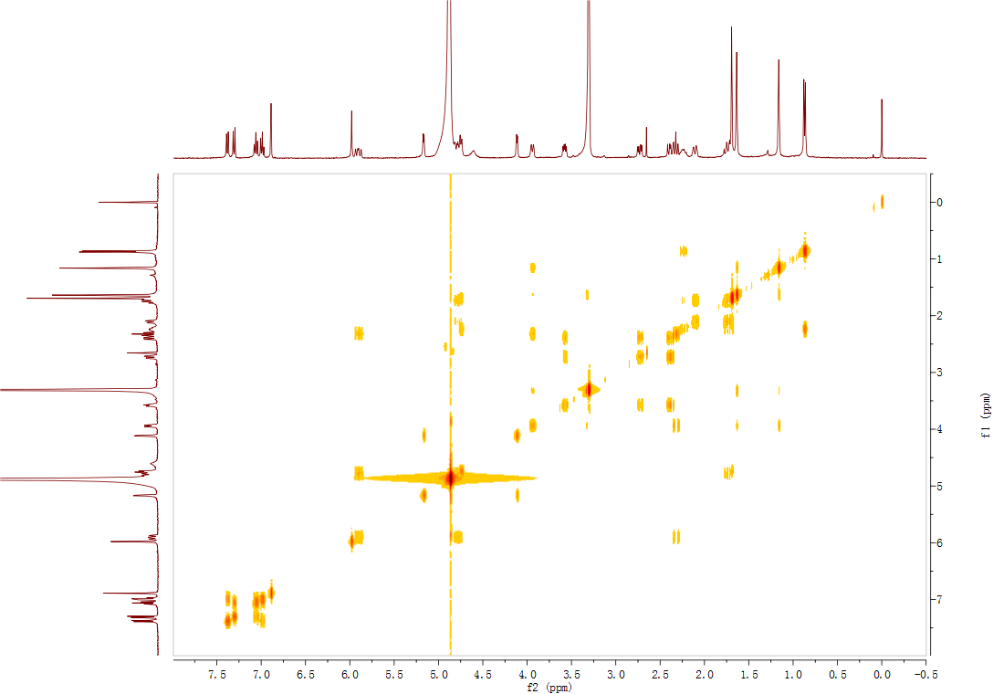


**Figure S3.** COSY spectrum of compound **1** in CD_3_OD (400 MHz)


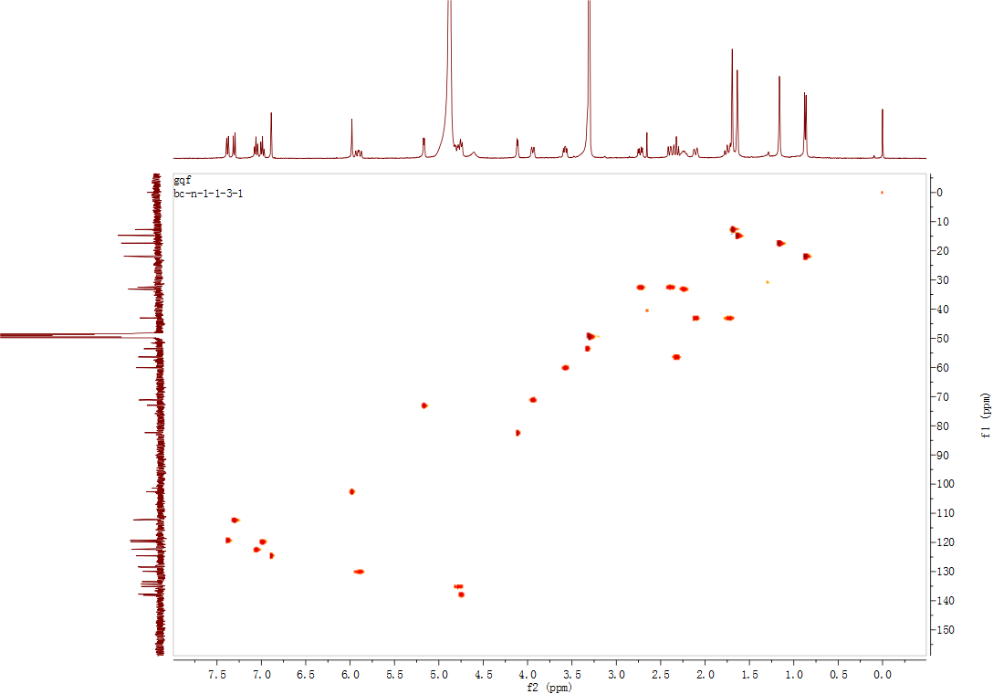


**Figure S4.** HSQC spectrum of compound **1** in CD_3_OD (400 MHz)


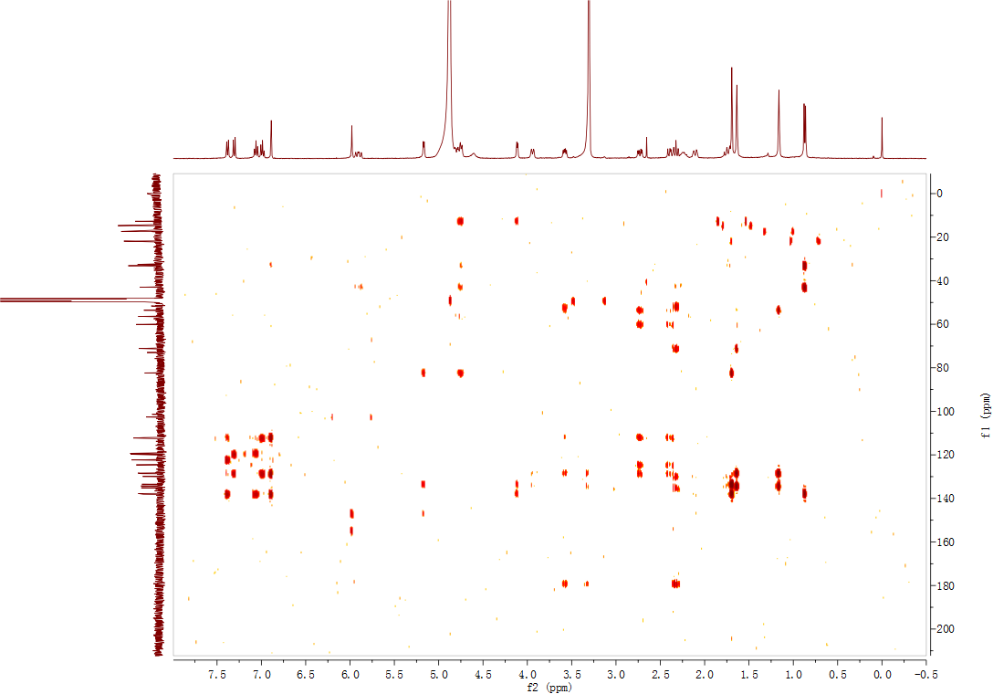


**Figure S5.** HMBC spectrum of compound **1** in CD_3_OD (400 MHz)


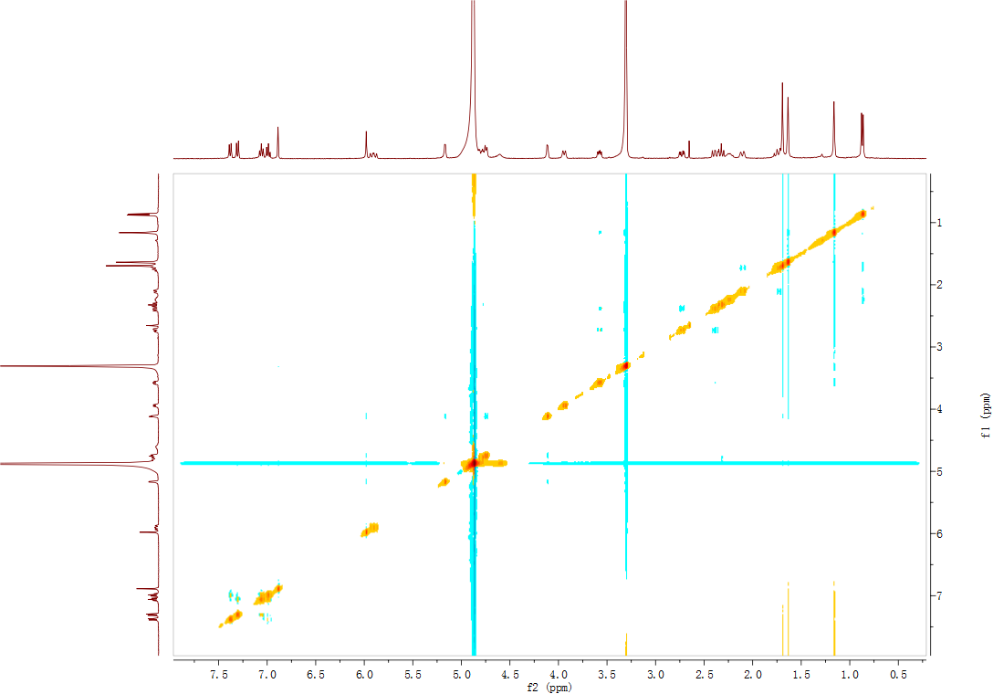


**Figure S6.** NOESY spectrum of compound **1** in CD_3_OD (400 MHz)


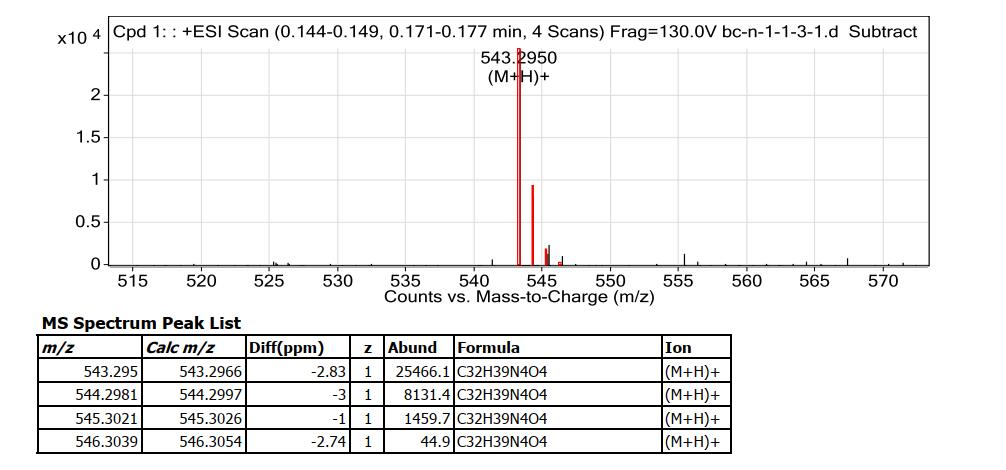


**Figure S7.** HRESIMS spectrum of compound **1**


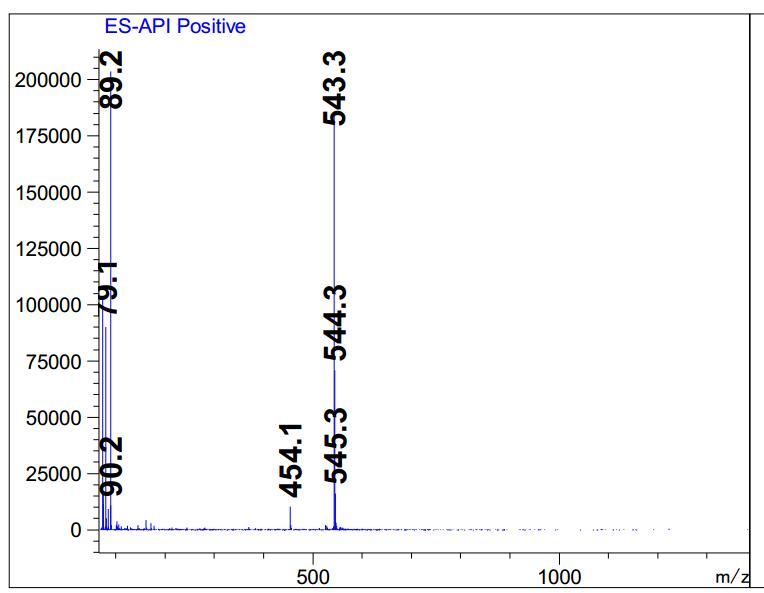


**Figure S8.** EIMS spectrum of Compound **1**


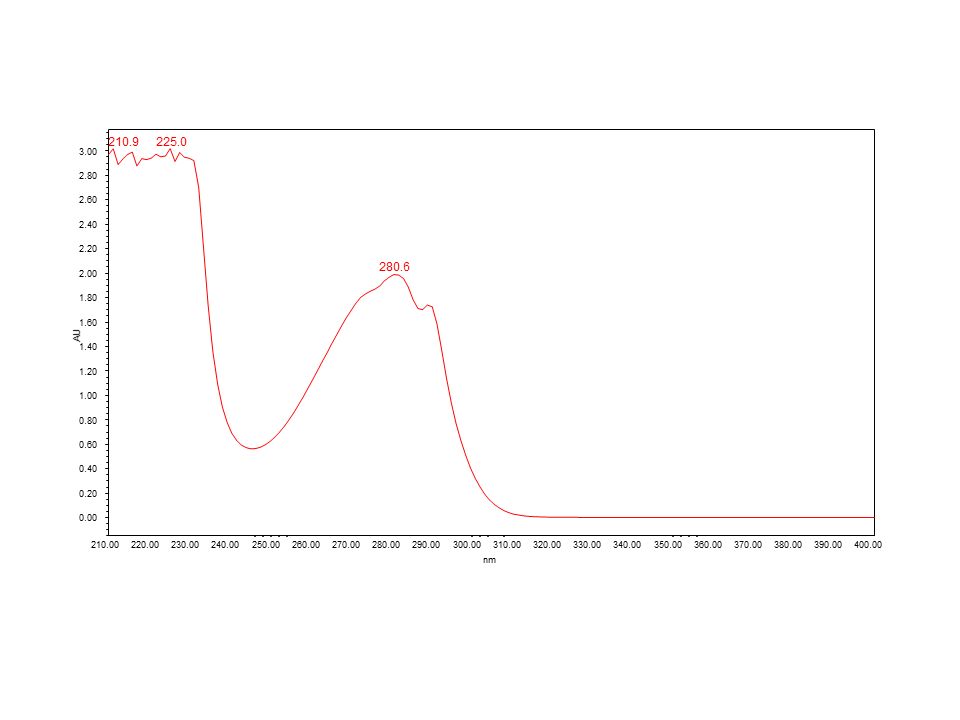


**Figure S9.** UV spectrum of Compound **1**


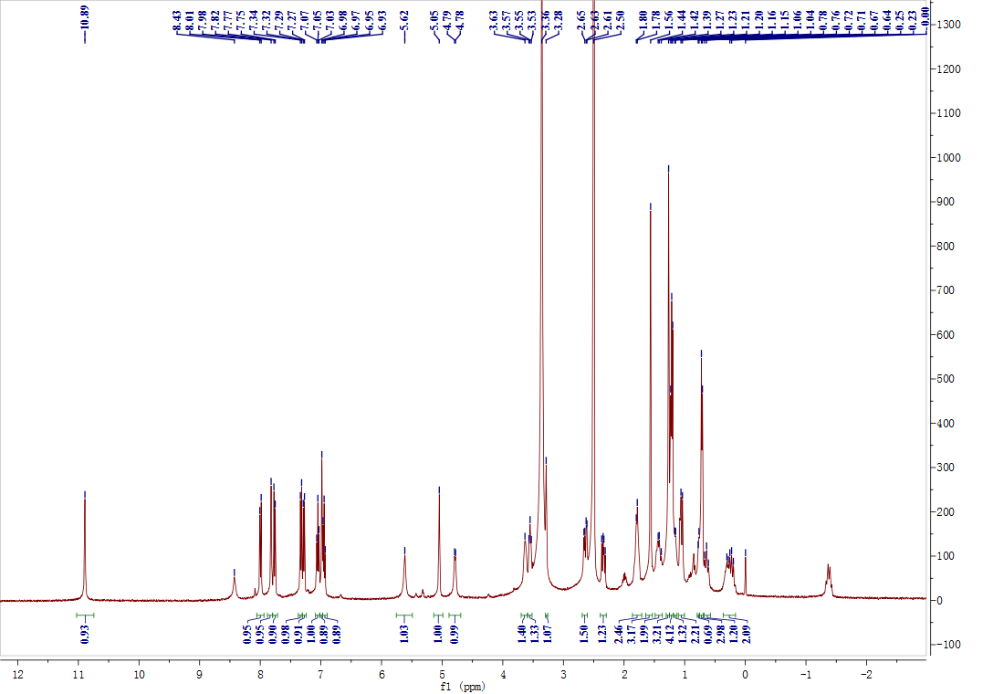


**Figure S10.** ^1^H NMR spectrum of compound **2** in DMSO-d_6_ (400 MHz)


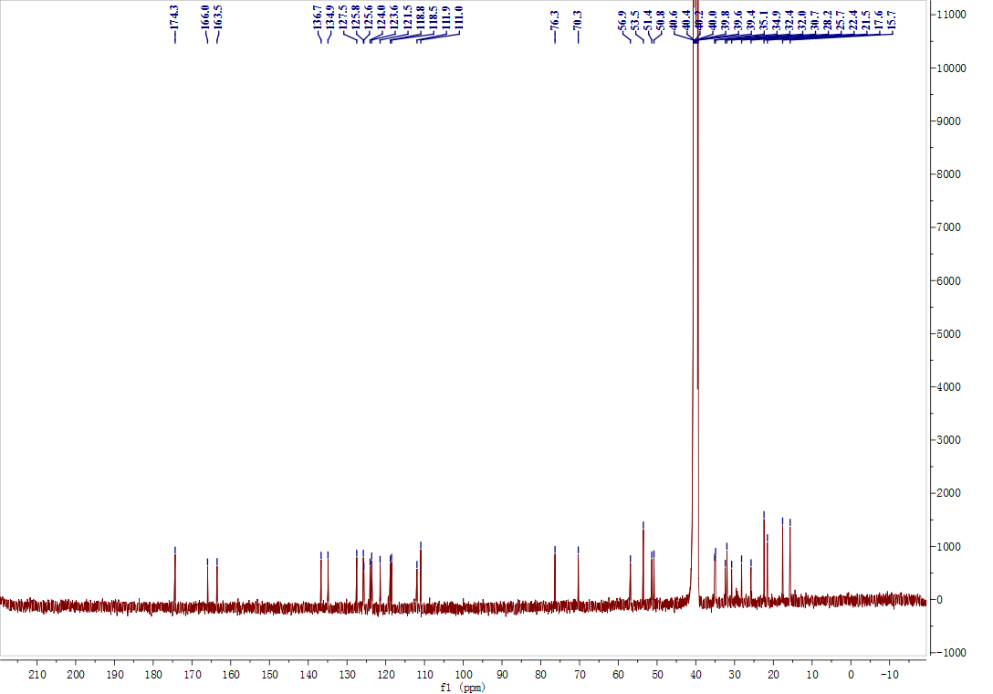


**Figure S11.** ^13^C NMR spectrum of compound **2** in DMSO-*d*_6_ (100 MHz)


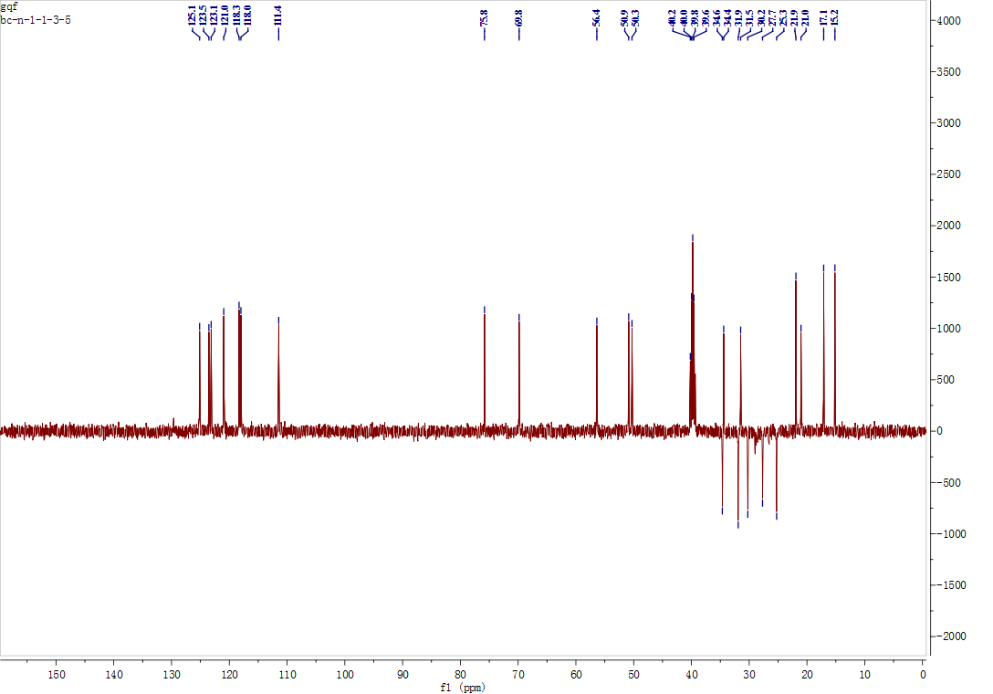


**Figure S12.** DEPT spectrum of compound **2** in DMSO-*d*_6_ (100 MHz)

**
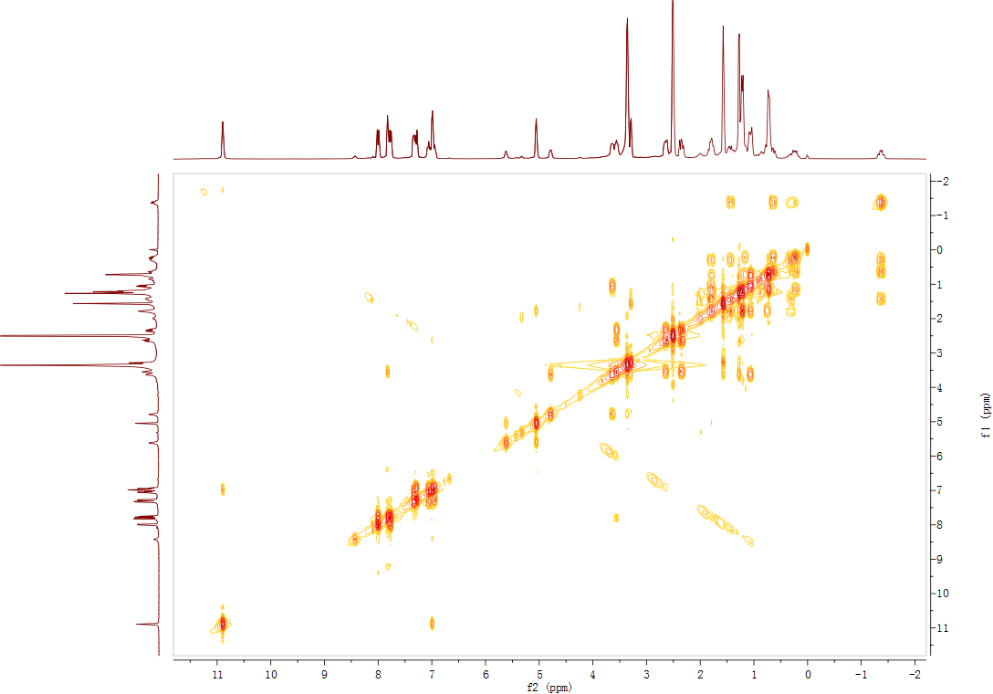
**

**Figure S13.** COSY spectrum of compound **2** in DMSO-*d*_6_ (400 MHz)

**
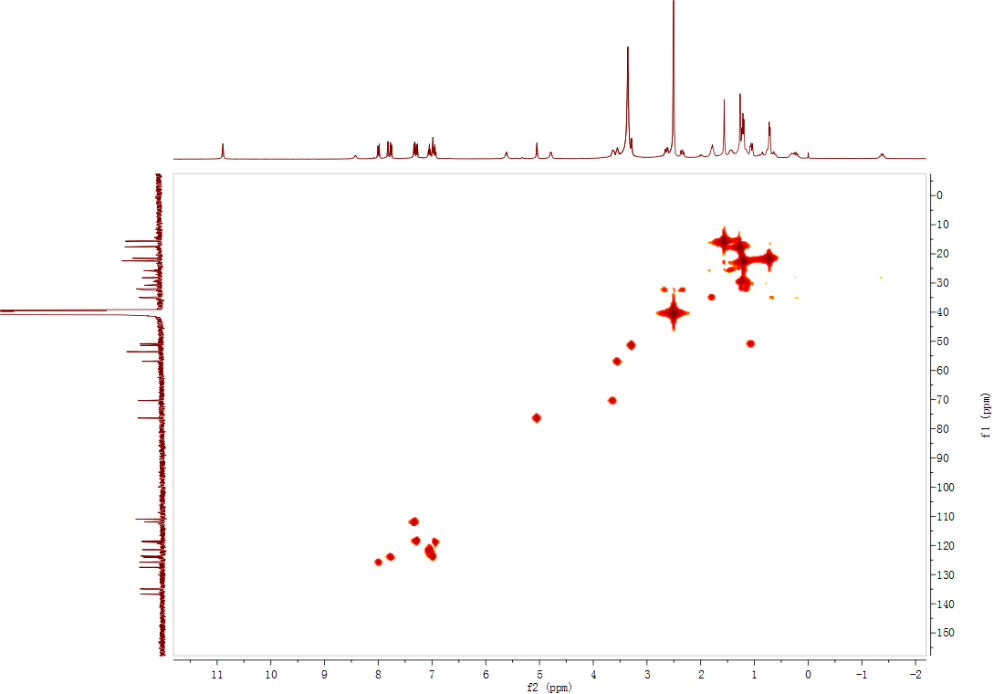
**

**Figure S14.** HMQC spectrum of compound **2** in DMSO-*d*_6_ (400 MHz)


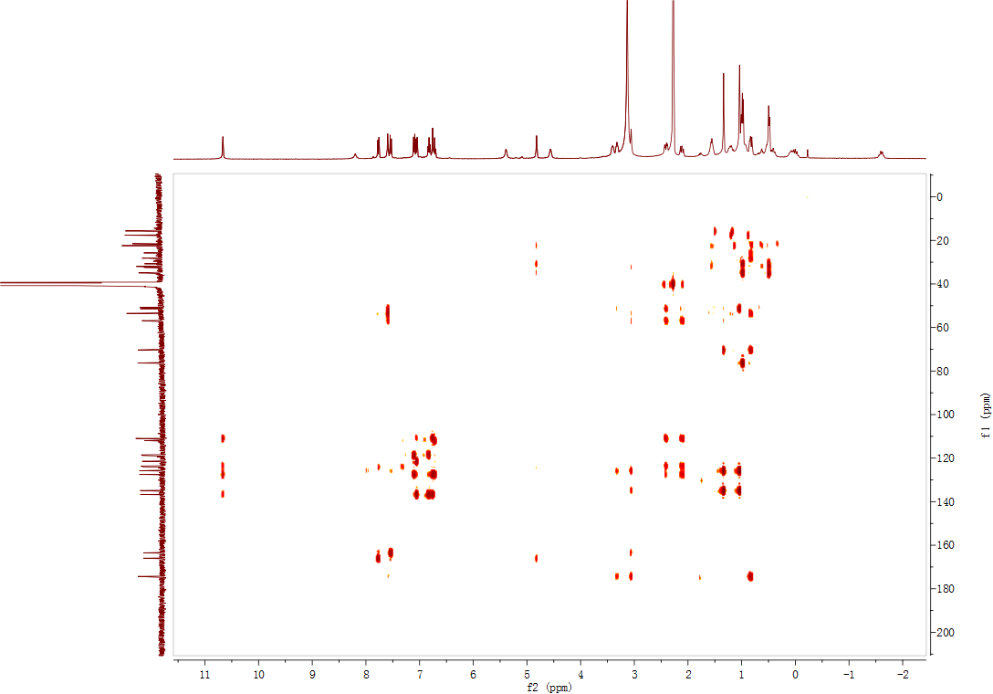


**Figure S15.** HMBC spectrum of compound **2** in DMSO-*d*_6_ (400 MHz)


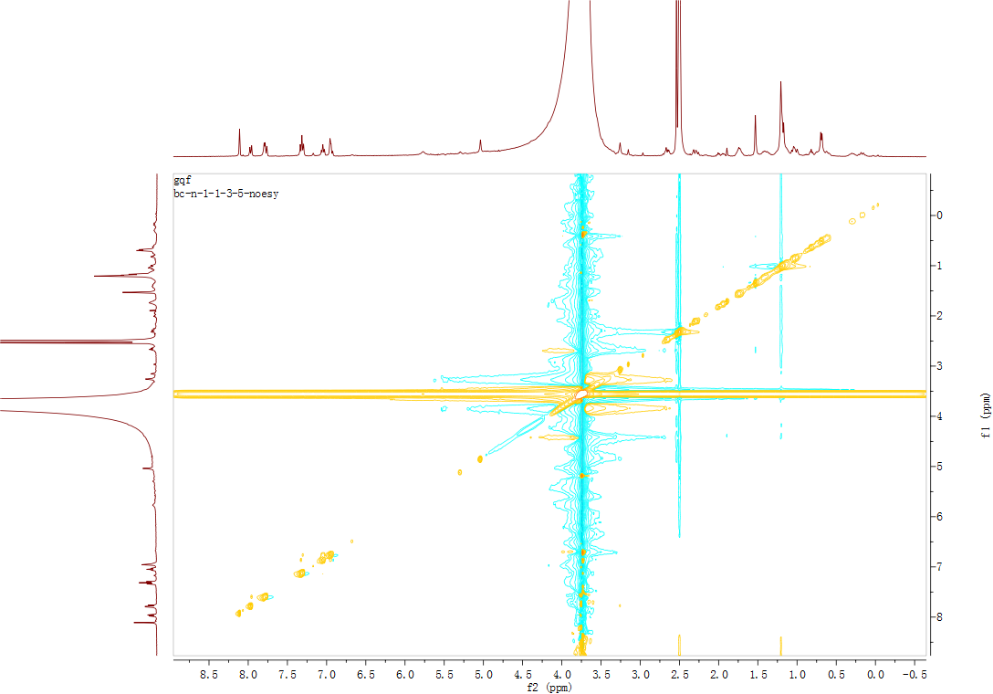


**Figure S16.** NOESY spectrum of compound **1** in DMSO-*d*_6_ (400 MHz)


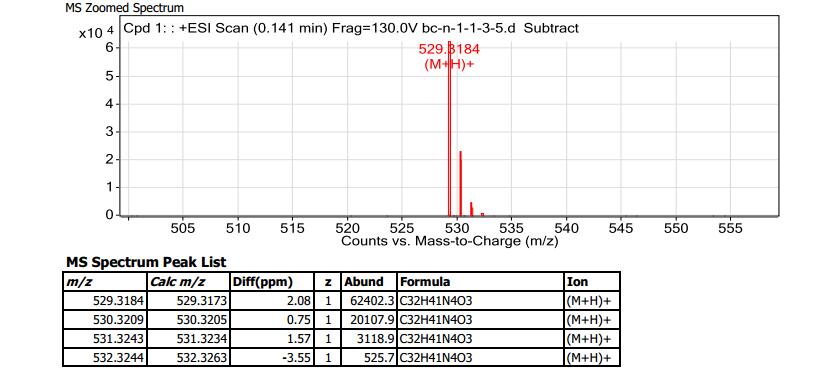


**Figure S17.** HRESIMS spectrum of compound **2**


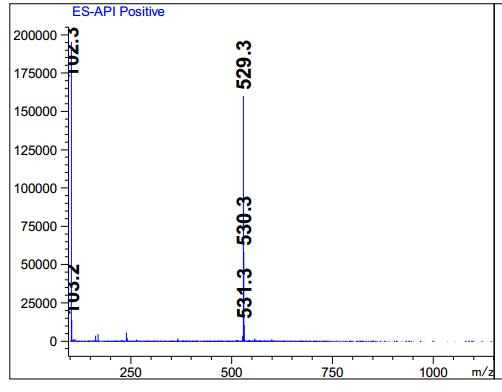


**Figure S18.** EIMS spectrum of Compound **2**


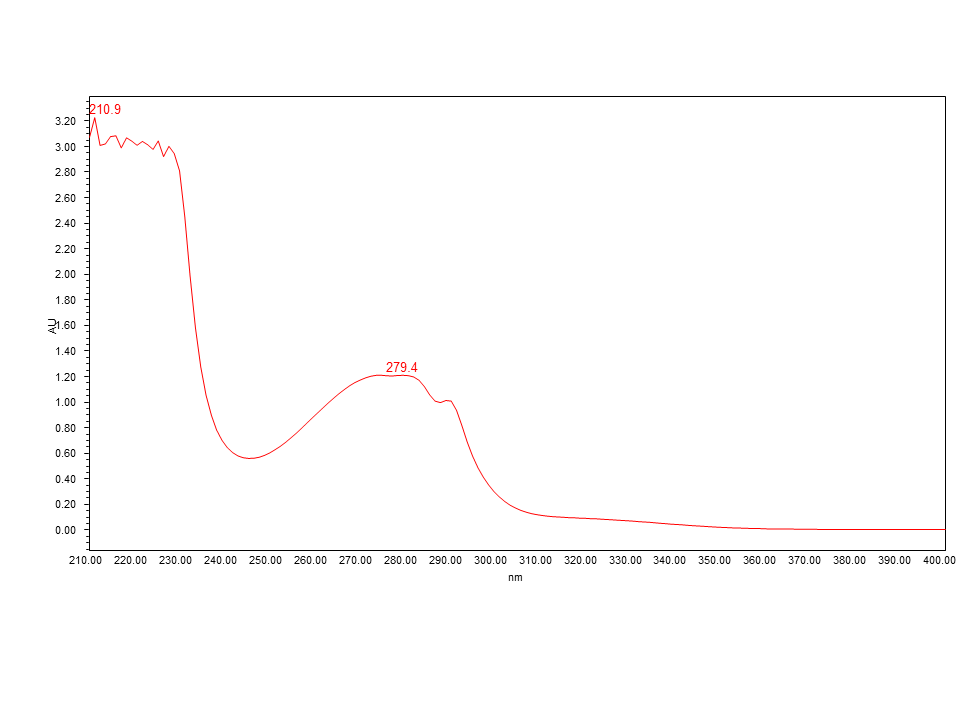


**Figure S19.** UV spectrum of Compound **2**

**
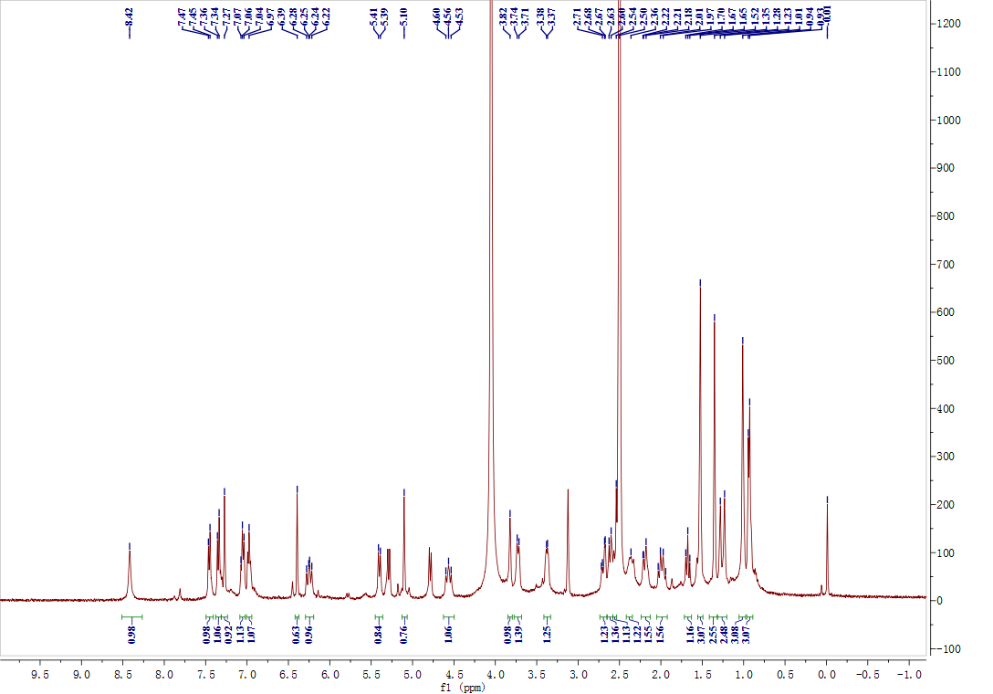
**

**Figure S20.** ^1^H NMR spectrum of compound **3** in DMSO-*d*_6_ (400 MHz)


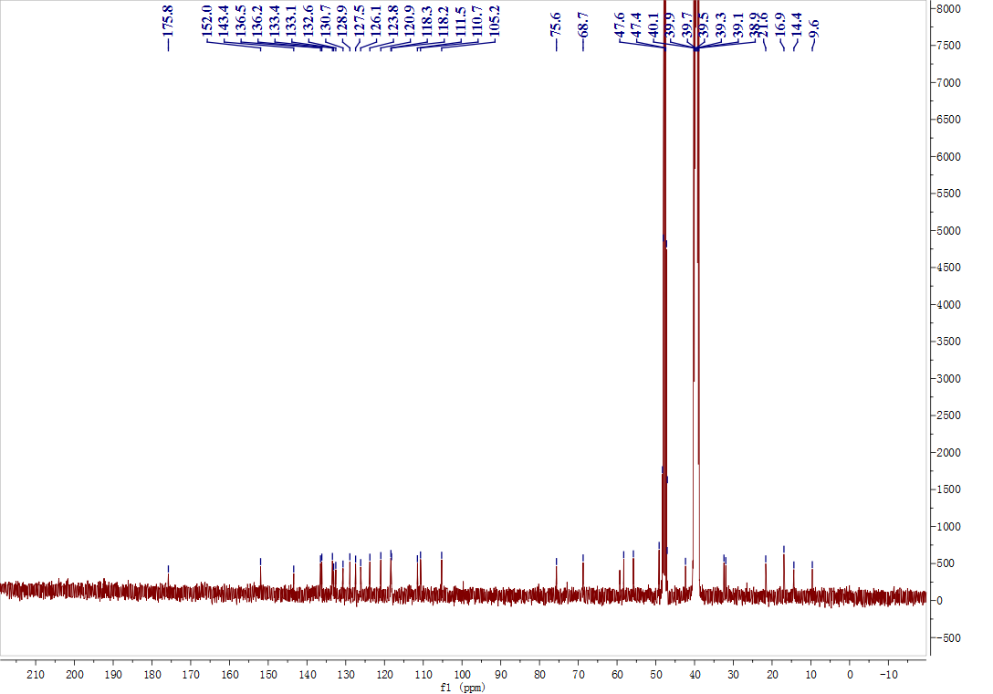


**Figure S21.** ^13^C NMR spectrum of compound **3** in DMSO-*d*_6_ (100 MHz)


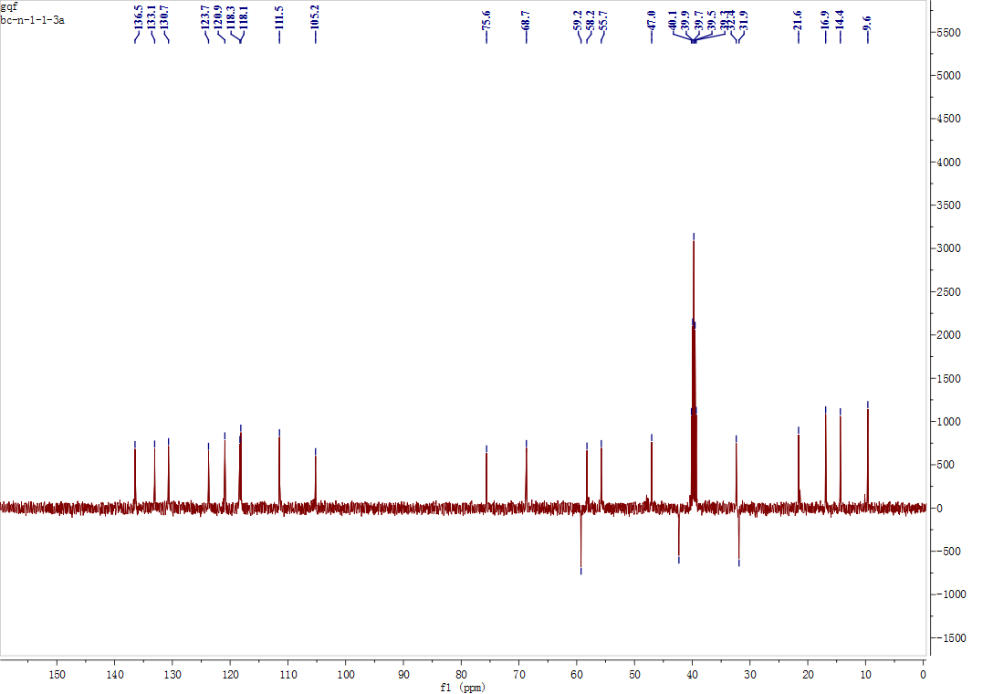


**Figure S22.** DEPT spectrum of compound **3** in DMSO-*d*_6_ (100 MHz)


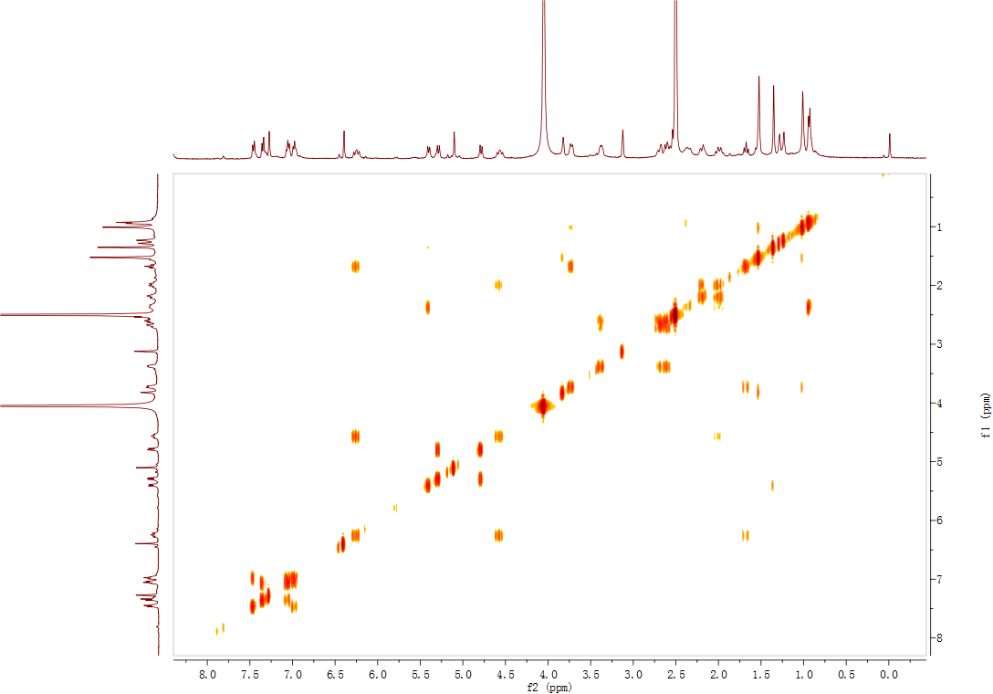


**Figure S23.** COSY spectrum of compound **3** in DMSO-*d*_6_ (400 MHz)


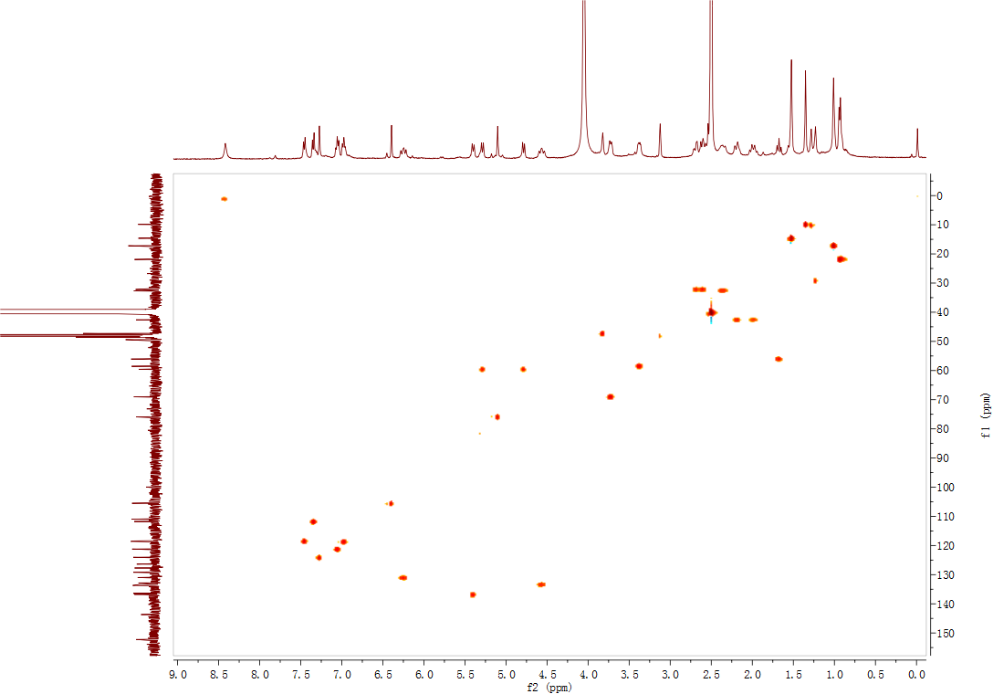


**Figure S24.** HSQC spectrum of compound **3** in DMSO-*d*_6_ (400 MHz)


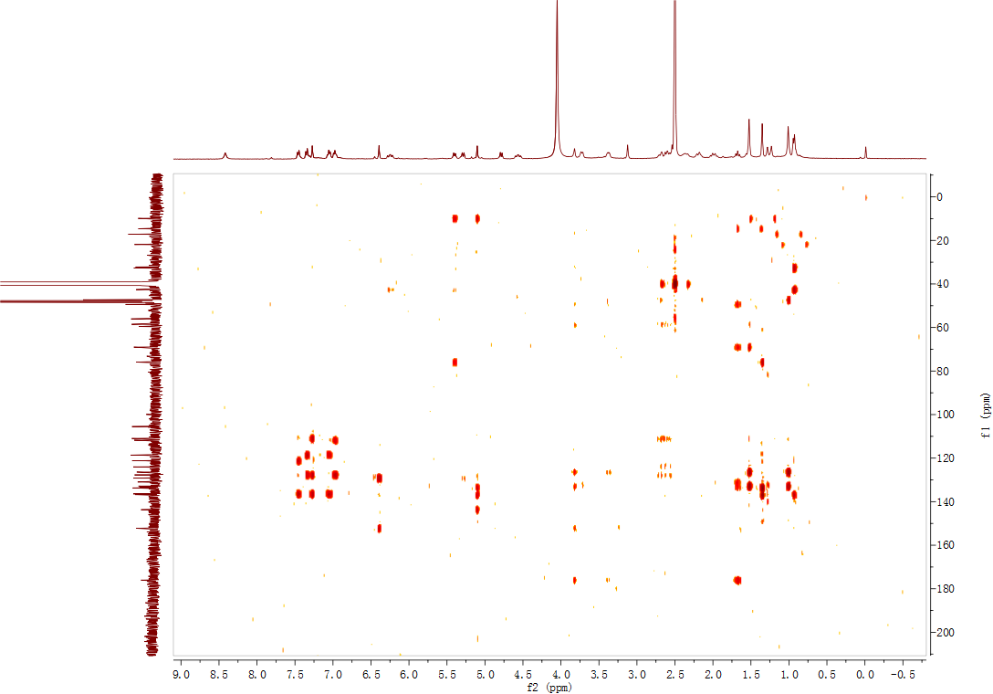


**Figure S25.** HMBC spectrum of compound **3** in DMSO-*d*_6_ (400 MHz)


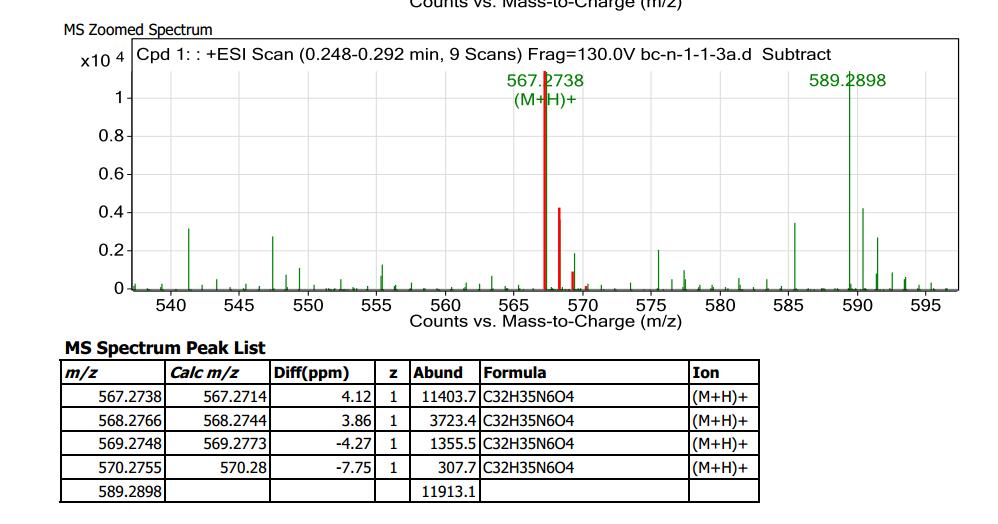


**Figure S26.** HRESIMS spectrum of compound **3**

**
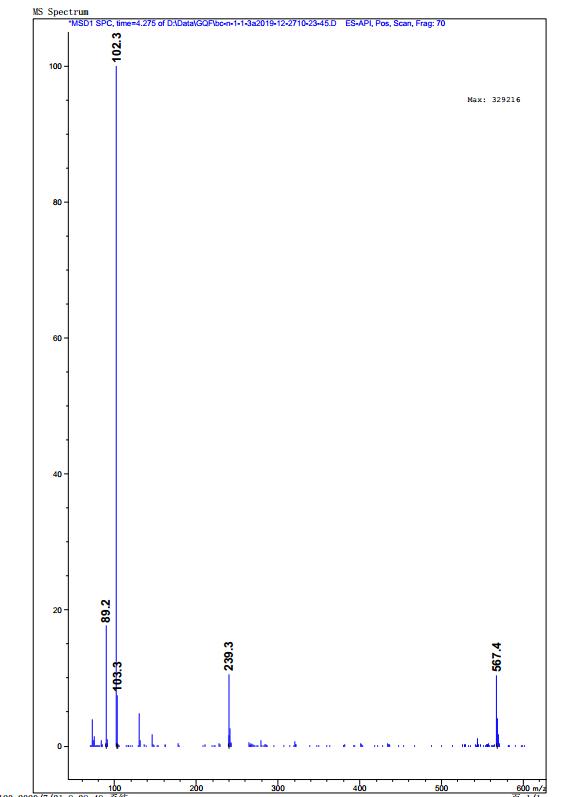
**

**Figure S27.** EIMS spectrum of Compound **3**

**
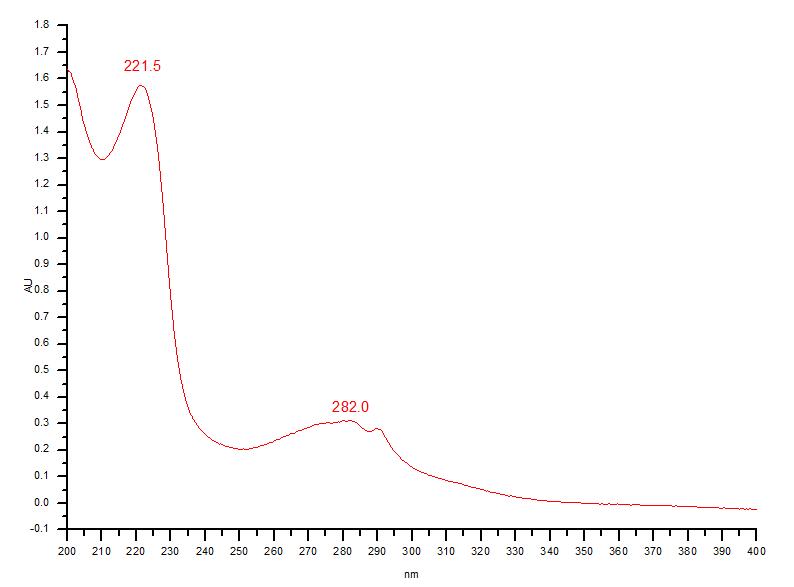
**

**Figure S28.** UV spectrum of Compound **3**


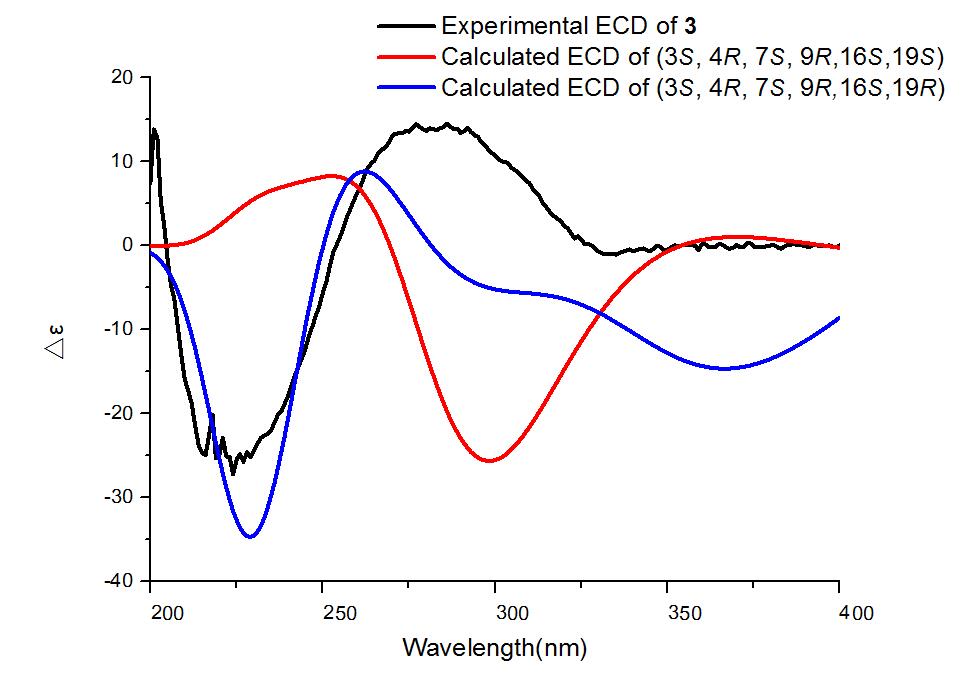


**Figure S29.** Experimental ECD spectra of **3** and calculated ECD spectra for (3*S*, 4*R*, 7*S*, 9*R*, 16*S*, 19*S*)-**3** and (3*S*, 4*R*, 7*S*, 9*R*, 16*S*, 19*R*)-**3**.

**Table S1.** Gibbs free energies*^a^* and equilibrium populations*^b^* of low-energy conformers of (3*S*, 4*R*, 7*S*, 9*R*, 16*S*, 19*S*)-**3**.

| Conformers | In MeOH | |
| --- | --- | --- |
|  | *G^a^* | *P* (%)^b^ |
| **3S-1** | -1172544.05836581 | 30.85 |
| **3S-2** | -1172544.29870214 | 46.31 |
| **3S-3** | -1172543.10706065 | 6.18 |
| **3S-4** | -1172543.63981664 | 15.21 |
| **3S-5** | -1172542.24548942 | 1.44 |
|  |  |  |
| *^a^*B3LYP/6-31G(d,p), in kcal/mol. *^b^*From *G* values at 298.15K. | | |

**
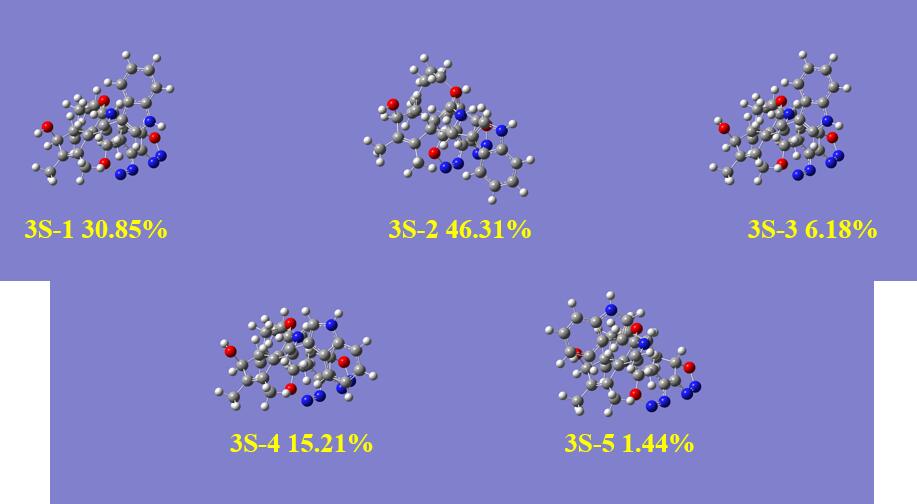
**

**Figure S30.** Structures and populations of the low-energy conformers of (3*S*, 4*R*, 7*S*, 9*R*,16*S*, 19*S*)-**3**.

**Table S2.** Cartesian coordinates for the low-energy reoptimized MMFF conformers of (3*S*, 4*R*, 7*S*, 9*R*,16*S*, 19*S*)-**3** at B3LYP/6-31G(d,p) level of theory in MeOH

| **3S-1** | | Standard Orientation  (Ångstroms) | | | |
| --- | --- | --- | --- | --- | --- |
| Center number | Atomic number | Atomic Type | X | Y | Z |
| 1. | 6. | 0. | -1.138185 | 3.038555 | -1.147640 |
| 2. | 6. | 0. | -1.412298 | 1.869922 | -0.221192 |
| 3. | 6. | 0. | -0.091718 | 1.177303 | 0.208833 |
| 4. | 6. | 0. | 0.825066 | 2.190949 | 0.979641 |
| 5. | 6. | 0. | 0.343121 | 3.657857 | 0.732440 |
| 6. | 6. | 0. | -0.233453 | 3.914244 | -0.660663 |
| 7. | 6. | 0. | 2.301612 | 2.108726 | 0.660591 |
| 8. | 6. | 0. | 0.639298 | 0.488923 | -0.969875 |
| 9. | 6. | 0. | 1.486691 | -0.567287 | -0.702436 |
| 10. | 6. | 0. | -6.313280 | -3.156478 | 2.067661 |
| 11. | 6. | 0. | -6.546416 | -3.145554 | 0.697733 |
| 12. | 6. | 0. | -5.968774 | -2.112735 | -0.047781 |
| 13. | 6. | 0. | -5.167623 | -1.098696 | 0.548080 |
| 14. | 6. | 0. | -4.955928 | -1.136014 | 1.939793 |
| 15. | 6. | 0. | -5.527125 | -2.161100 | 2.682919 |
| 16. | 7. | 0. | -6.027915 | -1.842450 | -1.398710 |
| 17. | 6. | 0. | -5.299286 | -0.699701 | -1.667312 |
| 18. | 6. | 0. | -4.749127 | -0.206385 | -0.509257 |
| 19. | 6. | 0. | -3.857810 | 0.998623 | -0.381585 |
| 20. | 6. | 0. | -0.668836 | 0.018874 | 1.054544 |
| 21. | 7. | 0. | -1.896000 | -0.248961 | 0.522719 |
| 22. | 6. | 0. | -2.354773 | 0.685324 | -0.519027 |
| 23. | 1. | 0. | -1.802519 | 2.297526 | 0.710589 |
| 24. | 8. | 0. | -0.133164 | -0.566354 | 1.986832 |
| 25. | 6. | 0. | 3.278133 | 1.593478 | 1.416393 |
| 26. | 6. | 0. | 3.144310 | 0.858913 | 2.726188 |
| 27. | 6. | 0. | 4.142533 | -0.335051 | 2.877572 |
| 28. | 6. | 0. | 3.863712 | -1.380242 | 1.812629 |
| 29. | 6. | 0. | 4.448242 | -1.683981 | 0.642144 |
| 30. | 6. | 0. | 3.752800 | -2.725854 | -0.231947 |
| 31. | 6. | 0. | 3.191677 | -2.205762 | -1.535002 |
| 32. | 6. | 0. | 2.227297 | -1.246714 | -1.699810 |
| 33. | 6. | 0. | 5.591104 | 0.145072 | 3.064406 |
| 34. | 6. | 0. | 5.680779 | -1.083334 | 0.017147 |
| 35. | 6. | 0. | -1.850075 | 3.137608 | -2.467763 |
| 36. | 6. | 0. | 0.259073 | 5.162143 | -1.348249 |
| 37. | 8. | 0. | -0.621171 | 3.935453 | 1.770762 |
| 38. | 7. | 0. | 3.818940 | -2.869745 | -2.578836 |
| 39. | 7. | 0. | 1.897275 | -0.901902 | -3.040147 |
| 40. | 8. | 0. | 4.682328 | -3.718560 | -0.733184 |
| 41. | 8. | 0. | 0.418779 | 0.956208 | -2.164303 |
| 42. | 7. | 0. | 4.643628 | -3.707598 | -2.151821 |
| 43. | 7. | 0. | 1.193491 | -0.079828 | -3.478006 |
| 44. | 1. | 0. | 0.674783 | 2.006378 | 2.046860 |
| 45. | 1. | 0. | 1.210089 | 4.316514 | 0.883089 |
| 46. | 1. | 0. | 2.587815 | 2.568541 | -0.286187 |
| 47. | 1. | 0. | 1.596457 | -0.886677 | 0.321769 |
| 48. | 1. | 0. | -6.746027 | -3.945468 | 2.675163 |
| 49. | 1. | 0. | -7.154484 | -3.911443 | 0.224803 |
| 50. | 1. | 0. | -4.356842 | -0.372731 | 2.428450 |
| 51. | 1. | 0. | -5.367961 | -2.198005 | 3.756215 |
| 52. | 1. | 0. | -6.538918 | -2.379189 | -2.079939 |
| 53. | 1. | 0. | -5.232042 | -0.317862 | -2.676675 |
| 54. | 1. | 0. | -4.023990 | 1.487647 | 0.586176 |
| 55. | 1. | 0. | -4.118943 | 1.731017 | -1.153072 |
| 56. | 1. | 0. | -2.451704 | -1.028276 | 0.848837 |
| 57. | 1. | 0. | -2.173393 | 0.257809 | -1.514393 |
| 58. | 1. | 0. | 4.292593 | 1.695978 | 1.032650 |
| 59. | 1. | 0. | 2.122916 | 0.484998 | 2.846443 |
| 60. | 1. | 0. | 3.333132 | 1.547663 | 3.563694 |
| 61. | 1. | 0. | 3.864470 | -0.818790 | 3.824298 |
| 62. | 1. | 0. | 2.958938 | -1.945786 | 2.044685 |
| 63. | 1. | 0. | 2.985037 | -3.248107 | 0.355834 |
| 64. | 1. | 0. | 5.673465 | 0.682082 | 4.015479 |
| 65. | 1. | 0. | 5.923678 | 0.832758 | 2.282511 |
| 66. | 1. | 0. | 6.289901 | -0.696147 | 3.094741 |
| 67. | 1. | 0. | 6.369198 | -1.877429 | -0.289442 |
| 68. | 1. | 0. | 6.209568 | -0.409579 | 0.685454 |
| 69. | 1. | 0. | 5.417827 | -0.523456 | -0.889333 |
| 70. | 1. | 0. | -2.929665 | 3.279834 | -2.329251 |
| 71. | 1. | 0. | -1.488196 | 3.965747 | -3.078852 |
| 72. | 1. | 0. | -1.709102 | 2.215792 | -3.040253 |
| 73. | 1. | 0. | -0.208733 | 5.332989 | -2.318961 |
| 74. | 1. | 0. | 1.344548 | 5.118665 | -1.506454 |
| 75. | 1. | 0. | 0.074903 | 6.054738 | -0.734228 |
| 76. | 1. | 0. | -1.051371 | 4.771924 | 1.546998 |

| **3S-2** | | Standard Orientation  (Ångstroms) | | | |
| --- | --- | --- | --- | --- | --- |
| Center number | Atom number | Type | X | Y | Z |
| 1. | 6. | 0. | 1.287565 | 2.660820 | 1.476584 |
| 2. | 6. | 0. | 1.333556 | 1.922477 | 0.153256 |
| 3. | 6. | 0. | -0.050835 | 1.310429 | -0.194478 |
| 4. | 6. | 0. | -1.124877 | 2.446240 | -0.325656 |
| 5. | 6. | 0. | -0.605560 | 3.766463 | 0.332819 |
| 6. | 6. | 0. | 0.288514 | 3.565118 | 1.557066 |
| 7. | 6. | 0. | -2.489827 | 2.127660 | 0.243975 |
| 8. | 6. | 0. | -0.478248 | 0.190149 | 0.785668 |
| 9. | 6. | 0. | -1.368511 | -0.770893 | 0.348496 |
| 10. | 6. | 0. | 6.670244 | -3.151812 | 0.472231 |
| 11. | 6. | 0. | 6.678281 | -2.833011 | -0.880175 |
| 12. | 6. | 0. | 5.956166 | -1.703912 | -1.281044 |
| 13. | 6. | 0. | 5.232066 | -0.897835 | -0.360256 |
| 14. | 6. | 0. | 5.246627 | -1.246642 | 1.002093 |
| 15. | 6. | 0. | 5.962875 | -2.365865 | 1.404961 |
| 16. | 7. | 0. | 5.786343 | -1.147343 | -2.532272 |
| 17. | 6. | 0. | 4.986720 | -0.024759 | -2.425890 |
| 18. | 6. | 0. | 4.620836 | 0.171473 | -1.115521 |
| 19. | 6. | 0. | 3.740395 | 1.269104 | -0.588176 |
| 20. | 6. | 0. | 0.312505 | 0.577188 | -1.506860 |
| 21. | 7. | 0. | 1.628392 | 0.243009 | -1.385489 |
| 22. | 6. | 0. | 2.322341 | 0.796594 | -0.212781 |
| 23. | 1. | 0. | 1.488761 | 2.683546 | -0.621716 |
| 24. | 8. | 0. | -0.423527 | 0.316850 | -2.449994 |
| 25. | 6. | 0. | -3.609507 | 1.827143 | -0.424209 |
| 26. | 6. | 0. | -3.773127 | 1.612538 | -1.907441 |
| 27. | 6. | 0. | -4.769479 | 0.462347 | -2.266746 |
| 28. | 6. | 0. | -4.248656 | -0.860407 | -1.733627 |
| 29. | 6. | 0. | -4.545246 | -1.598927 | -0.651515 |
| 30. | 6. | 0. | -3.665322 | -2.816910 | -0.378675 |
| 31. | 6. | 0. | -2.824194 | -2.741751 | 0.874244 |
| 32. | 6. | 0. | -1.850729 | -1.822238 | 1.164648 |
| 33. | 6. | 0. | -6.226716 | 0.845354 | -1.960367 |
| 34. | 6. | 0. | -5.601490 | -1.359609 | 0.396259 |
| 35. | 6. | 0. | 2.296566 | 2.351008 | 2.546678 |
| 36. | 6. | 0. | -0.030087 | 4.437820 | 2.744571 |
| 37. | 8. | 0. | 0.079512 | 4.484769 | -0.716395 |
| 38. | 7. | 0. | -3.192463 | -3.782583 | 1.713307 |
| 39. | 7. | 0. | -1.216045 | -1.945494 | 2.431904 |
| 40. | 8. | 0. | -4.452886 | -3.998055 | -0.086061 |
| 41. | 8. | 0. | 0.026082 | 0.218741 | 1.983664 |
| 42. | 7. | 0. | -4.089873 | -4.484536 | 1.197224 |
| 43. | 7. | 0. | -0.426943 | -1.279202 | 2.973843 |
| 44. | 1. | 0. | -1.221538 | 2.667655 | -1.392111 |
| 45. | 1. | 0. | -1.487057 | 4.351398 | 0.630995 |
| 46. | 1. | 0. | -2.558067 | 2.200288 | 1.330144 |
| 47. | 1. | 0. | -1.723751 | -0.714517 | -0.668060 |
| 48. | 1. | 0. | 7.220225 | -4.022147 | 0.817222 |
| 49. | 1. | 0. | 7.225354 | -3.438166 | -1.597409 |
| 50. | 1. | 0. | 4.707811 | -0.648562 | 1.731616 |
| 51. | 1. | 0. | 5.980284 | -2.643805 | 2.454385 |
| 52. | 1. | 0. | 6.195720 | -1.490643 | -3.385339 |
| 53. | 1. | 0. | 4.751830 | 0.567833 | -3.299460 |
| 54. | 1. | 0. | 3.651226 | 2.064926 | -1.337047 |
| 55. | 1. | 0. | 4.198101 | 1.719709 | 0.300605 |
| 56. | 1. | 0. | 2.104037 | -0.311428 | -2.083859 |
| 57. | 1. | 0. | 2.388787 | 0.038832 | 0.578553 |
| 58. | 1. | 0. | -4.510536 | 1.699426 | 0.174702 |
| 59. | 1. | 0. | -2.803310 | 1.398536 | -2.366505 |
| 60. | 1. | 0. | -4.154753 | 2.530703 | -2.379538 |
| 61. | 1. | 0. | -4.709656 | 0.366834 | -3.359815 |
| 62. | 1. | 0. | -3.421143 | -1.231304 | -2.341933 |
| 63. | 1. | 0. | -3.048774 | -3.031898 | -1.262662 |
| 64. | 1. | 0. | -6.529968 | 1.667379 | -2.617648 |
| 65. | 1. | 0. | -6.376758 | 1.188922 | -0.933746 |
| 66. | 1. | 0. | -6.906239 | 0.006777 | -2.138971 |
| 67. | 1. | 0. | -6.203168 | -2.263946 | 0.532825 |
| 68. | 1. | 0. | -6.267388 | -0.538014 | 0.148040 |
| 69. | 1. | 0. | -5.137454 | -1.137919 | 1.365695 |
| 70. | 1. | 0. | 3.315278 | 2.591116 | 2.216418 |
| 71. | 1. | 0. | 2.114450 | 2.903604 | 3.469488 |
| 72. | 1. | 0. | 2.270790 | 1.283592 | 2.787614 |
| 73. | 1. | 0. | -0.012408 | 5.502748 | 2.473882 |
| 74. | 1. | 0. | 0.661755 | 4.301713 | 3.576973 |
| 75. | 1. | 0. | -1.041991 | 4.234141 | 3.118486 |
| 76. | 1. | 0. | 0.543155 | 5.225148 | -0.301948 |

| **3S-3** | | Standard Orientation  (Ångstroms) | | | |
| --- | --- | --- | --- | --- | --- |
| Center number | Atom number | Type | X | Y | Z |
| 1. | 6. | 0. | -1.132525 | 3.023293 | -1.188066 |
| 2. | 6. | 0. | -1.407699 | 1.870120 | -0.242304 |
| 3. | 6. | 0. | -0.090293 | 1.181064 | 0.196004 |
| 4. | 6. | 0. | 0.834737 | 2.206494 | 0.936250 |
| 5. | 6. | 0. | 0.349425 | 3.680019 | 0.673163 |
| 6. | 6. | 0. | -0.234563 | 3.911631 | -0.716861 |
| 7. | 6. | 0. | 2.306311 | 2.121838 | 0.596987 |
| 8. | 6. | 0. | 0.630979 | 0.459788 | -0.968630 |
| 9. | 6. | 0. | 1.475179 | -0.593879 | -0.680588 |
| 10. | 6. | 0. | -6.313276 | -3.125714 | 2.112643 |
| 11. | 6. | 0. | -6.542743 | -3.133494 | 0.742042 |
| 12. | 6. | 0. | -5.964732 | -2.110042 | -0.015921 |
| 13. | 6. | 0. | -5.166434 | -1.086765 | 0.568160 |
| 14. | 6. | 0. | -4.959135 | -1.104836 | 1.960919 |
| 15. | 6. | 0. | -5.530626 | -2.120703 | 2.716492 |
| 16. | 7. | 0. | -6.020648 | -1.858292 | -1.370437 |
| 17. | 6. | 0. | -5.292687 | -0.718337 | -1.652593 |
| 18. | 6. | 0. | -4.745868 | -0.208603 | -0.500121 |
| 19. | 6. | 0. | -3.854492 | 0.997981 | -0.388186 |
| 20. | 6. | 0. | -0.669945 | 0.048676 | 1.074162 |
| 21. | 7. | 0. | -1.894954 | -0.232651 | 0.546815 |
| 22. | 6. | 0. | -2.351856 | 0.680697 | -0.516425 |
| 23. | 1. | 0. | -1.800777 | 2.315767 | 0.680068 |
| 24. | 8. | 0. | -0.137357 | -0.503158 | 2.029260 |
| 25. | 6. | 0. | 3.298745 | 1.633968 | 1.350704 |
| 26. | 6. | 0. | 3.190172 | 0.929659 | 2.679763 |
| 27. | 6. | 0. | 4.196359 | -0.255744 | 2.845079 |
| 28. | 6. | 0. | 3.897702 | -1.332720 | 1.818173 |
| 29. | 6. | 0. | 4.459529 | -1.673477 | 0.646956 |
| 30. | 6. | 0. | 3.746519 | -2.741713 | -0.179948 |
| 31. | 6. | 0. | 3.164170 | -2.261331 | -1.488883 |
| 32. | 6. | 0. | 2.200127 | -1.304620 | -1.667741 |
| 33. | 6. | 0. | 5.646700 | 0.233742 | 2.986449 |
| 34. | 6. | 0. | 5.680167 | -1.094390 | -0.020586 |
| 35. | 6. | 0. | -1.853941 | 3.106570 | -2.504081 |
| 36. | 6. | 0. | 0.208669 | 5.180813 | -1.393830 |
| 37. | 8. | 0. | -0.658090 | 4.077610 | 1.621461 |
| 38. | 7. | 0. | 3.772243 | -2.959706 | -2.521713 |
| 39. | 7. | 0. | 1.851245 | -0.999446 | -3.013288 |
| 40. | 8. | 0. | 4.664733 | -3.752380 | -0.664726 |
| 41. | 8. | 0. | 0.403314 | 0.896521 | -2.172768 |
| 42. | 7. | 0. | 4.601959 | -3.785995 | -2.082886 |
| 43. | 7. | 0. | 1.146678 | -0.184980 | -3.464462 |
| 44. | 1. | 0. | 0.708111 | 2.016197 | 2.007892 |
| 45. | 1. | 0. | 1.225824 | 4.331373 | 0.799669 |
| 46. | 1. | 0. | 2.573101 | 2.560597 | -0.365114 |
| 47. | 1. | 0. | 1.591533 | -0.888740 | 0.350357 |
| 48. | 1. | 0. | -6.746564 | -3.907080 | 2.729540 |
| 49. | 1. | 0. | -7.148529 | -3.906560 | 0.277937 |
| 50. | 1. | 0. | -4.364272 | -0.332812 | 2.441027 |
| 51. | 1. | 0. | -5.375042 | -2.142401 | 3.790751 |
| 52. | 1. | 0. | -6.530588 | -2.403860 | -2.045426 |
| 53. | 1. | 0. | -5.223287 | -0.350001 | -2.666804 |
| 54. | 1. | 0. | -4.023429 | 1.503932 | 0.570252 |
| 55. | 1. | 0. | -4.111305 | 1.718341 | -1.172093 |
| 56. | 1. | 0. | -2.455416 | -0.999137 | 0.894723 |
| 57. | 1. | 0. | -2.169045 | 0.230966 | -1.501389 |
| 58. | 1. | 0. | 4.306530 | 1.734434 | 0.949668 |
| 59. | 1. | 0. | 2.173083 | 0.551724 | 2.824472 |
| 60. | 1. | 0. | 3.388991 | 1.638224 | 3.498675 |
| 61. | 1. | 0. | 3.940279 | -0.711509 | 3.811755 |
| 62. | 1. | 0. | 2.997851 | -1.890750 | 2.084833 |
| 63. | 1. | 0. | 2.988084 | -3.241963 | 0.438283 |
| 64. | 1. | 0. | 5.746878 | 0.803771 | 3.916404 |
| 65. | 1. | 0. | 5.963031 | 0.893344 | 2.174174 |
| 66. | 1. | 0. | 6.347276 | -0.605201 | 3.032598 |
| 67. | 1. | 0. | 6.358048 | -1.899549 | -0.321621 |
| 68. | 1. | 0. | 6.227377 | -0.405991 | 0.617417 |
| 69. | 1. | 0. | 5.399040 | -0.557290 | -0.935315 |
| 70. | 1. | 0. | -2.927249 | 3.285686 | -2.359125 |
| 71. | 1. | 0. | -1.473465 | 3.908221 | -3.138621 |
| 72. | 1. | 0. | -1.745772 | 2.167401 | -3.055238 |
| 73. | 1. | 0. | -0.257377 | 5.336561 | -2.368048 |
| 74. | 1. | 0. | 1.296756 | 5.192749 | -1.539873 |
| 75. | 1. | 0. | -0.033599 | 6.048207 | -0.766061 |
| 76. | 1. | 0. | -0.270589 | 4.004188 | 2.505115 |

| **3S -4** | | Standard Orientation  (Ångstroms) | | | |
| --- | --- | --- | --- | --- | --- |
| Center number | Atom number | Type | X | Y | Z |
| 1. | 6. | 0. | 1.278230 | 2.666740 | 1.481341 |
| 2. | 6. | 0. | 1.330353 | 1.927630 | 0.158395 |
| 3. | 6. | 0. | -0.048767 | 1.311006 | -0.193198 |
| 4. | 6. | 0. | -1.125748 | 2.441878 | -0.319454 |
| 5. | 6. | 0. | -0.609986 | 3.774641 | 0.340487 |
| 6. | 6. | 0. | 0.284759 | 3.574571 | 1.559582 |
| 7. | 6. | 0. | -2.487534 | 2.120068 | 0.255205 |
| 8. | 6. | 0. | -0.473051 | 0.183836 | 0.780035 |
| 9. | 6. | 0. | -1.360259 | -0.778036 | 0.337959 |
| 10. | 6. | 0. | 6.640408 | -3.174283 | 0.460077 |
| 11. | 6. | 0. | 6.664495 | -2.836712 | -0.887577 |
| 12. | 6. | 0. | 5.951490 | -1.699014 | -1.280355 |
| 13. | 6. | 0. | 5.220779 | -0.902724 | -0.356290 |
| 14. | 6. | 0. | 5.219106 | -1.270479 | 1.001160 |
| 15. | 6. | 0. | 5.926243 | -2.398331 | 1.395997 |
| 16. | 7. | 0. | 5.797556 | -1.124452 | -2.525507 |
| 17. | 6. | 0. | 5.001839 | 0.000317 | -2.411900 |
| 18. | 6. | 0. | 4.622359 | 0.179842 | -1.103029 |
| 19. | 6. | 0. | 3.741926 | 1.274920 | -0.570145 |
| 20. | 6. | 0. | 0.318892 | 0.589492 | -1.510692 |
| 21. | 7. | 0. | 1.633134 | 0.254158 | -1.386516 |
| 22. | 6. | 0. | 2.321253 | 0.802645 | -0.206319 |
| 23. | 1. | 0. | 1.491962 | 2.689775 | -0.614657 |
| 24. | 8. | 0. | -0.414269 | 0.345472 | -2.461410 |
| 25. | 6. | 0. | -3.612956 | 1.825759 | -0.406725 |
| 26. | 6. | 0. | -3.786777 | 1.613658 | -1.889557 |
| 27. | 6. | 0. | -4.784799 | 0.463833 | -2.245600 |
| 28. | 6. | 0. | -4.257085 | -0.860961 | -1.724470 |
| 29. | 6. | 0. | -4.542803 | -1.606031 | -0.644092 |
| 30. | 6. | 0. | -3.659182 | -2.824864 | -0.387480 |
| 31. | 6. | 0. | -2.807696 | -2.756835 | 0.858854 |
| 32. | 6. | 0. | -1.834798 | -1.836863 | 1.149146 |
| 33. | 6. | 0. | -6.239815 | 0.843209 | -1.924624 |
| 34. | 6. | 0. | -5.589596 | -1.374024 | 0.414616 |
| 35. | 6. | 0. | 2.292398 | 2.366904 | 2.549321 |
| 36. | 6. | 0. | 0.001601 | 4.493279 | 2.717949 |
| 37. | 8. | 0. | 0.139055 | 4.567647 | -0.599605 |
| 38. | 7. | 0. | -3.166818 | -3.804977 | 1.693023 |
| 39. | 7. | 0. | -1.191067 | -1.968852 | 2.411349 |
| 40. | 8. | 0. | -4.442935 | -4.008514 | -0.096871 |
| 41. | 8. | 0. | 0.032514 | 0.206913 | 1.977245 |
| 42. | 7. | 0. | -4.067440 | -4.504509 | 1.179959 |
| 43. | 7. | 0. | -0.402667 | -1.301778 | 2.953910 |
| 44. | 1. | 0. | -1.233785 | 2.641150 | -1.391477 |
| 45. | 1. | 0. | -1.499430 | 4.343091 | 0.646988 |
| 46. | 1. | 0. | -2.547755 | 2.190130 | 1.341839 |
| 47. | 1. | 0. | -1.716928 | -0.717755 | -0.677966 |
| 48. | 1. | 0. | 7.183126 | -4.051604 | 0.798837 |
| 49. | 1. | 0. | 7.216968 | -3.434044 | -1.607240 |
| 50. | 1. | 0. | 4.675508 | -0.679879 | 1.733208 |
| 51. | 1. | 0. | 5.931386 | -2.690708 | 2.441617 |
| 52. | 1. | 0. | 6.217292 | -1.455891 | -3.378213 |
| 53. | 1. | 0. | 4.780099 | 0.606816 | -3.279338 |
| 54. | 1. | 0. | 3.659603 | 2.078630 | -1.311204 |
| 55. | 1. | 0. | 4.194660 | 1.716129 | 0.325623 |
| 56. | 1. | 0. | 2.114444 | -0.290800 | -2.088575 |
| 57. | 1. | 0. | 2.381115 | 0.040750 | 0.581253 |
| 58. | 1. | 0. | -4.510569 | 1.699215 | 0.197335 |
| 59. | 1. | 0. | -2.820119 | 1.399253 | -2.355677 |
| 60. | 1. | 0. | -4.173146 | 2.531915 | -2.358407 |
| 61. | 1. | 0. | -4.734577 | 0.374389 | -3.339698 |
| 62. | 1. | 0. | -3.435211 | -1.227790 | -2.342789 |
| 63. | 1. | 0. | -3.049804 | -3.032627 | -1.278211 |
| 64. | 1. | 0. | -6.549949 | 1.668913 | -2.574117 |
| 65. | 1. | 0. | -6.382200 | 1.179492 | -0.894566 |
| 66. | 1. | 0. | -6.919264 | 0.004673 | -2.103273 |
| 67. | 1. | 0. | -6.187652 | -2.280521 | 0.552494 |
| 68. | 1. | 0. | -6.259755 | -0.552920 | 0.176411 |
| 69. | 1. | 0. | -5.116890 | -1.155750 | 1.380585 |
| 70. | 1. | 0. | 3.303825 | 2.646970 | 2.227812 |
| 71. | 1. | 0. | 2.088642 | 2.897013 | 3.480677 |
| 72. | 1. | 0. | 2.300517 | 1.295248 | 2.772235 |
| 73. | 1. | 0. | 0.686689 | 4.354900 | 3.555650 |
| 74. | 1. | 0. | -1.019153 | 4.350032 | 3.096166 |
| 75. | 1. | 0. | 0.073977 | 5.540629 | 2.397053 |
| 76. | 1. | 0. | -0.449111 | 4.771472 | -1.340460 |

| **3S-5** | | Standard Orientation  (Ångstroms) | | | |
| --- | --- | --- | --- | --- | --- |
| Center number | Atom number | Type | X | Y | Z |
| 1. | 6. | 0. | 1.930971 | -1.229632 | 1.661069 |
| 2. | 6. | 0. | 1.701643 | -0.845205 | 0.212876 |
| 3. | 6. | 0. | 0.309421 | -0.185447 | 0.023459 |
| 4. | 6. | 0. | 0.225417 | 1.126988 | 0.883249 |
| 5. | 6. | 0. | 1.384831 | 1.162567 | 1.939100 |
| 6. | 6. | 0. | 1.744129 | -0.205200 | 2.521171 |
| 7. | 6. | 0. | -1.089407 | 1.357468 | 1.593485 |
| 8. | 6. | 0. | -0.871345 | -1.150592 | 0.287254 |
| 9. | 6. | 0. | -2.090960 | -0.894718 | -0.307445 |
| 10. | 6. | 0. | 6.809053 | 1.542107 | 0.467002 |
| 11. | 6. | 0. | 6.539239 | 1.729066 | -0.882484 |
| 12. | 6. | 0. | 5.646895 | 0.841628 | -1.493107 |
| 13. | 6. | 0. | 5.020361 | -0.221742 | -0.785692 |
| 14. | 6. | 0. | 5.322683 | -0.388777 | 0.578825 |
| 15. | 6. | 0. | 6.208263 | 0.490855 | 1.190749 |
| 16. | 7. | 0. | 5.202005 | 0.781190 | -2.796127 |
| 17. | 6. | 0. | 4.320096 | -0.272293 | -2.926266 |
| 18. | 6. | 0. | 4.170134 | -0.919781 | -1.723528 |
| 19. | 6. | 0. | 3.282606 | -2.104270 | -1.458246 |
| 20. | 6. | 0. | 0.328764 | 0.012879 | -1.508672 |
| 21. | 7. | 0. | 1.104889 | -0.998890 | -1.998745 |
| 22. | 6. | 0. | 1.837610 | -1.789348 | -0.995713 |
| 23. | 1. | 0. | 2.398144 | -0.030925 | -0.009922 |
| 24. | 8. | 0. | -0.254361 | 0.863671 | -2.166721 |
| 25. | 6. | 0. | -2.044093 | 2.241816 | 1.282130 |
| 26. | 6. | 0. | -2.102645 | 3.152038 | 0.081557 |
| 27. | 6. | 0. | -3.540773 | 3.324526 | -0.507081 |
| 28. | 6. | 0. | -4.058555 | 1.988440 | -1.007997 |
| 29. | 6. | 0. | -4.904313 | 1.078009 | -0.498323 |
| 30. | 6. | 0. | -5.040862 | -0.241735 | -1.254434 |
| 31. | 6. | 0. | -4.524307 | -1.460243 | -0.525739 |
| 32. | 6. | 0. | -3.241542 | -1.695066 | -0.105200 |
| 33. | 6. | 0. | -4.446325 | 4.154224 | 0.417484 |
| 34. | 6. | 0. | -5.704727 | 1.139927 | 0.777318 |
| 35. | 6. | 0. | 2.301287 | -2.641371 | 2.022968 |
| 36. | 6. | 0. | 1.863891 | -0.264431 | 4.022241 |
| 37. | 8. | 0. | 2.505335 | 1.790807 | 1.286886 |
| 38. | 7. | 0. | -5.579554 | -2.341612 | -0.342184 |
| 39. | 7. | 0. | -3.018947 | -2.909861 | 0.601189 |
| 40. | 8. | 0. | -6.427850 | -0.621371 | -1.434891 |
| 41. | 8. | 0. | -0.649464 | -2.161768 | 1.075168 |
| 42. | 7. | 0. | -6.635946 | -1.893285 | -0.839173 |
| 43. | 7. | 0. | -2.073760 | -3.329102 | 1.143025 |
| 44. | 1. | 0. | 0.422262 | 1.962062 | 0.205584 |
| 45. | 1. | 0. | 1.055935 | 1.818617 | 2.757273 |
| 46. | 1. | 0. | -1.238439 | 0.750264 | 2.487256 |
| 47. | 1. | 0. | -2.175304 | -0.034981 | -0.953067 |
| 48. | 1. | 0. | 7.496596 | 2.213565 | 0.972121 |
| 49. | 1. | 0. | 7.002650 | 2.536572 | -1.441933 |
| 50. | 1. | 0. | 4.873479 | -1.195231 | 1.149896 |
| 51. | 1. | 0. | 6.452212 | 0.362752 | 2.241370 |
| 52. | 1. | 0. | 5.461613 | 1.416437 | -3.532560 |
| 53. | 1. | 0. | 3.870396 | -0.489273 | -3.885358 |
| 54. | 1. | 0. | 3.738973 | -2.754207 | -0.703245 |
| 55. | 1. | 0. | 3.210554 | -2.718163 | -2.365812 |
| 56. | 1. | 0. | 1.245524 | -1.093441 | -2.994207 |
| 57. | 1. | 0. | 1.324674 | -2.745244 | -0.823803 |
| 58. | 1. | 0. | -2.892153 | 2.300908 | 1.963528 |
| 59. | 1. | 0. | -1.439245 | 2.780605 | -0.705622 |
| 60. | 1. | 0. | -1.741844 | 4.155611 | 0.353803 |
| 61. | 1. | 0. | -3.404962 | 3.930556 | -1.413690 |
| 62. | 1. | 0. | -3.578797 | 1.707224 | -1.947774 |
| 63. | 1. | 0. | -4.584802 | -0.145254 | -2.249674 |
| 64. | 1. | 0. | -4.059434 | 5.177054 | 0.479109 |
| 65. | 1. | 0. | -4.489259 | 3.769268 | 1.439459 |
| 66. | 1. | 0. | -5.468879 | 4.206590 | 0.032100 |
| 67. | 1. | 0. | -6.745846 | 0.867821 | 0.576607 |
| 68. | 1. | 0. | -5.690451 | 2.122653 | 1.240233 |
| 69. | 1. | 0. | -5.322173 | 0.416179 | 1.508262 |
| 70. | 1. | 0. | 3.242018 | -2.947216 | 1.547836 |
| 71. | 1. | 0. | 2.417800 | -2.782515 | 3.098537 |
| 72. | 1. | 0. | 1.523731 | -3.329767 | 1.677638 |
| 73. | 1. | 0. | 2.182449 | -1.240662 | 4.391148 |
| 74. | 1. | 0. | 0.904080 | -0.027367 | 4.499634 |
| 75. | 1. | 0. | 2.582192 | 0.480527 | 4.389864 |
| 76. | 1. | 0. | 3.321595 | 1.433055 | 1.663915 |

**Table S3.** Gibbs free energies*^a^* and equilibrium populations*^b^* of low-energy conformers of (3*S*, 4*R*, 7*S*, 9*R*, 16*S*, 19*R*)-**3**

| Conformers | In MeOH | |
| --- | --- | --- |
|  | *G^a^* | *P* (%)^b^ |
| **3R**.**-1** | -1172543.38442007 | 18.94 |
| **3R**.**-2** | -1172541.779877 | 1.26 |
| **3R**.**-3** | -1172544.05962083 | 59.27 |
| **3R**.**-4** | -1172542.55234181 | 4.64 |
| **3R**.**-5** | -1172543.28025341 | 15.89 |
|  |  |  |
| *^a^*B3LYP/6-31G(d,p), in kcal/mol. *^b^*From *G* values at 298.15K. | | |

**
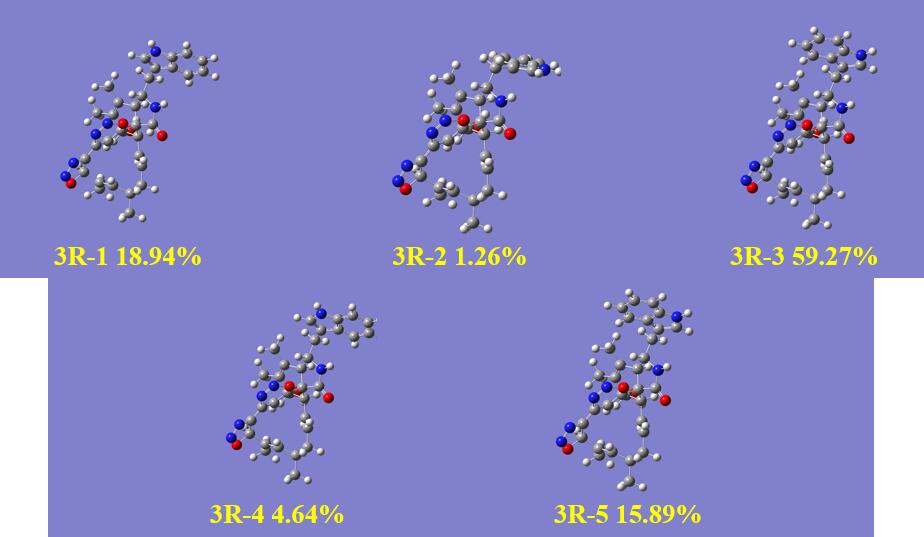
**

**Figure S31.** Structures and populations of the low-energy conformers of (3*S*, 4*R*, 7*S*, 9*R*,16*S*, 19*R*)-**3**

**Table S4.** Cartesian coordinates for the low-energy reoptimized MMFF conformers of (3*S*, 4*R*, 7*S*, 9*R*, 16*S*, 19*R*)-**3** at B3LYP/6-31G(d,p) level of theory in MeOH.

| **3R-1** | | Standard Orientation  (Ångstroms) | | | |
| --- | --- | --- | --- | --- | --- |
| Center number | Atomic number | Atomic Type | X | Y | Z |
| 1. | 6. | 0. | 0.915704 | 2.810789 | -0.831685 |
| 2. | 6. | 0. | 1.434319 | 1.384604 | -0.902106 |
| 3. | 6. | 0. | 0.276266 | 0.356021 | -0.709562 |
| 4. | 6. | 0. | -0.588285 | 0.487266 | -1.999420 |
| 5. | 6. | 0. | -1.101040 | 1.965619 | -2.099570 |
| 6. | 6. | 0. | -0.271083 | 3.057022 | -1.422792 |
| 7. | 6. | 0. | -1.761199 | -0.430913 | -2.319619 |
| 8. | 6. | 0. | -0.496828 | 0.605095 | 0.606700 |
| 9. | 6. | 0. | -1.860993 | 0.421967 | 0.703290 |
| 10. | 6. | 0. | 7.241056 | -3.066384 | 0.196980 |
| 11. | 6. | 0. | 7.482722 | -2.056434 | 1.120258 |
| 12. | 6. | 0. | 6.711044 | -0.893916 | 1.023243 |
| 13. | 6. | 0. | 5.708452 | -0.725031 | 0.027386 |
| 14. | 6. | 0. | 5.492311 | -1.766436 | -0.895244 |
| 15. | 6. | 0. | 6.256403 | -2.922431 | -0.801588 |
| 16. | 7. | 0. | 6.729838 | 0.258122 | 1.780834 |
| 17. | 6. | 0. | 5.782704 | 1.138374 | 1.294967 |
| 18. | 6. | 0. | 5.127420 | 0.584349 | 0.222172 |
| 19. | 6. | 0. | 4.007948 | 1.212592 | -0.562361 |
| 20. | 6. | 0. | 1.092442 | -0.953041 | -0.463772 |
| 21. | 7. | 0. | 2.342616 | -0.566979 | -0.091166 |
| 22. | 6. | 0. | 2.601253 | 0.871038 | -0.025780 |
| 23. | 1. | 0. | 1.795663 | 1.214672 | -1.928021 |
| 24. | 8. | 0. | 0.704794 | -2.109616 | -0.532198 |
| 25. | 6. | 0. | -2.004798 | -1.683602 | -1.922417 |
| 26. | 6. | 0. | -3.265126 | -2.438509 | -2.255125 |
| 27. | 6. | 0. | -4.000547 | -2.963082 | -0.983984 |
| 28. | 6. | 0. | -4.235771 | -1.868859 | 0.049816 |
| 29. | 6. | 0. | -5.021441 | -0.782863 | -0.002153 |
| 30. | 6. | 0. | -5.005694 | 0.238922 | 1.132510 |
| 31. | 6. | 0. | -3.899639 | 0.213059 | 2.152218 |
| 32. | 6. | 0. | -2.555079 | 0.374221 | 1.941580 |
| 33. | 6. | 0. | -5.254039 | -3.781640 | -1.345386 |
| 34. | 6. | 0. | -5.962907 | -0.402508 | -1.117328 |
| 35. | 6. | 0. | 1.751978 | 3.843377 | -0.121712 |
| 36. | 6. | 0. | -0.958758 | 4.401231 | -1.470916 |
| 37. | 8. | 0. | -1.360398 | 2.291434 | -3.474062 |
| 38. | 7. | 0. | -4.471805 | 0.055703 | 3.409278 |
| 39. | 7. | 0. | -1.736126 | 0.400200 | 3.106463 |
| 40. | 8. | 0. | -6.166014 | 0.076935 | 2.000499 |
| 41. | 8. | 0. | 0.248989 | 0.907753 | 1.632951 |
| 42. | 7. | 0. | -5.718313 | -0.023847 | 3.332253 |
| 43. | 7. | 0. | -0.593658 | 0.599037 | 3.238530 |
| 44. | 1. | 0. | 0.131484 | 0.368151 | -2.824389 |
| 45. | 1. | 0. | -2.098387 | 2.005312 | -1.644962 |
| 46. | 1. | 0. | -2.477796 | 0.050018 | -2.987120 |
| 47. | 1. | 0. | -2.407346 | 0.110360 | -0.169776 |
| 48. | 1. | 0. | 7.821621 | -3.982608 | 0.245507 |
| 49. | 1. | 0. | 8.242681 | -2.166996 | 1.888540 |
| 50. | 1. | 0. | 4.737188 | -1.669548 | -1.669983 |
| 51. | 1. | 0. | 6.094317 | -3.730818 | -1.507969 |
| 52. | 1. | 0. | 7.345865 | 0.437350 | 2.556487 |
| 53. | 1. | 0. | 5.649780 | 2.108051 | 1.754496 |
| 54. | 1. | 0. | 4.067071 | 0.901299 | -1.613149 |
| 55. | 1. | 0. | 4.123537 | 2.301238 | -0.549806 |
| 56. | 1. | 0. | 2.998891 | -1.253969 | 0.255413 |
| 57. | 1. | 0. | 2.516469 | 1.218011 | 1.008612 |
| 58. | 1. | 0. | -1.285295 | -2.190984 | -1.283421 |
| 59. | 1. | 0. | -3.024553 | -3.317640 | -2.871006 |
| 60. | 1. | 0. | -3.937556 | -1.810958 | -2.852396 |
| 61. | 1. | 0. | -3.303008 | -3.664141 | -0.508018 |
| 62. | 1. | 0. | -3.626130 | -1.973992 | 0.945216 |
| 63. | 1. | 0. | -5.085111 | 1.243862 | 0.687245 |
| 64. | 1. | 0. | -4.979610 | -4.632090 | -1.978794 |
| 65. | 1. | 0. | -5.996826 | -3.191666 | -1.889125 |
| 66. | 1. | 0. | -5.737324 | -4.173458 | -0.445381 |
| 67. | 1. | 0. | -6.994603 | -0.356547 | -0.747182 |
| 68. | 1. | 0. | -5.933635 | -1.099374 | -1.953162 |
| 69. | 1. | 0. | -5.722436 | 0.596285 | -1.504097 |
| 70. | 1. | 0. | 1.248290 | 4.806144 | -0.036304 |
| 71. | 1. | 0. | 1.993424 | 3.507076 | 0.892787 |
| 72. | 1. | 0. | 2.705011 | 4.014510 | -0.638535 |
| 73. | 1. | 0. | -1.361705 | 4.573060 | -2.473230 |
| 74. | 1. | 0. | -0.303821 | 5.235770 | -1.218688 |
| 75. | 1. | 0. | -1.811890 | 4.423828 | -0.779080 |
| 76. | 1. | 0. | -0.503865 | 2.332717 | -3.923158 |

| **3R-2** | | Standard Orientation  (Ångstroms) | | | |
| --- | --- | --- | --- | --- | --- |
| Center number | Atom number | Type | X | Y | Z |
| 1. | 6. | 0. | 1.591137 | -1.731976 | 1.201315 |
| 2. | 6. | 0. | 1.805179 | -0.990815 | -0.105458 |
| 3. | 6. | 0. | 0.498931 | -0.256157 | -0.539278 |
| 4. | 6. | 0. | 0.379778 | 0.928687 | 0.466109 |
| 5. | 6. | 0. | 0.386296 | 0.358607 | 1.933199 |
| 6. | 6. | 0. | 0.924258 | -1.054999 | 2.157480 |
| 7. | 6. | 0. | -0.713562 | 1.983461 | 0.375897 |
| 8. | 6. | 0. | -0.706322 | -1.215547 | -0.567312 |
| 9. | 6. | 0. | -1.950647 | -0.874286 | -0.090312 |
| 10. | 6. | 0. | 6.230635 | 1.827194 | 1.887313 |
| 11. | 6. | 0. | 6.349545 | 2.185607 | 0.550448 |
| 12. | 6. | 0. | 5.794682 | 1.324709 | -0.402379 |
| 13. | 6. | 0. | 5.126589 | 0.118905 | -0.047993 |
| 14. | 6. | 0. | 5.029948 | -0.217049 | 1.316168 |
| 15. | 6. | 0. | 5.577766 | 0.636057 | 2.266424 |
| 16. | 7. | 0. | 5.758627 | 1.420749 | -1.777033 |
| 17. | 6. | 0. | 5.093408 | 0.326257 | -2.291431 |
| 18. | 6. | 0. | 4.682398 | -0.503221 | -1.276250 |
| 19. | 6. | 0. | 3.910664 | -1.783706 | -1.440885 |
| 20. | 6. | 0. | 0.820501 | 0.056246 | -2.033348 |
| 21. | 7. | 0. | 1.843446 | -0.767506 | -2.393388 |
| 22. | 6. | 0. | 2.363874 | -1.672947 | -1.371059 |
| 23. | 1. | 0. | 2.520908 | -0.183530 | 0.085719 |
| 24. | 8. | 0. | 0.252422 | 0.836958 | -2.781884 |
| 25. | 6. | 0. | -1.429065 | 2.382509 | -0.679771 |
| 26. | 6. | 0. | -2.549291 | 3.386969 | -0.606903 |
| 27. | 6. | 0. | -3.879244 | 2.841940 | -1.210248 |
| 28. | 6. | 0. | -4.270993 | 1.487094 | -0.632466 |
| 29. | 6. | 0. | -4.658141 | 1.152720 | 0.607458 |
| 30. | 6. | 0. | -4.887041 | -0.310321 | 0.978684 |
| 31. | 6. | 0. | -4.398049 | -1.402871 | 0.066639 |
| 32. | 6. | 0. | -3.111103 | -1.662639 | -0.326267 |
| 33. | 6. | 0. | -4.997821 | 3.899907 | -1.181737 |
| 34. | 6. | 0. | -4.887764 | 2.091464 | 1.765261 |
| 35. | 6. | 0. | 2.081442 | -3.150647 | 1.327172 |
| 36. | 6. | 0. | 0.600034 | -1.574074 | 3.537283 |
| 37. | 8. | 0. | 1.026276 | 1.296560 | 2.811934 |
| 38. | 7. | 0. | -5.489163 | -2.185578 | -0.295082 |
| 39. | 7. | 0. | -2.922218 | -2.814916 | -1.135281 |
| 40. | 8. | 0. | -6.309922 | -0.623147 | 1.024811 |
| 41. | 8. | 0. | -0.459765 | -2.361508 | -1.154339 |
| 42. | 7. | 0. | -6.544359 | -1.759382 | 0.224230 |
| 43. | 7. | 0. | -1.943311 | -3.287591 | -1.573706 |
| 44. | 1. | 0. | 1.333516 | 1.466649 | 0.363093 |
| 45. | 1. | 0. | -0.650760 | 0.352365 | 2.290430 |
| 46. | 1. | 0. | -0.893992 | 2.465986 | 1.338129 |
| 47. | 1. | 0. | -2.098907 | 0.107113 | 0.324982 |
| 48. | 1. | 0. | 6.649413 | 2.473549 | 2.652566 |
| 49. | 1. | 0. | 6.853570 | 3.101904 | 0.256687 |
| 50. | 1. | 0. | 4.532175 | -1.130364 | 1.627691 |
| 51. | 1. | 0. | 5.510016 | 0.379901 | 3.319613 |
| 52. | 1. | 0. | 6.154374 | 2.169276 | -2.321490 |
| 53. | 1. | 0. | 4.967615 | 0.217512 | -3.359706 |
| 54. | 1. | 0. | 4.230070 | -2.515405 | -0.690447 |
| 55. | 1. | 0. | 4.160600 | -2.234168 | -2.410284 |
| 56. | 1. | 0. | 2.100999 | -0.828322 | -3.368398 |
| 57. | 1. | 0. | 1.943044 | -2.674389 | -1.510561 |
| 58. | 1. | 0. | -1.234473 | 1.937820 | -1.653711 |
| 59. | 1. | 0. | -2.284269 | 4.288395 | -1.179120 |
| 60. | 1. | 0. | -2.703410 | 3.707877 | 0.430391 |
| 61. | 1. | 0. | -3.665087 | 2.651594 | -2.269765 |
| 62. | 1. | 0. | -4.164455 | 0.664369 | -1.336711 |
| 63. | 1. | 0. | -4.499049 | -0.467018 | 1.998401 |
| 64. | 1. | 0. | -4.686324 | 4.792428 | -1.735292 |
| 65. | 1. | 0. | -5.251780 | 4.214403 | -0.165891 |
| 66. | 1. | 0. | -5.910586 | 3.515496 | -1.646625 |
| 67. | 1. | 0. | -5.930225 | 2.033881 | 2.102049 |
| 68. | 1. | 0. | -4.668050 | 3.128921 | 1.519521 |
| 69. | 1. | 0. | -4.264122 | 1.809460 | 2.623569 |
| 70. | 1. | 0. | 1.855014 | -3.587347 | 2.299918 |
| 71. | 1. | 0. | 1.608724 | -3.780072 | 0.563884 |
| 72. | 1. | 0. | 3.165288 | -3.226825 | 1.174848 |
| 73. | 1. | 0. | 0.808718 | -0.796125 | 4.278372 |
| 74. | 1. | 0. | 1.164726 | -2.466126 | 3.811719 |
| 75. | 1. | 0. | -0.468308 | -1.815657 | 3.621386 |
| 76. | 1. | 0. | 1.973659 | 1.272655 | 2.610181 |

| **3R-3** | | Standard Orientation  (Ångstroms) | | | |
| --- | --- | --- | --- | --- | --- |
| Center number | Atom number | Type | X | Y | Z |
| 1. | 6. | 0. | -1.151307 | -1.502505 | 2.185681 |
| 2. | 6. | 0. | -1.415269 | -1.484340 | 0.690106 |
| 3. | 6. | 0. | -0.145214 | -1.036586 | -0.099001 |
| 4. | 6. | 0. | 0.863489 | -2.212079 | 0.073722 |
| 5. | 6. | 0. | 1.139125 | -2.399935 | 1.605408 |
| 6. | 6. | 0. | 0.053859 | -1.955760 | 2.586658 |
| 7. | 6. | 0. | 2.203770 | -2.280994 | -0.647913 |
| 8. | 6. | 0. | 0.379054 | 0.334339 | 0.392396 |
| 9. | 6. | 0. | 1.727843 | 0.628591 | 0.433699 |
| 10. | 6. | 0. | -7.541804 | 2.571498 | -0.089053 |
| 11. | 6. | 0. | -7.566240 | 1.805369 | -1.247978 |
| 12. | 6. | 0. | -6.679341 | 0.726447 | -1.326683 |
| 13. | 6. | 0. | -5.776552 | 0.405329 | -0.276046 |
| 14. | 6. | 0. | -5.778616 | 1.199938 | 0.884166 |
| 15. | 6. | 0. | -6.657878 | 2.271306 | 0.967581 |
| 16. | 7. | 0. | -6.481840 | -0.198517 | -2.331394 |
| 17. | 6. | 0. | -5.493399 | -1.084614 | -1.945888 |
| 18. | 6. | 0. | -5.029087 | -0.758257 | -0.694020 |
| 19. | 6. | 0. | -3.944019 | -1.463059 | 0.070627 |
| 20. | 6. | 0. | -0.764828 | -0.755004 | -1.505264 |
| 21. | 7. | 0. | -2.098263 | -0.576561 | -1.309446 |
| 22. | 6. | 0. | -2.596309 | -0.710137 | 0.057754 |
| 23. | 1. | 0. | -1.593732 | -2.523505 | 0.373673 |
| 24. | 8. | 0. | -0.192945 | -0.655357 | -2.580823 |
| 25. | 6. | 0. | 2.580427 | -1.748138 | -1.814435 |
| 26. | 6. | 0. | 3.982320 | -1.832060 | -2.359645 |
| 27. | 6. | 0. | 4.588848 | -0.428132 | -2.663685 |
| 28. | 6. | 0. | 4.497681 | 0.517601 | -1.472237 |
| 29. | 6. | 0. | 5.110568 | 0.469319 | -0.279962 |
| 30. | 6. | 0. | 4.770497 | 1.485466 | 0.807824 |
| 31. | 6. | 0. | 3.543159 | 2.345957 | 0.678119 |
| 32. | 6. | 0. | 2.235227 | 1.943779 | 0.601191 |
| 33. | 6. | 0. | 5.991219 | -0.533463 | -3.291262 |
| 34. | 6. | 0. | 6.143271 | -0.535972 | 0.164305 |
| 35. | 6. | 0. | -2.239810 | -1.020599 | 3.108444 |
| 36. | 6. | 0. | 0.514423 | -2.054458 | 4.022056 |
| 37. | 8. | 0. | 1.538566 | -3.756446 | 1.857701 |
| 38. | 7. | 0. | 3.940852 | 3.677412 | 0.713570 |
| 39. | 7. | 0. | 1.260228 | 2.983755 | 0.585707 |
| 40. | 8. | 0. | 5.801201 | 2.512473 | 0.905110 |
| 41. | 8. | 0. | -0.543424 | 1.211050 | 0.666534 |
| 42. | 7. | 0. | 5.182533 | 3.775790 | 0.834817 |
| 43. | 7. | 0. | 0.095659 | 2.950084 | 0.613533 |
| 44. | 1. | 0. | 0.299239 | -3.105577 | -0.236200 |
| 45. | 1. | 0. | 2.040923 | -1.827756 | 1.855497 |
| 46. | 1. | 0. | 2.929014 | -2.879178 | -0.094306 |
| 47. | 1. | 0. | 2.433510 | -0.125896 | 0.132554 |
| 48. | 1. | 0. | -8.216711 | 3.416899 | 0.004695 |
| 49. | 1. | 0. | -8.248540 | 2.036746 | -2.060927 |
| 50. | 1. | 0. | -5.103199 | 0.979621 | 1.706004 |
| 51. | 1. | 0. | -6.666604 | 2.891314 | 1.858866 |
| 52. | 1. | 0. | -6.990077 | -0.236319 | -3.199392 |
| 53. | 1. | 0. | -5.204080 | -1.900851 | -2.593460 |
| 54. | 1. | 0. | -3.786100 | -2.464025 | -0.349343 |
| 55. | 1. | 0. | -4.257508 | -1.601192 | 1.111756 |
| 56. | 1. | 0. | -2.683485 | -0.242381 | -2.062674 |
| 57. | 1. | 0. | -2.720816 | 0.279029 | 0.507864 |
| 58. | 1. | 0. | 1.862787 | -1.174323 | -2.396955 |
| 59. | 1. | 0. | 3.984667 | -2.393875 | -3.305372 |
| 60. | 1. | 0. | 4.627482 | -2.382455 | -1.664448 |
| 61. | 1. | 0. | 3.941372 | 0.012905 | -3.432350 |
| 62. | 1. | 0. | 3.781728 | 1.325902 | -1.608427 |
| 63. | 1. | 0. | 4.761099 | 0.957966 | 1.775403 |
| 64. | 1. | 0. | 5.945718 | -1.109138 | -4.222097 |
| 65. | 1. | 0. | 6.709100 | -1.029131 | -2.632184 |
| 66. | 1. | 0. | 6.388790 | 0.458212 | -3.527019 |
| 67. | 1. | 0. | 7.084663 | -0.030631 | 0.412951 |
| 68. | 1. | 0. | 6.353545 | -1.290749 | -0.591528 |
| 69. | 1. | 0. | 5.814017 | -1.053867 | 1.074505 |
| 70. | 1. | 0. | -1.934633 | -1.014963 | 4.154690 |
| 71. | 1. | 0. | -2.534638 | 0.002013 | 2.846314 |
| 72. | 1. | 0. | -3.139105 | -1.644428 | 3.029970 |
| 73. | 1. | 0. | 1.032713 | -3.004896 | 4.178619 |
| 74. | 1. | 0. | -0.297833 | -1.984590 | 4.746182 |
| 75. | 1. | 0. | 1.236236 | -1.258691 | 4.251433 |
| 76. | 1. | 0. | 0.758456 | -4.312778 | 1.720019 |

| **3R -4** | | Standard Orientation  (Ångstroms) | | | |
| --- | --- | --- | --- | --- | --- |
| Center number | Atom number | Type | X | Y | Z |
| 1. | 6. | 0. | 0.897782 | 2.774550 | -0.866215 |
| 2. | 6. | 0. | 1.426797 | 1.352928 | -0.919347 |
| 3. | 6. | 0. | 0.271139 | 0.324175 | -0.712145 |
| 4. | 6. | 0. | -0.572789 | 0.413291 | -2.018006 |
| 5. | 6. | 0. | -1.028811 | 1.900234 | -2.233163 |
| 6. | 6. | 0. | -0.258212 | 3.005411 | -1.517748 |
| 7. | 6. | 0. | -1.769839 | -0.496378 | -2.278954 |
| 8. | 6. | 0. | -0.514354 | 0.601755 | 0.586216 |
| 9. | 6. | 0. | -1.881699 | 0.468968 | 0.676604 |
| 10. | 6. | 0. | 7.253649 | -3.044078 | 0.299076 |
| 11. | 6. | 0. | 7.493902 | -2.003755 | 1.188440 |
| 12. | 6. | 0. | 6.717037 | -0.848212 | 1.056383 |
| 13. | 6. | 0. | 5.710690 | -0.715752 | 0.058730 |
| 14. | 6. | 0. | 5.496704 | -1.787000 | -0.829639 |
| 15. | 6. | 0. | 6.265789 | -2.936352 | -0.700865 |
| 16. | 7. | 0. | 6.732760 | 0.327526 | 1.776663 |
| 17. | 6. | 0. | 5.779988 | 1.187475 | 1.265686 |
| 18. | 6. | 0. | 5.123826 | 0.596354 | 0.213522 |
| 19. | 6. | 0. | 3.997861 | 1.193026 | -0.585955 |
| 20. | 6. | 0. | 1.090303 | -0.977570 | -0.435289 |
| 21. | 7. | 0. | 2.338486 | -0.582434 | -0.070072 |
| 22. | 6. | 0. | 2.594903 | 0.857457 | -0.036276 |
| 23. | 1. | 0. | 1.782281 | 1.175599 | -1.945031 |
| 24. | 8. | 0. | 0.703586 | -2.136180 | -0.483308 |
| 25. | 6. | 0. | -1.991022 | -1.759978 | -1.903702 |
| 26. | 6. | 0. | -3.268493 | -2.507270 | -2.181426 |
| 27. | 6. | 0. | -3.978587 | -2.990084 | -0.879635 |
| 28. | 6. | 0. | -4.221078 | -1.853214 | 0.105286 |
| 29. | 6. | 0. | -5.037022 | -0.791882 | 0.019512 |
| 30. | 6. | 0. | -5.025066 | 0.285265 | 1.101702 |
| 31. | 6. | 0. | -3.923007 | 0.311955 | 2.126273 |
| 32. | 6. | 0. | -2.577253 | 0.464277 | 1.916869 |
| 33. | 6. | 0. | -5.220752 | -3.845848 | -1.190922 |
| 34. | 6. | 0. | -6.016452 | -0.492806 | -1.087916 |
| 35. | 6. | 0. | 1.686502 | 3.810538 | -0.109603 |
| 36. | 6. | 0. | -0.948373 | 4.341689 | -1.635453 |
| 37. | 8. | 0. | -0.998089 | 2.218002 | -3.634682 |
| 38. | 7. | 0. | -4.502169 | 0.214428 | 3.386849 |
| 39. | 7. | 0. | -1.766101 | 0.538682 | 3.081854 |
| 40. | 8. | 0. | -6.189260 | 0.163795 | 1.969176 |
| 41. | 8. | 0. | 0.236424 | 0.886926 | 1.620271 |
| 42. | 7. | 0. | -5.747132 | 0.127691 | 3.308848 |
| 43. | 7. | 0. | -0.613054 | 0.713918 | 3.196595 |
| 44. | 1. | 0. | 0.149076 | 0.230537 | -2.825492 |
| 45. | 1. | 0. | -2.069886 | 1.982534 | -1.879572 |
| 46. | 1. | 0. | -2.553927 | -0.014547 | -2.871806 |
| 47. | 1. | 0. | -2.435958 | 0.173113 | -0.196403 |
| 48. | 1. | 0. | 7.838323 | -3.955804 | 0.375316 |
| 49. | 1. | 0. | 8.256695 | -2.086265 | 1.957453 |
| 50. | 1. | 0. | 4.740370 | -1.717302 | -1.606140 |
| 51. | 1. | 0. | 6.105741 | -3.767418 | -1.380911 |
| 52. | 1. | 0. | 7.351709 | 0.535155 | 2.542820 |
| 53. | 1. | 0. | 5.644024 | 2.170859 | 1.694148 |
| 54. | 1. | 0. | 4.055944 | 0.853180 | -1.627709 |
| 55. | 1. | 0. | 4.104065 | 2.282713 | -0.605282 |
| 56. | 1. | 0. | 2.998135 | -1.261564 | 0.285663 |
| 57. | 1. | 0. | 2.515161 | 1.224638 | 0.991601 |
| 58. | 1. | 0. | -1.235528 | -2.276933 | -1.315378 |
| 59. | 1. | 0. | -3.051685 | -3.403247 | -2.781054 |
| 60. | 1. | 0. | -3.951514 | -1.888041 | -2.775941 |
| 61. | 1. | 0. | -3.263234 | -3.657339 | -0.382306 |
| 62. | 1. | 0. | -3.589166 | -1.898680 | 0.989800 |
| 63. | 1. | 0. | -5.103846 | 1.266631 | 0.605833 |
| 64. | 1. | 0. | -4.935804 | -4.724661 | -1.779343 |
| 65. | 1. | 0. | -5.975475 | -3.297773 | -1.761372 |
| 66. | 1. | 0. | -5.693293 | -4.196134 | -0.268491 |
| 67. | 1. | 0. | -7.041676 | -0.483984 | -0.697880 |
| 68. | 1. | 0. | -5.969869 | -1.213995 | -1.902069 |
| 69. | 1. | 0. | -5.833419 | 0.504384 | -1.509004 |
| 70. | 1. | 0. | 1.170915 | 4.768823 | -0.046592 |
| 71. | 1. | 0. | 1.880137 | 3.471445 | 0.914215 |
| 72. | 1. | 0. | 2.661808 | 3.993584 | -0.578268 |
| 73. | 1. | 0. | -1.151926 | 4.556235 | -2.689277 |
| 74. | 1. | 0. | -0.369030 | 5.169141 | -1.225533 |
| 75. | 1. | 0. | -1.918658 | 4.323904 | -1.120398 |
| 76. | 1. | 0. | -1.675687 | 1.687174 | -4.074641 |

| **3R-5** | | Standard Orientation  (Ångstroms) | | | |
| --- | --- | --- | --- | --- | --- |
| Center number | Atom number | Type | X | Y | Z |
| 1. | 6. | 0. | -1.143377 | -1.455679 | 2.211429 |
| 2. | 6. | 0. | -1.412484 | -1.461529 | 0.717819 |
| 3. | 6. | 0. | -0.142290 | -1.029492 | -0.079394 |
| 4. | 6. | 0. | 0.853435 | -2.215355 | 0.091888 |
| 5. | 6. | 0. | 1.089493 | -2.463347 | 1.622701 |
| 6. | 6. | 0. | 0.041292 | -1.958281 | 2.609024 |
| 7. | 6. | 0. | 2.209133 | -2.250369 | -0.609492 |
| 8. | 6. | 0. | 0.394435 | 0.338642 | 0.394769 |
| 9. | 6. | 0. | 1.741773 | 0.623267 | 0.461226 |
| 10. | 6. | 0. | -7.516430 | 2.603100 | -0.147613 |
| 11. | 6. | 0. | -7.553024 | 1.804030 | -1.283782 |
| 12. | 6. | 0. | -6.672439 | 0.718435 | -1.337463 |
| 13. | 6. | 0. | -5.764044 | 0.422649 | -0.284121 |
| 14. | 6. | 0. | -5.753922 | 1.250259 | 0.852708 |
| 15. | 6. | 0. | -6.626801 | 2.328519 | 0.911175 |
| 16. | 7. | 0. | -6.486525 | -0.235843 | -2.316641 |
| 17. | 6. | 0. | -5.500118 | -1.115947 | -1.912347 |
| 18. | 6. | 0. | -5.025482 | -0.756548 | -0.673543 |
| 19. | 6. | 0. | -3.939571 | -1.446743 | 0.103092 |
| 20. | 6. | 0. | -0.763824 | -0.764653 | -1.488918 |
| 21. | 7. | 0. | -2.095364 | -0.580296 | -1.294727 |
| 22. | 6. | 0. | -2.591853 | -0.694893 | 0.075211 |
| 23. | 1. | 0. | -1.589044 | -2.506689 | 0.423431 |
| 24. | 8. | 0. | -0.191316 | -0.685503 | -2.566584 |
| 25. | 6. | 0. | 2.564055 | -1.813415 | -1.821952 |
| 26. | 6. | 0. | 3.973888 | -1.854945 | -2.349835 |
| 27. | 6. | 0. | 4.535424 | -0.437015 | -2.676461 |
| 28. | 6. | 0. | 4.460284 | 0.506028 | -1.481941 |
| 29. | 6. | 0. | 5.117040 | 0.474529 | -0.312599 |
| 30. | 6. | 0. | 4.788326 | 1.477099 | 0.791075 |
| 31. | 6. | 0. | 3.559261 | 2.339134 | 0.686994 |
| 32. | 6. | 0. | 2.249557 | 1.940400 | 0.622560 |
| 33. | 6. | 0. | 5.919059 | -0.510798 | -3.349146 |
| 34. | 6. | 0. | 6.196687 | -0.498305 | 0.091321 |
| 35. | 6. | 0. | -2.205609 | -0.910098 | 3.128670 |
| 36. | 6. | 0. | 0.502875 | -2.091153 | 4.039095 |
| 37. | 8. | 0. | 1.241633 | -3.871586 | 1.866180 |
| 38. | 7. | 0. | 3.959392 | 3.670036 | 0.731110 |
| 39. | 7. | 0. | 1.277891 | 2.980324 | 0.623264 |
| 40. | 8. | 0. | 5.821021 | 2.500540 | 0.887375 |
| 41. | 8. | 0. | -0.527838 | 1.231679 | 0.633794 |
| 42. | 7. | 0. | 5.201658 | 3.767347 | 0.836846 |
| 43. | 7. | 0. | 0.109559 | 2.935976 | 0.638132 |
| 44. | 1. | 0. | 0.294250 | -3.097733 | -0.247190 |
| 45. | 1. | 0. | 2.029754 | -1.957331 | 1.897838 |
| 46. | 1. | 0. | 2.995613 | -2.712965 | -0.004674 |
| 47. | 1. | 0. | 2.448576 | -0.141073 | 0.190694 |
| 48. | 1. | 0. | -8.186235 | 3.454517 | -0.073653 |
| 49. | 1. | 0. | -8.239797 | 2.015585 | -2.098383 |
| 50. | 1. | 0. | -5.074331 | 1.049602 | 1.676115 |
| 51. | 1. | 0. | -6.626131 | 2.973812 | 1.784378 |
| 52. | 1. | 0. | -7.002125 | -0.296877 | -3.178939 |
| 53. | 1. | 0. | -5.219581 | -1.952236 | -2.537817 |
| 54. | 1. | 0. | -3.782718 | -2.455808 | -0.296980 |
| 55. | 1. | 0. | -4.250219 | -1.564864 | 1.147670 |
| 56. | 1. | 0. | -2.683269 | -0.264470 | -2.053837 |
| 57. | 1. | 0. | -2.715960 | 0.301243 | 0.509954 |
| 58. | 1. | 0. | 1.815091 | -1.341575 | -2.454833 |
| 59. | 1. | 0. | 4.003209 | -2.436311 | -3.282787 |
| 60. | 1. | 0. | 4.633658 | -2.366419 | -1.638304 |
| 61. | 1. | 0. | 3.853509 | -0.013424 | -3.424702 |
| 62. | 1. | 0. | 3.717617 | 1.293562 | -1.591476 |
| 63. | 1. | 0. | 4.790805 | 0.936859 | 1.751977 |
| 64. | 1. | 0. | 5.854585 | -1.079166 | -4.283275 |
| 65. | 1. | 0. | 6.667846 | -0.997700 | -2.718511 |
| 66. | 1. | 0. | 6.289297 | 0.490454 | -3.588510 |
| 67. | 1. | 0. | 7.133360 | 0.035919 | 0.292261 |
| 68. | 1. | 0. | 6.393967 | -1.254074 | -0.667107 |
| 69. | 1. | 0. | 5.925553 | -1.015286 | 1.021167 |
| 70. | 1. | 0. | -1.889321 | -0.879395 | 4.171245 |
| 71. | 1. | 0. | -2.476063 | 0.110627 | 2.835071 |
| 72. | 1. | 0. | -3.121644 | -1.512469 | 3.081887 |
| 73. | 1. | 0. | 0.799626 | -3.126483 | 4.234089 |
| 74. | 1. | 0. | -0.255628 | -1.810038 | 4.769575 |
| 75. | 1. | 0. | 1.389065 | -1.467416 | 4.219256 |
| 76. | 1. | 0. | 2.053930 | -4.161413 | 1.429463 |

**
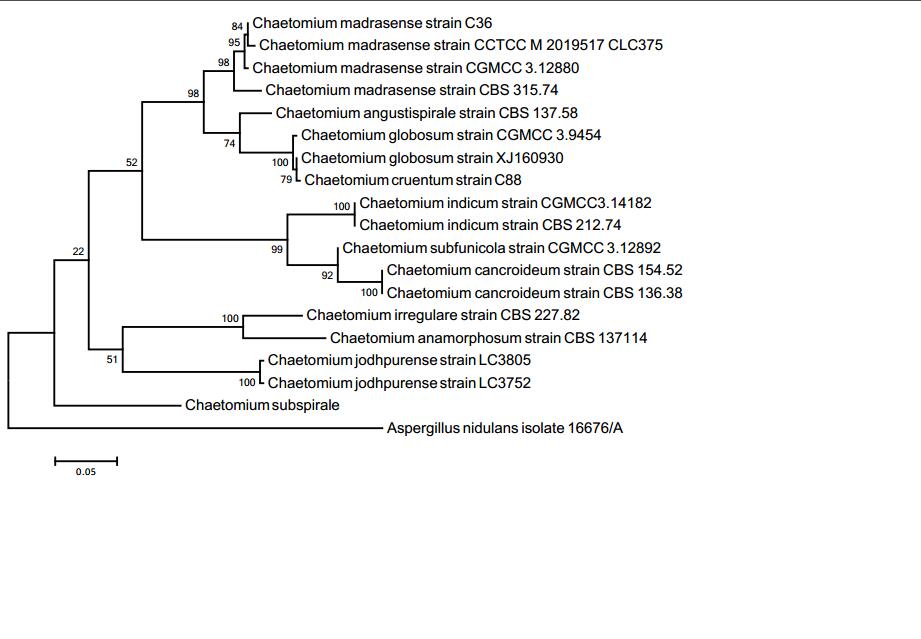
**

**Figure S32.** Molecular phylogenetic analysis of beta-Tubulin gene from different species

Partial beta-tubulin gene from *Chaetomium madrasense* 375 was amplified with primers Bt2a (5’GGTAACCAAATCGGTGCTGCTTTC 3’) and Bt2b (5’ACCCTCAGTGTAGTGACCCTTGGC 3’) [1], which was used to conduct the phylogenetic analysis. The molecular phylogenetic analysis was inferred by using the Maximum Likelihood method with the software MEGA7. The fungus was identified as *Chaetomium madrasense* according to its internal transcribed spacer (ITS) sequence of ITS rDNA and beta-tubulin encoding gene from genomic DNA, as well as its morphological features. A phylogenetic tree was constructed based on the sequence of partial beta-tubulin gene from *C. madrasense* and other species in the genus *Chaetomium*, with Aspergillus nidulans as the outgroup. The results demonstrated this strain was clustered with three other strains belonging to *C. madrasense.*

CGGTGCTGCTTTCTGGTATGTTCAAGCAAAGCAAACGCCCGTGGTTGATGGCAATCGAGACTAACTTGTTCTTCAGGCAGACCATCTCCGGCGAGCACGGCCTCGACGGCAATGGCGTGTATGTGGCTATGGACAATCCCCCGACCGATAAACCCCCGCTCACCGCTTCGATAGGTACAACGGCACCTCTGAGCTCCACCTCGAGCGCATGAACGTCTACTTCAACCAGGTCAGTTGGGATGAATACCTAGGTATATACATTCAAGGGAAAGCGTGCTGACAGTATCATACAGGCTTCCGGTAACAAGTATGTTCCCCGTGCCGTCCTCGTCGACTTGGAGCCCGGCACCATGGATGCCGTCCGTGCCGGCCCCTTCGGCCAGCTCTTCCGCCCGGACAACT

**Figure S33.** beta-tubulin encoding gene sequence of *C. madrasense* 375

TTCCGTAGGGGGACCTGCGGAGGGATCATTACAGAGTTGCAAAACTCCCTAAACCATTGTGAACGTTACCTATACCGTTGCTTCGGCGGGCGGCCCCGGGGTTTACCCCCCGGGCGCCCCTGGGCCCCACCGCGGGCGCCCGCCGGAGGTCACCAAACTCTTGATAATTTATGGCCTCTCTGAGTCTTCTGTACTGAATAAGTCAAAACTTTCAACAACGGATCTCTTGGTTCTGGCATCGATGAAGAACGCAGCGAAATGCGATAAGTAATGTGAATTGCAGAATTCAGTGAATCATCGAATCTTTGAACGCACATTGCGCCCGCCAGCATTCTGGCGGGCATGCCTGTTCGAGCGTCATTTCAACCATCAAGCCCCCGGGCTTGTGTTGGGGACCTGCGGCTGCCGCAGGCCCTGAAAAGCAGTGGCGGGCTCGCTGTCGCACCGAGCGTAGTAGCATACATCTCGCTCTGGTCGCGCCGCGGGTTCCGGCCGTTAAACCACCTTTTAACCCAAGGTTGACCTCGGATCAGGTAGGAAGACCCGCTGAACTTAAGCATATCAATAAGCGGAGGAA

**Figure S34.** 18S rDNA gene sequence of 375

**
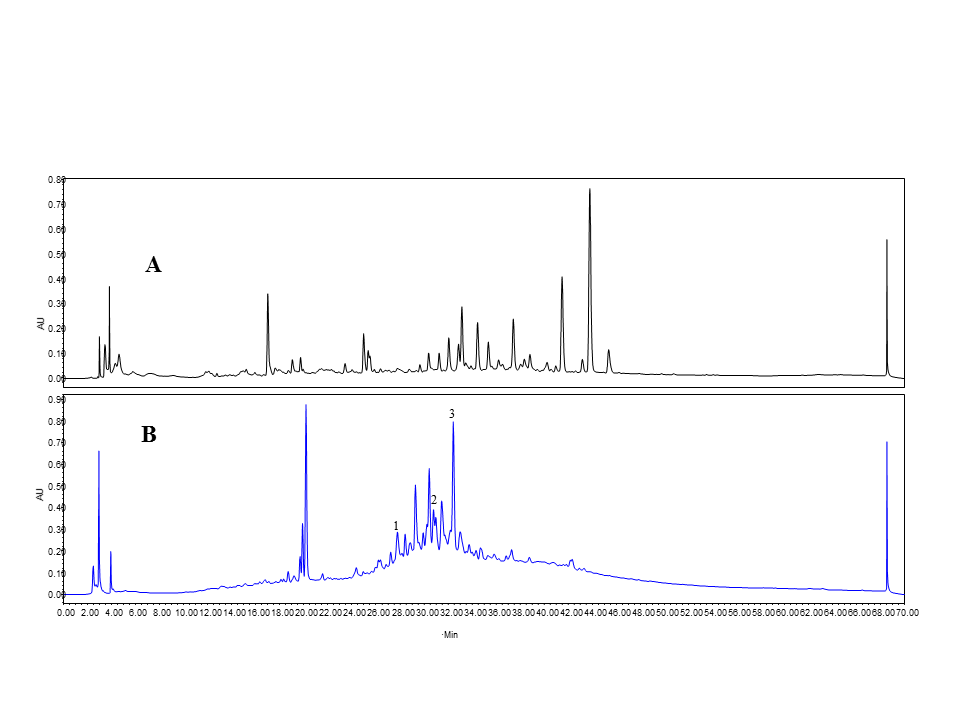
**

**Figure S35.** HPLC chromatogram analysis of the crude extracts (A) from 375 and modified production (B)

**
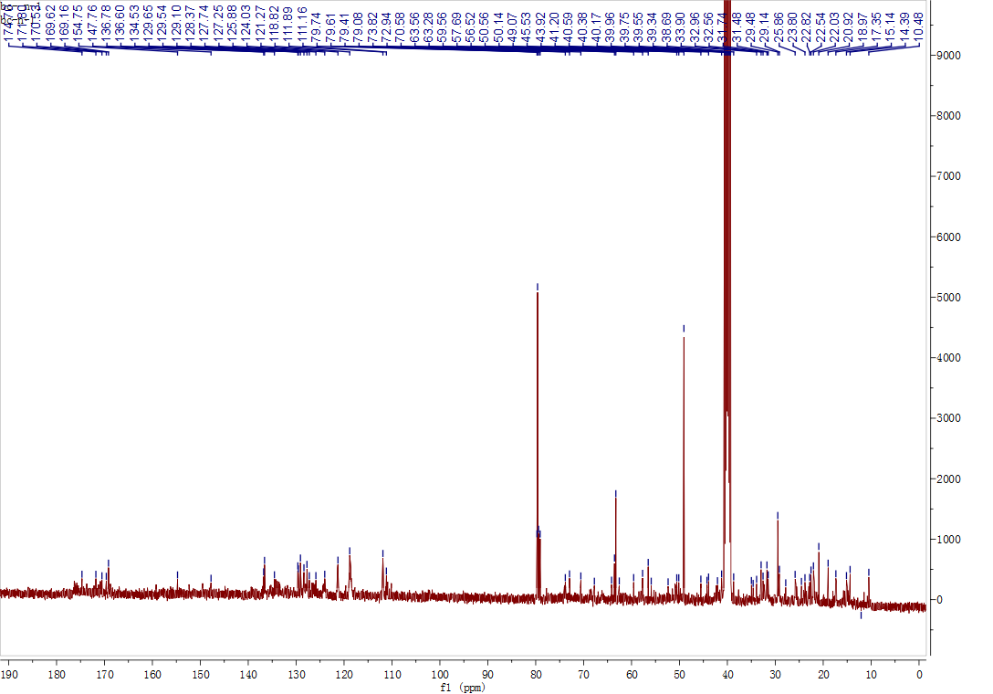
**

**Figure S36.** ^13^C NMR spectrum of modified production in DMSO-*d*_6_ (100 MHz)

**Figure S37.** Four possible structures of compound **3**


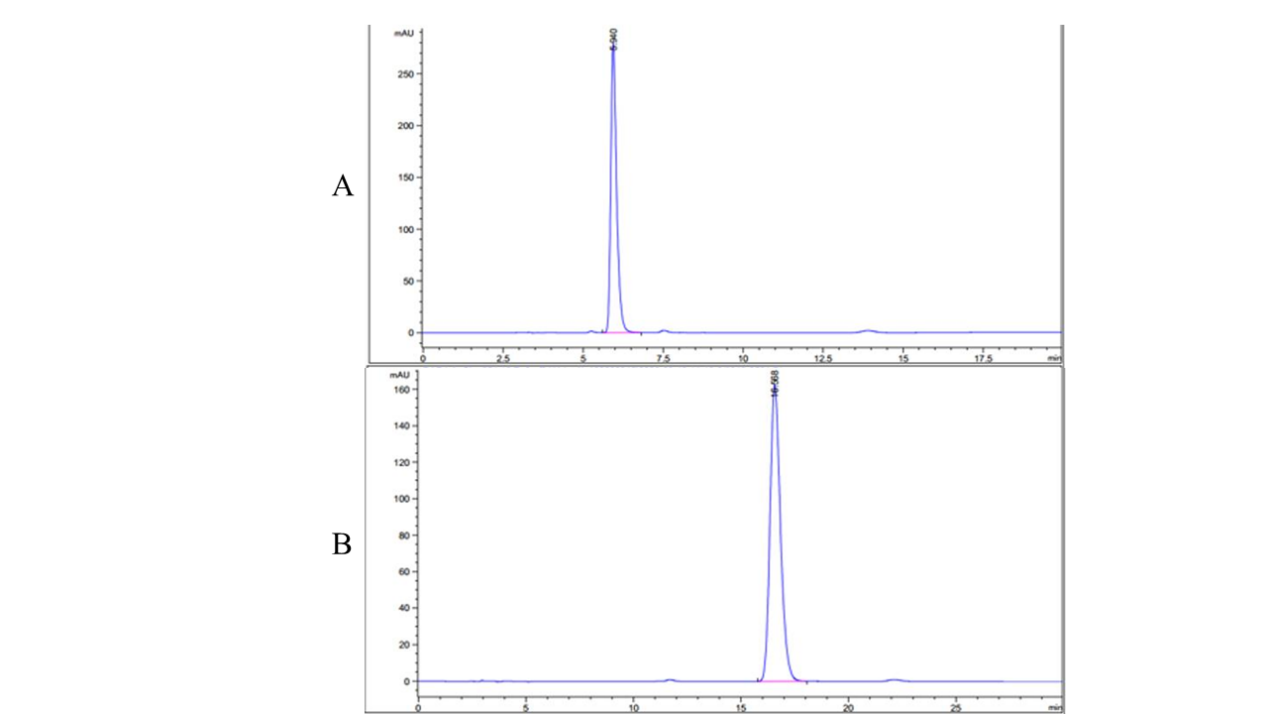


**Figure S38.** Chiral HPLC analysis of compound **3**

(HPLC analysis of compound **3** was carry out by Agilent 1200 (Agilent Technologies, USA) on a chiral column Daicel Chiralcel OD-H column (Daicel Chemical Industries, Ltd., Japan). The mobile phase consisted of isopropyl and hexane (A: 60:40; B: 80:20) with a flow rate of 0.5 mL/min. The detection wavelength was at 254 nm.)


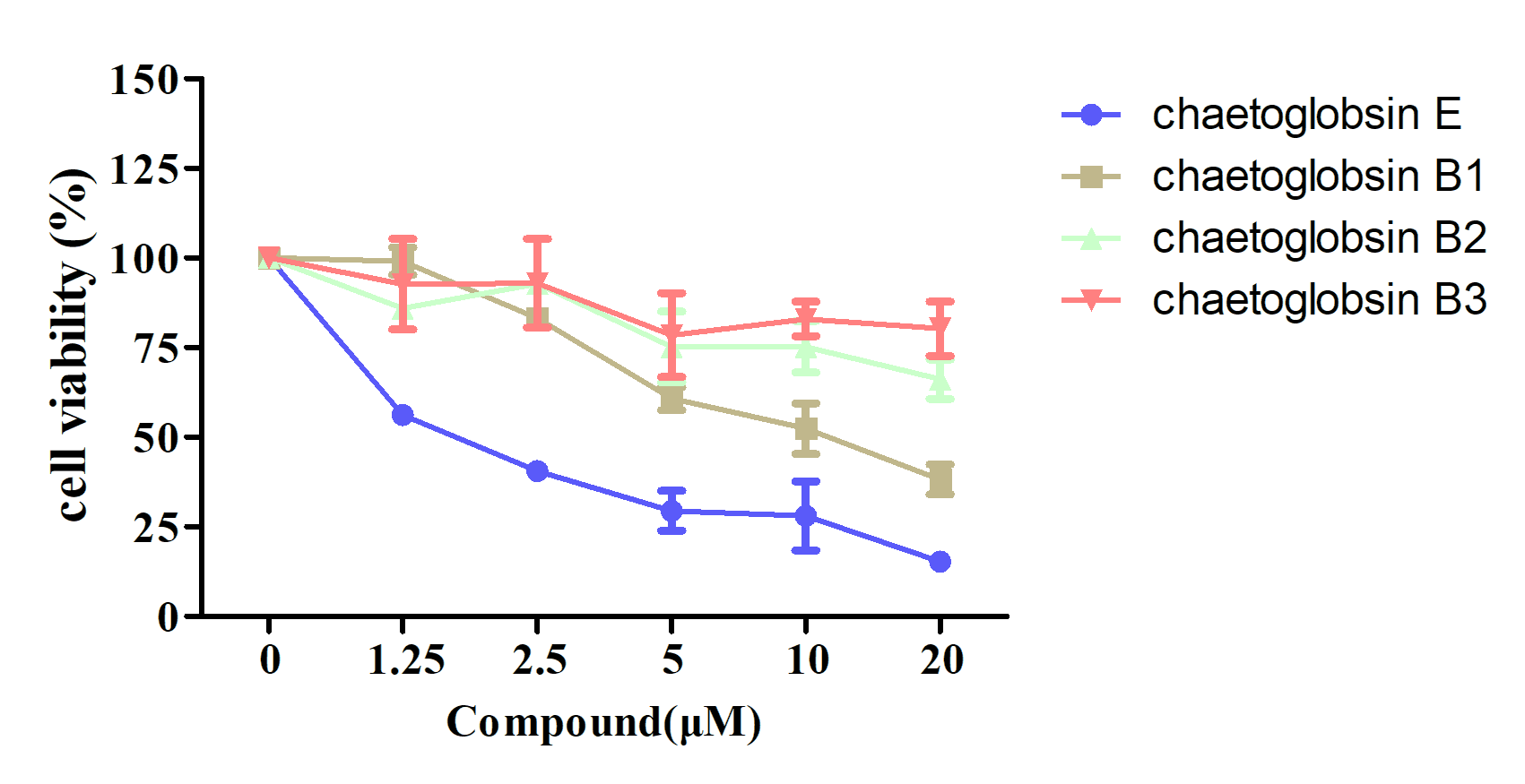

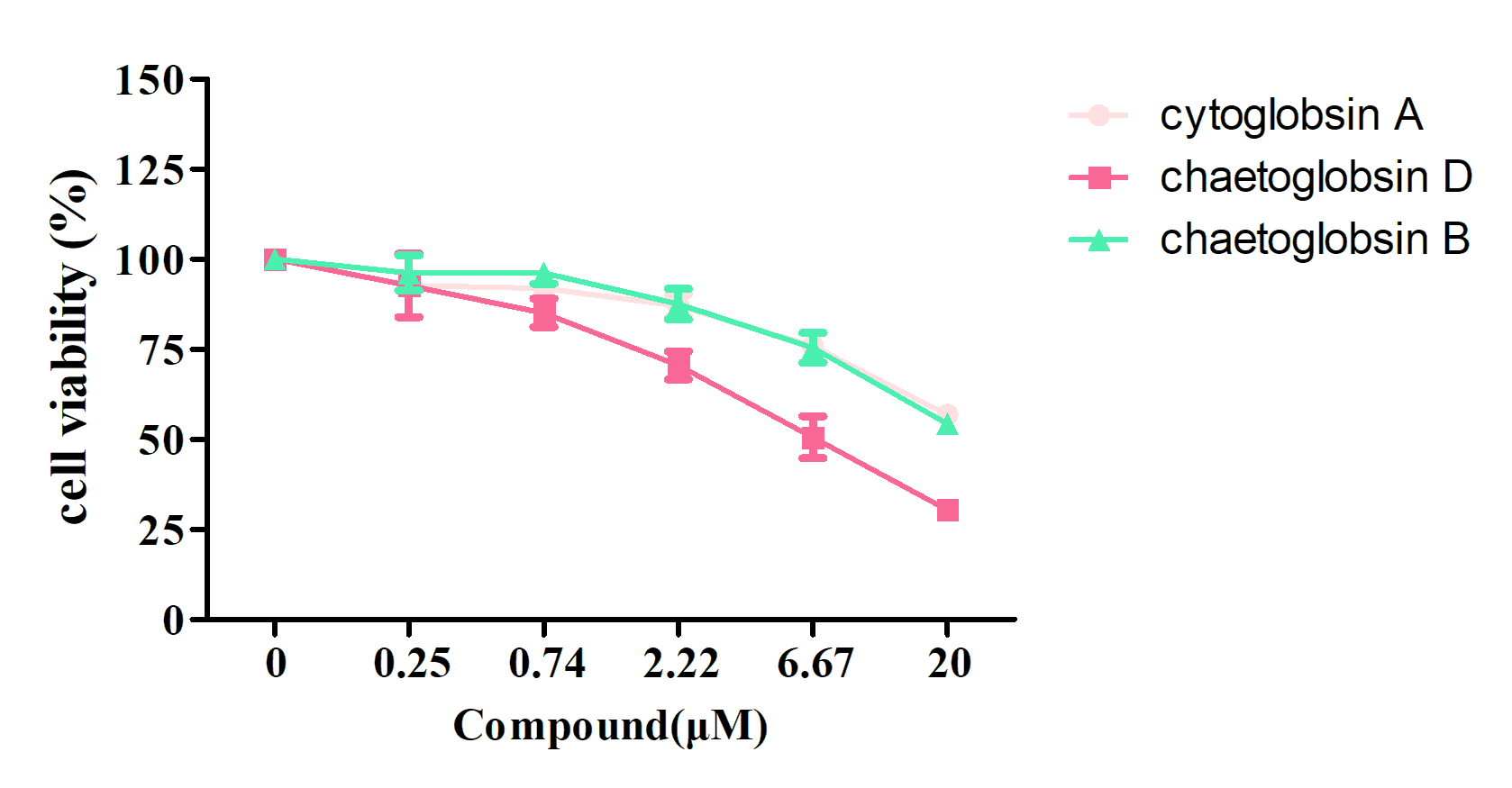


**Figure S39.** IC_50_ curve of compounds **1**-**7** against A549 cell lines


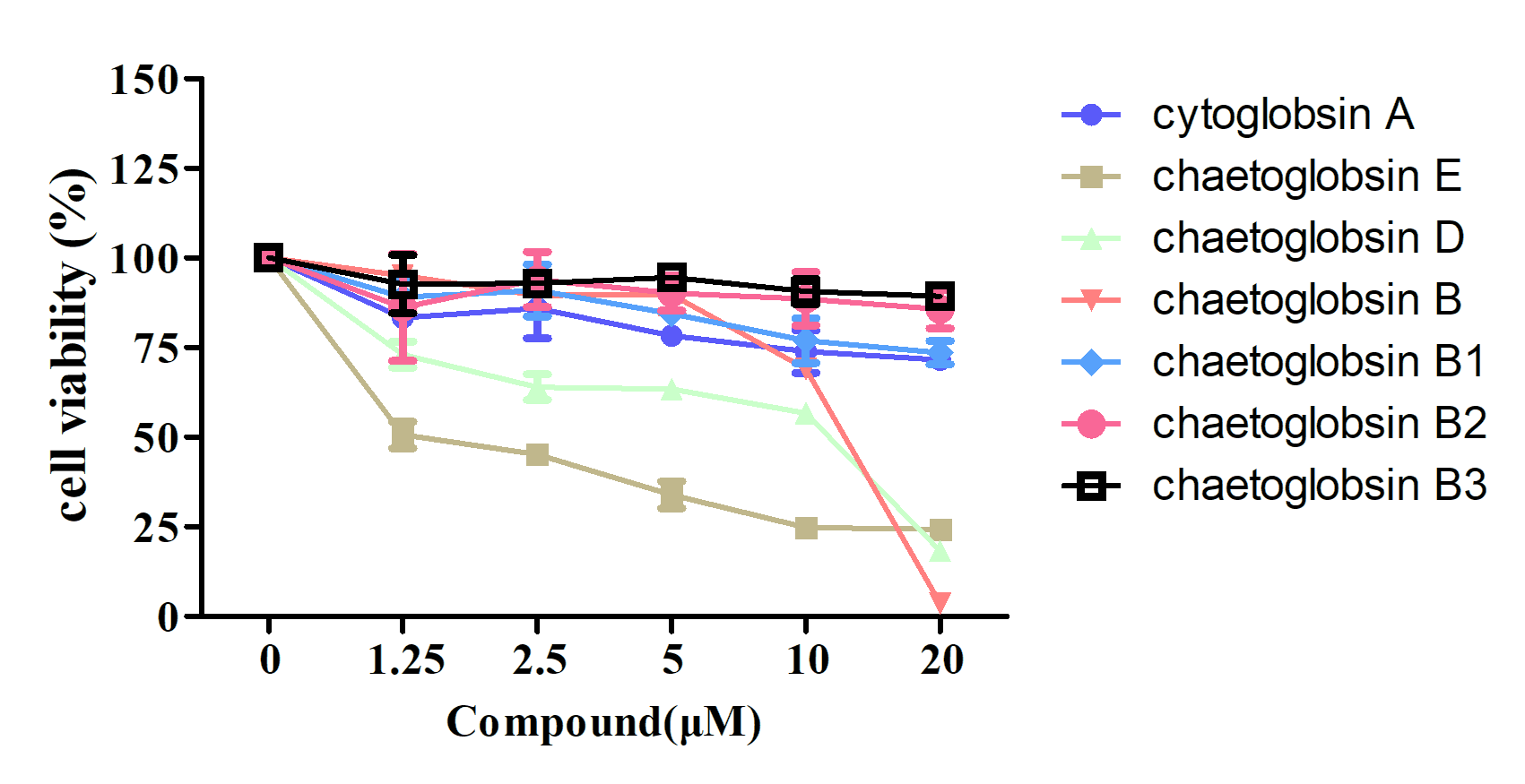


**Figure S40.** IC_50_ curve of compounds **1**-**7** against HCC827 cell lines


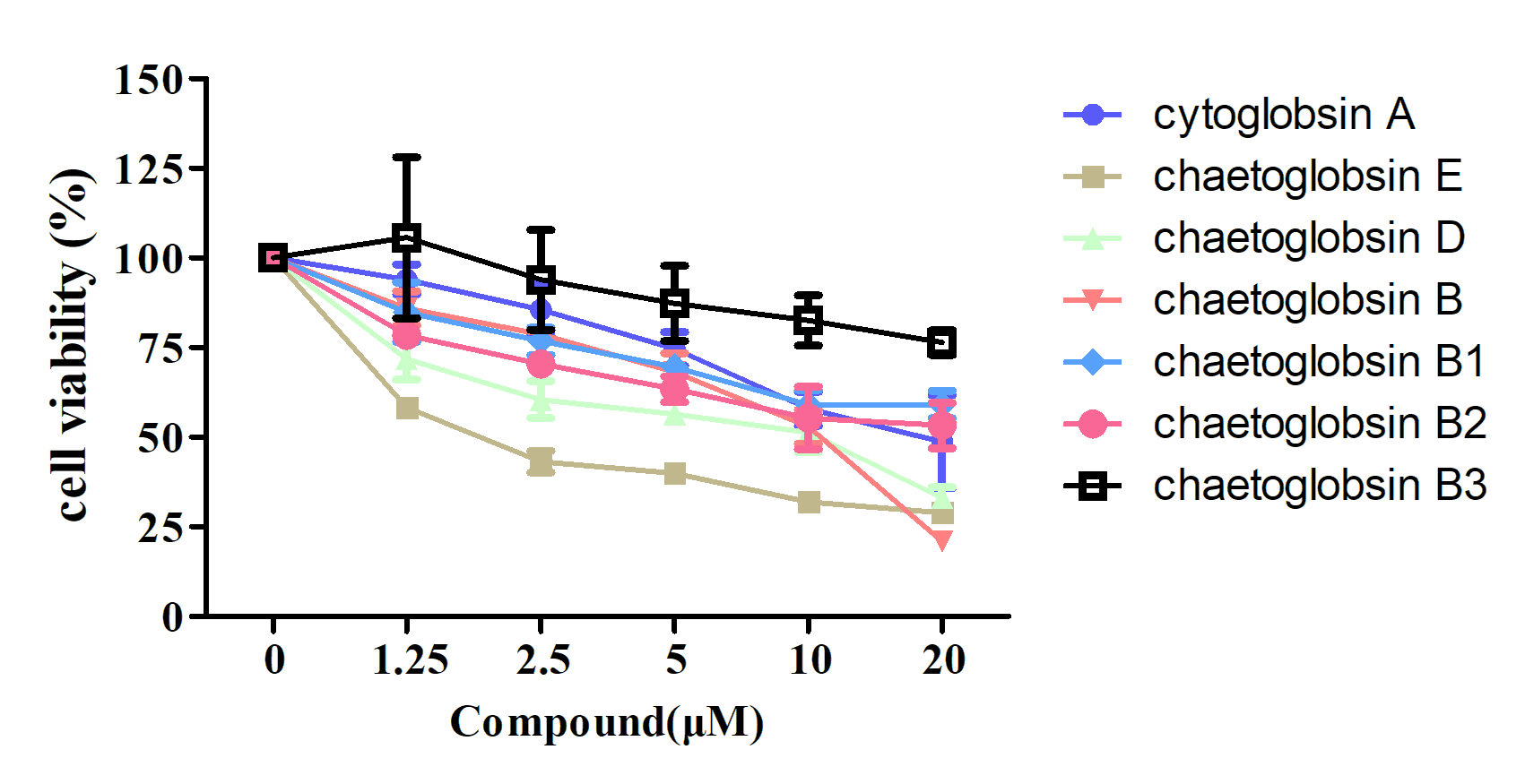


**Figure S41.** IC_50_ curve of compounds **1**-**7** against SW620 cell lines


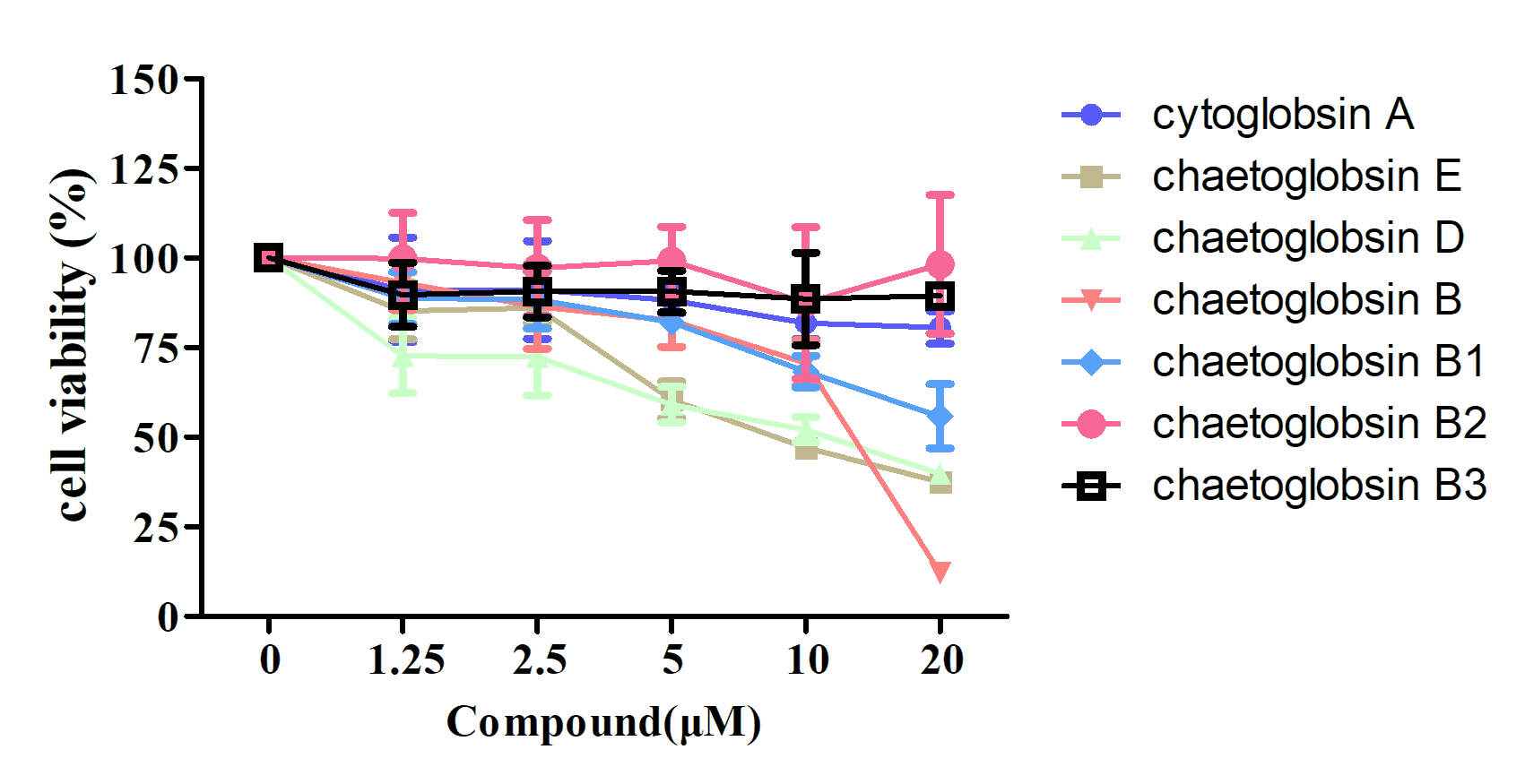


**Figure S42.** IC_50_ curve of compounds **1**-**7** against MDA-MB-231 cell lines

**A**

**B**


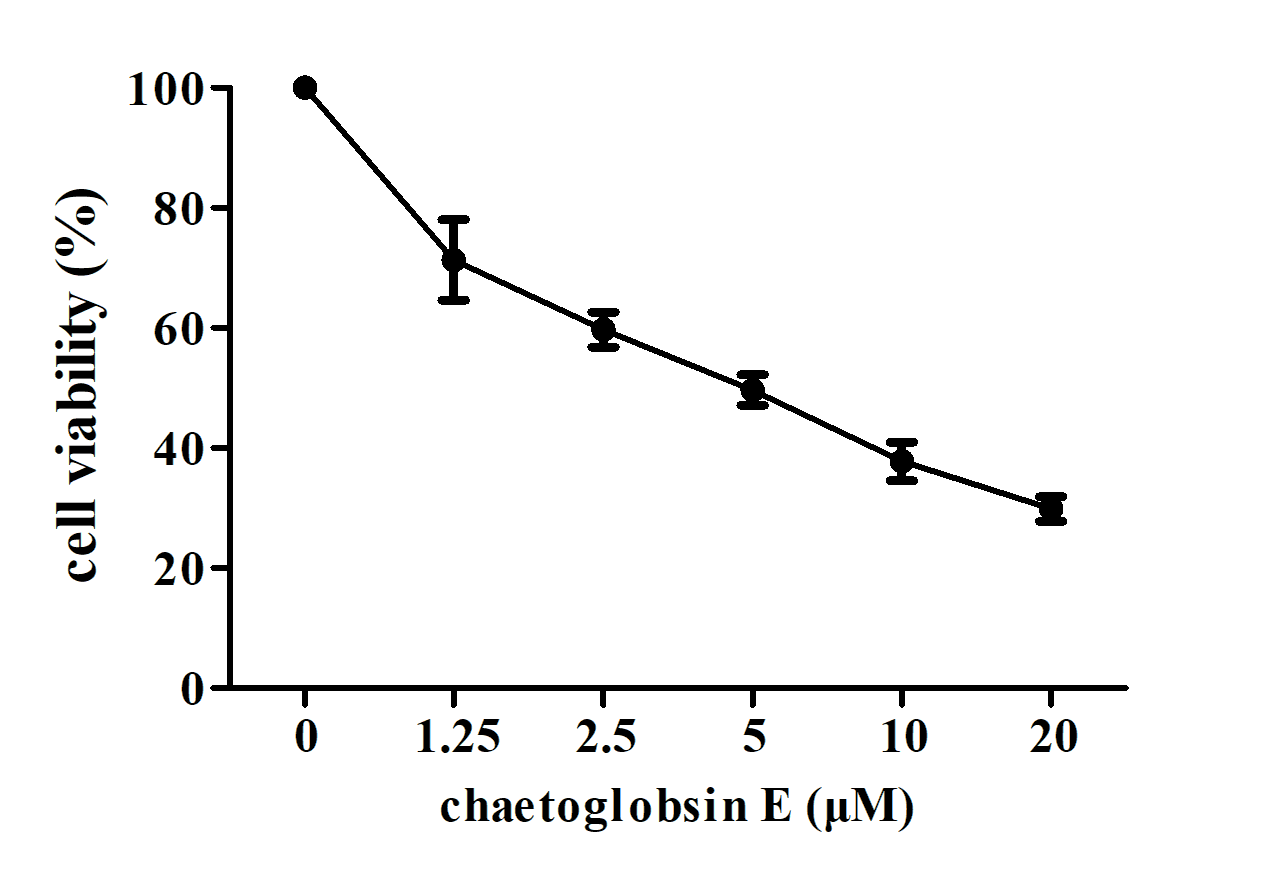

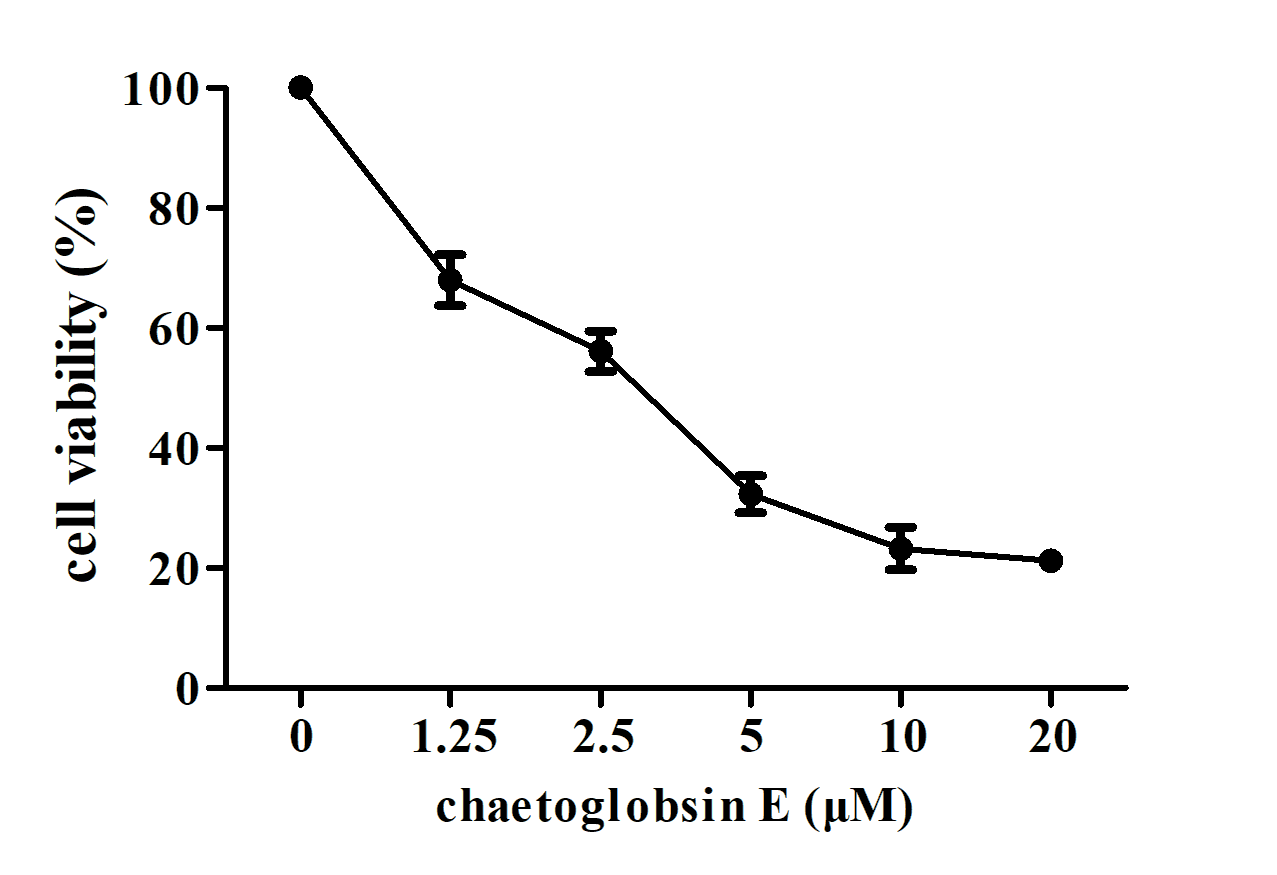


**Figure S43.** IC_50_ curve of compounds **6** against drug-resistant HCC827 cells (A: Gefitinib-resistant, B: Osimertinib-resistant) cell lines
